# FGF signaling acts on different levels of mesoderm development within Spiralia

**DOI:** 10.1242/dev.196089

**Published:** 2021-05-17

**Authors:** Carmen Andrikou, Andreas Hejnol

**Affiliations:** 1 University of Bergen, Department of Biological Sciences, Thormøhlensgate 55, 5006 Bergen, Norway; 2 Sars International Centre for Marine Molecular Biology, University of Bergen, Thormøhlensgate 55, 5006 Bergen, Norway

**Keywords:** Lophophorate, Axial elongation, Gastrulation, Evolution, Sprouty

## Abstract

FGF signaling is involved in mesoderm induction in members of deuterostomes (e.g. tunicates, hemichordates), but not in flies and nematodes, in which it has a role in mesoderm patterning and migration. However, we need comparable studies in other protostome taxa in order to decipher whether this mesoderm-inducing function of FGF extends beyond the lineage of deuterostomes. Here, we investigated the role of FGF signaling in mesoderm development in three species of lophophorates, a clade within the protostome group Spiralia. Our gene expression analyses show that the mesodermal molecular patterning is conserved between brachiopods and phoronids, but the spatial and temporal recruitment of transcription factors differs significantly. Moreover, the use of the inhibitor SU5402 demonstrates that FGF signaling is involved in different steps of mesoderm development, as well as in morphogenetic movements of gastrulation and axial elongation. Our findings suggest that the mesoderm-inducing role of FGF extends beyond the group of deuterostomes.

## Abstract (precis)

**Summary:** Spatial and temporal differences in lophophorate mesoderm development are observed in Spiralia, which suggests a possible diversification of mesodermal developmental programs, despite an overall conserved role of FGF signaling in mesoderm formation.

## INTRODUCTION

**Fig. 1.**
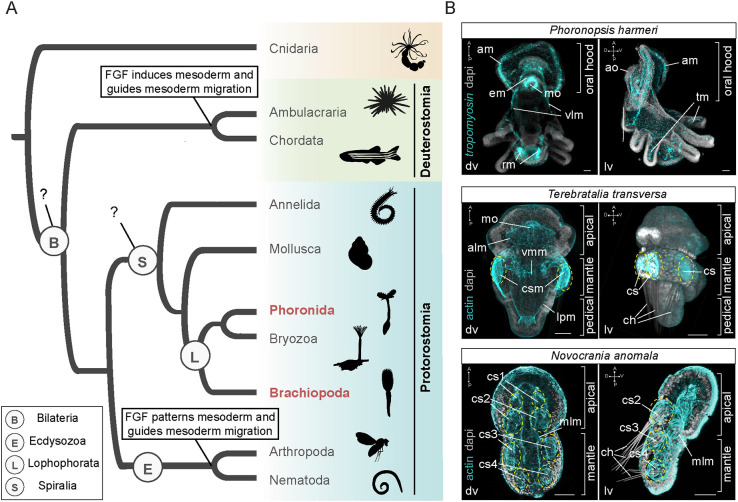
**The distinct roles of FGF signaling in mesoderm development among bilaterians.** (A) FGF signaling plays pivotal roles in mesoderm induction and migration in members of deuterostomes; however, in protostomes the information is restricted to members of ecdysozoans, where it acts in mesoderm patterning and migration. Animal illustrations are taken from phylopic.org where they were published under a CC-BY 3.0 license. (B) Morphology of musculature in larvae of three representative lophophorate species: the brachiopods *Terebratalia transversa* and *Novocrania anomala*, and the phoronid *Phoronopsis harmeri.* In brachiopods muscles are stained by immunohistochemistry against actin and in *Ph. harmeri* musculature is stained by *tropomyosin* gene expression. In brachiopods, the coelomic sacs are encircled by yellow dashed lines. Every fluorescent image is a *z*-projection of merged confocal stacks and nuclei are stained with DAPI. Anterior to the top. alm, apical longitudinal muscles; am, anterior muscles; ao, apical organ; ch, chaetae; cs, coelomic sac; csm, coelomic sac muscles; dv, dorsoventral view; em, esophageal muscles; lpm, lateral pedicle muscles; lv, lateral view; mlm, mediolateral muscles; mo, mouth; rm, retractor muscles; tm, tentacular muscles; vlm, ventrolateral muscles; vmm, ventral lateral mantle muscles. Scale bars: 20 µm.

Mesoderm is an embryonic germ layer of bilaterians that gives rise to tissues residing between the ectoderm and endoderm, such as coeloms and muscles (Hyman, 1951; Ruppert, 1991). The way mesoderm is formed varies between embryos of different species. Mesoderm can originate by outpouchings of the invaginating endoderm, for example in most deuterostomes (Franz, 1924; Hennig, 1984; Hyman, 1955, 1959; Swalla, 1993) and two clades of protostomes – the Chaetognatha (Hertwig, 1896; Kapp, 2000; Matus et al., 2006) and the Brachiopoda (Conklin, 1902; Kowalevsky, 1874; Plenk, 1913). Alternatively, mesoderm can form by delamination of one or more precursor cells that internalize during gastrulation, for example in spiralian species, where the source of mesoderm can be of endodermal (e.g. the micromere 4d) and ectodermal origin (e.g. micromeres from the animal pole/anterior end of the blastopore) (summarized by Henry and Martindale, 1999; Kozin and Kostyuchenko, 2016; Lambert, 2008; Lyons and Henry, 2014), and in ecdysozoans, where mesoderm originates either from internalization of vegetal endomesodermal cells (Martin-Duran and Hejnol, 2015; Sulston et al., 1983) or from cells of the blastoderm (Eriksson and Tait, 2012; Hartenstein et al., 1985). Despite the differences in the embryological origin and morphogenesis, the molecular underpinnings of mesoderm induction, migration and differentiation into various derivatives shares similarities within bilaterians (Amin et al., 2009, 2010; Andrikou et al., 2013; Chiodin et al., 2013; Fritzenwanker et al., 2014; Grifone et al., 2005; Harfe et al., 1998; Hinman and Degnan, 2002; Imai et al., 2004; Kozin et al., 2016; Kozmik et al., 2007; Mahlapuu et al., 2001; Mankoo et al., 1999; Materna et al., 2013; Nederbragt et al., 2002; Osborne et al., 2018; Passamaneck et al., 2015; Perry et al., 2015; Rudnicki et al., 1993; Sandmann et al., 2007; Schubert et al., 2003; Shimeld et al., 2010; Zaffran et al., 2001) (Table S1). These molecular similarities have been commonly used as an argument for the homology of this germ layer (Burton, 2008; Lartillot et al., 2002; Martindale et al., 2004; Seipel and Schmid, 2005; Technau and Scholz, 2003). In addition to shared sets of transcriptions factors, conserved signaling cascades are also involved in different steps of mesoderm development, such as fibroblast growth factor (FGF), Notch and bone morphogenetic protein (BMP) (Good et al., 2004; Itoh and Ornitz, 2004; Sweet et al., 1999; Wijesena et al., 2017; Winnier et al., 1995) (Table S1). FGF signaling is of particular interest due to its proposed ancestral role in mesoderm induction in deuterostomes (Fan et al., 2018; Green et al., 2013). Functional studies have demonstrated that this signal is required for posterior mesoderm formation in vertebrates (Amaya et al., 1993; Draper et al., 2003; Fletcher et al., 2006; Fletcher and Harland, 2008; Yamaguchi et al., 1994), anterior mesoderm formation in cephalochordates (Bertrand et al., 2011), mesenchyme induction and formation of notochord, trunk ventral cells (TVC) and tail muscle in tunicates (Davidson et al., 2006; Imai et al., 2002; Kim and Nishida, 2001; Yasuo and Hudson, 2007), mesoderm induction in hemichordates (Fan et al., 2018; Green et al., 2013) and myoblast formation in sea urchins (Andrikou et al., 2015). Outside deuterostomes, however, studies addressing the role of FGF in mesoderm development are scarce. The only available data among protostome taxa concerns the two well-studied ecdysozoans *Drosophila melanogaster* and *Caenorhabditis elegans*, in which FGF is involved in mesoderm patterning and migration but not in induction (Beiman et al., 1996; Burdine et al., 1998; DeVore et al., 1995; Kadam et al., 2009; Lo et al., 2008; McMahon et al., 2010; Photos et al., 2006; Stathopoulos et al., 2004; Sun and Stathopoulos, 2018; Wilson et al., 2005). A question therefore emerges as to whether the mesoderm-inducing role of FGF originated within deuterostomes, or predated deuterostomes and was lost in the lineage of ecdysozoans (Fig. 1A). To gain insight into the ancestral role of FGF signaling for mesoderm development, data from other protostomes and, in particular, members of the Spiralia, are therefore needed.

Lophophorates, comprised of Bryozoa, Brachiopoda and Phoronida (Kocot et al., 2017; Laumer et al., 2019), belong to the lineage of Spiralia (Fig. 1A). These animals exhibit ‘deuterostome-like’ features in their development, such as radial cleavage and enterocoely (Zimmer, 1997). We used two brachiopod species, the rhynchonelliform *Terebratalia transversa* and the craniiform *Novocrania anomala*, and one phoronid species, *Phoronopsis harmeri* (Fig. 1B), which show profound differences in mesoderm development such as the time and site of mesoderm emergence, the direction of mesoderm migration and the degree of mesoderm compartmentalization and differentiation. In particular, in *T. transversa* mesoderm is specified at the blastula stage, whereas the mesoderm of *N. anomala* and *Ph. harmeri* forms at the gastrula stage (Andrikou et al., 2019; Martín-Durán et al., 2017; Passamaneck et al., 2015). In addition, in *T. transversa* and *Ph. harmeri* the mesodermal tissue migrates in an anterior-to-posterior direction, but in *N. anomala* it follows a posterior-to-anterior direction (Andrikou et al., 2019; Freeman, 1993, 2000, 2003; Martín-Durán et al., 2017; Nielsen, 1991; Passamaneck et al., 2015; Rattenbury, 1954; Temereva and Malakhov, 2007). Finally, in *T. transversa*, mesoderm differentiates into musculature, which consists of an anterior domain in the apical lobe (apical longitudinal muscles), an umbrella-like domain in the mantle lobe (ventral lateral mantle muscles) that projects to four coelomic sacs with chaetae bundles, and a posterior domain in the pedicle lobe (lateral pedicle muscles) (Altenburger and Wanninger, 2009; Freeman, 2003; Martín-Durán et al., 2017; Passamaneck et al., 2015; Vellutini and Hejnol, 2016); in *N. anomala* mesoderm differentiates into four pairs of coelomic sacs – with the three posterior ones projecting into chaetae bundles – and mediolateral muscles (Altenburger and Wanninger, 2010; Freeman, 2000; Martín-Durán et al., 2017; Nielsen, 1991; Vellutini and Hejnol, 2016); in *Ph. harmeri* the mesodermal derivatives can be distinguished as an anterior domain with a pre-oral coelom and projecting ventrolateral muscles, circumesophageal muscles and tentacular muscles, and a posterior domain that emerges at the larva stage and includes a trunk coelom (metacoel) and retractor muscles (Andrikou et al., 2019; Rattenbury, 1954; Temereva and Malakhov, 2007) (Fig. 1B). We investigated and compared the molecular mechanisms of mesoderm development in these three species, with an emphasis on the role of the FGF signaling pathway. Our results suggest an overall conserved involvement of FGF in mesoderm migration and differentiation in all three lophophorate species tested. Moreover, they show a similar mesoderm-inducing role of this signal in *Ph. harmeri* to members of deuterostomes.

## RESULTS

### The spatiotemporal expression of mesodermal markers during development differs between *N. anomala*, *T. transversa* and *Ph. harmeri*

**Fig. 2.**
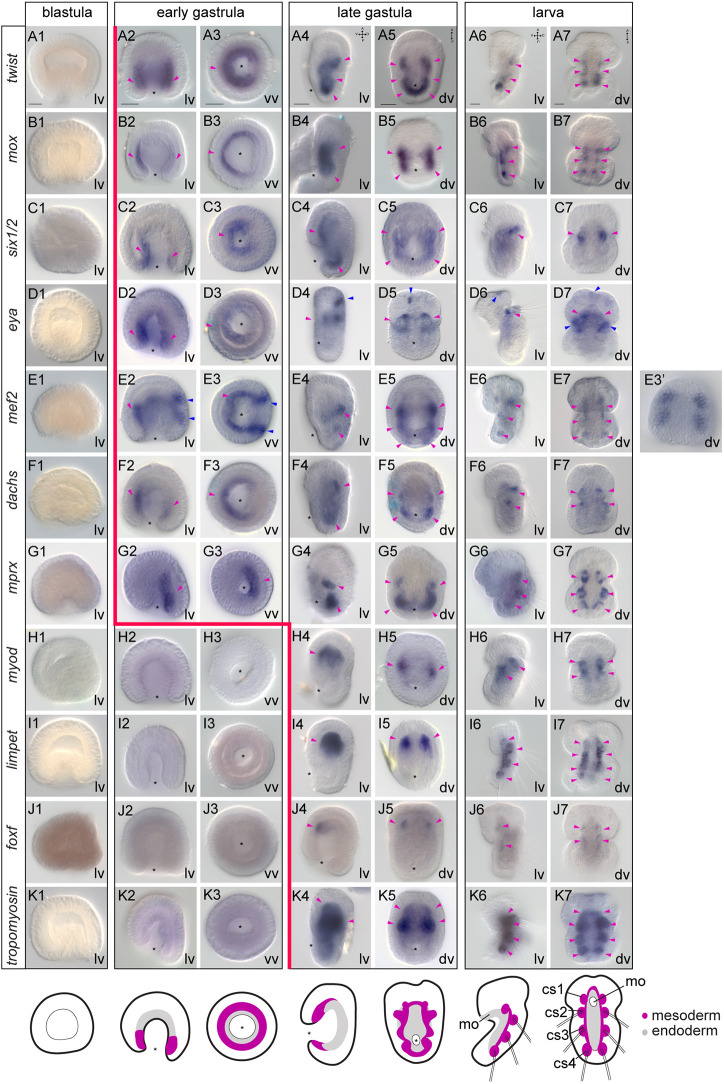
**Mesodermal gene expression during *Novocrania anomala* development.** (A-K) Whole-mount *in situ* hybridization of *twist* (A), *mox* (B), *six1/2* (C), *eya* (D), *mef2* (E), *dachs* (F), *mprx* (G), *myod* (H), *limpet* (I), *foxf* (J) and *tropomyosin* (K) in blastulae, early gastrulae, late gastrulae and larvae of *N. anomala*. On the right, panel E3′ shows a different focal plane of the embryo. The position of the blastopore is indicated with an asterisk. Magenta arrowheads indicate mesodermal domains and derivatives, in which gene expression is detected, and blue arrowheads indicate ectodermal expression. Red line marks the onset of mesodermal gene expression. Anterior to the top. cs, coelomic sac; dv, dorsoventral view; lv, lateral view; mo, mouth; vv, vegetal view. Scale bars: 20 μm.

**Fig. 3.**
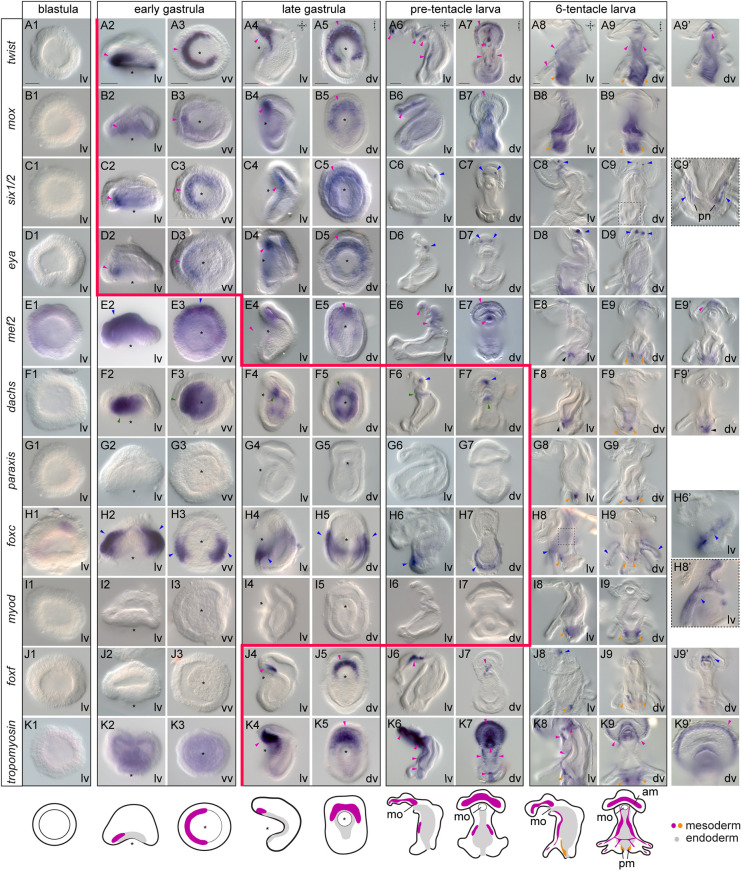
**Mesodermal gene expression during *Phoronopsis harmeri* development.** (A-K) Whole-mount *in situ* hybridization of *twist* (A), *mox* (B), *six1/2* (C), *eya* (D), *mef2* (E), *dachs* (F), *paraxis* (G), *foxc* (H), *myod* (I), *foxf* (J) and *tropomyosin* (K) in blastulae, early gastrulae, late gastrulae, pre-tentacle larvae and 6-tentacle larvae of *Ph. harmeri*. On the right, panels A9′, E9′, F9′, H6′, J9′ and K9′ show different focal planes of the embryos, and panels C9′ and H8′ show higher magnifications of the boxed areas in C9 and H8, respectively. The position of the blastopore is indicated with an asterisk. Magenta arrowheads indicate anterior mesodermal domains and derivatives, in which gene expression is detected. Blue arrowheads indicate ectodermal expression. Green arrowheads show endodermal expression. In the 6-tentacle larvae, orange arrowheads indicate expression in the posterior domain and black arrowheads show expression in the metasomal sac. Red line marks the onset of mesodermal gene expression. Anterior to the top. am, anterior muscle; dv, dorsoventral view; lv, lateral view; pm, posterior muscle; pn, protonephridia; mo, mouth; vv, vegetal view. Scale bars: 20 μm.

To understand whether the developmental and morphological variations of mesoderm formation between these three species are associated with differences in molecular patterning, we first revealed the expression of the conserved mesodermal transcription factors *twist*, *mox*, *six1/2*, *eya*, *mef2*, *dachs*, *paraxis*, *foxc*, *mprx*, *myod*, *limpet*, *foxf* and *nk1* (Table S1) in *N. anomala* and *Ph. harmeri* (Figs 2, 3; Figs S1, S2). The mesodermal expression of these genes during development has been previously described in *T. transversa* (Passamaneck et al., 2015). All genes, with the exception of *nk1* (in *N. anomala*) (Fig. S2A), showed mesodermal expression. The earliest mesodermal marker is *twist*, expression of which initiates at the early gastrula stage and demarcates the entire mesoderm in both species (Figs 2A2,A3, 3A2,A3) (Andrikou et al., 2019; Martín-Durán et al., 2017), indicating that mesoderm is specified before its morphological separation from endoderm. *Mox*, *six1/2* and *eya*, genes commonly involved in mesoderm patterning, are expressed shortly after (Figs 2B2,B3,C2,C3,D2,D3, 3B2,B3,C2,C3,D2,D3). Transcripts of transcription factors often associated with muscle development, such as *myod*, *limpet* (only in *N. anomala*), *foxf* (Martín-Durán et al., 2017) and the terminal differentiation gene *tropomyosin*, begin to be detected at the late gastrula stage (Figs 2H4,H5,I4,I5,J4,J5,K4,K5, 3J4,J5,K4,K5) in both organisms, correlating with the formation of musculature (Altenburger and Wanninger, 2010; Temereva and Tsitrin, 2013).

However, when comparing these results between the three organisms, the onset of expression of a number of orthologs varies (Fig. S3). For example, in *T. transversa* the expression of *mox* and *eya* only starts at the late gastrula stage (Passamaneck et al., 2015), although mesoderm (*twist*-positive cells) is already present from the blastula stage (Martín-Durán et al., 2017). Also, *mef2* shows an early mesodermal expression in *N. anomala* (Fig. 2E2,E3), but in *T. transversa* (Passamaneck et al., 2015) and *Ph. harmeri* (Fig. 3E4,E5) this gene is not mesodermally activated before the late gastrula stage. A second important difference concerns the spatial patterning of the different subpopulations of mesodermal derivatives (Fig. S3). *Twist* is expressed in both anterior and posterior regions in *Ph. harmeri* (Fig. 3A4-A9), in *N. anomala* it is expressed in three pairs of coelomic sacs (cs2-cs3-cs4) but acquires a stronger expression in the most posterior (cs4) (Fig. 2A6,A7) and, in *T. transversa*, *twist* expression is confined to the anterior (apical) region and mantle region (which includes also the coelomic sacs) (Passamaneck et al., 2015). The expression of *six1/2* and *myod* is excluded from the posterior (cs4) pairs of coelomic sacs in *N. anomala* (Fig. 2C6,C7,H6,H7), whereas in *T. transversa* these genes are expressed in both the anterior (apical) and posterior (pedicle) regions (Passamaneck et al., 2015). In *Ph. harmeri*, *six1/2* (Fig. 3C2-C5) and *myod* (Fig. 3I8,I9; Fig. S2B) are restricted to the anterior and posterior regions, respectively. Moreover, *mprx* is solely expressed in the mantle region of *T. transversa* (Passamaneck et al., 2015), whereas in *N. anomala* the orthologous gene is expressed in the three most posterior pairs of coelomic sacs (cs2-cs3-cs4) (Fig. 2G6,G7). *Foxf* and *foxc* expression is confined to the anterior regions in both brachiopod species (Fig. 2J4-J7; Fig. S3) (Passamaneck et al., 2015; Martín-Durán et al., 2017), whereas in *Ph. harmeri*, *foxf* is expressed in both the anterior and posterior regions (Fig. 3J4-J9) and transcripts of *foxc* are only detected in the posterior region (Fig. 3H8,H9). *Dachs* is not expressed in the anterior region in *Ph. harmeri* (Fig. 3F2-F9), whereas in *T. transversa* it demarcates the entire musculature (Passamaneck et al., 2015) and in *N. anomala* is confined to the anterior pairs of coelomic sacs (cs2-cs3) (Fig. 2F6,F7). Finally, *hox3* was previously described to be expressed in the mantle region (*T. transversa*) and the posterior pair of coelomic sacs (c4) (*N. anomala*) in brachiopods (Schiemann et al., 2017), but this is not the case for *Ph. harmeri*, as the orthologous gene is solely expressed in the metasomal sac and not in the mesoderm (Gąsiorowski and Hejnol, 2020)*.* These data show that, in all three organisms, mesoderm development exhibits differences not only in the recruitment of transcription factors, but also in their temporal and spatial expression profiles, suggesting diverse underlying patterning mechanisms.

### Gene expression of FGF signaling components suggest their possible association with mesoderm and neuroectoderm development

**Fig. 4.**
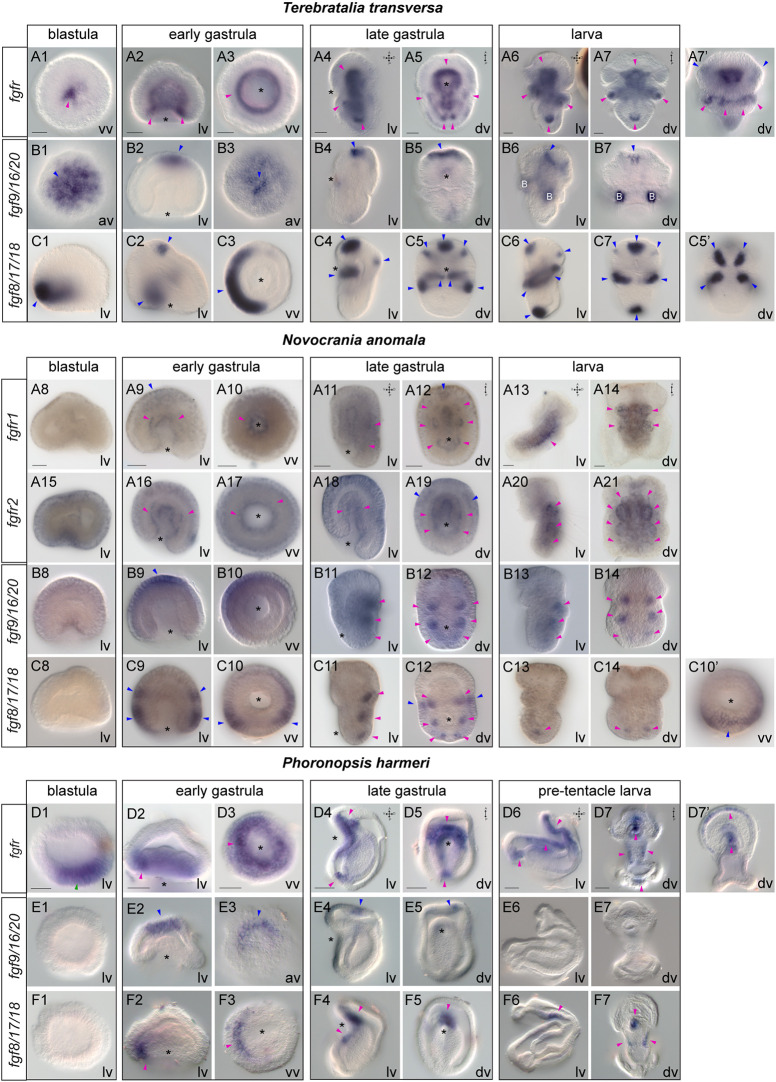
**Gene expression of FGF signaling components in lophophorates.** (A-F) Whole-mount *in situ* hybridization of *fgfr* (A,D) , *fgf9/16/20* (B,E) and *fgf8/17/18* (C,F) during the blastula, early gastrula, late gastrula and larva stages of development of *Terebratalia transversa*, *Novocrania anomala* and *Phoronopsis harmeri*. On the right, panels A7′, C5′, C10′ and D7′ show different focal planes of the embryos. The position of the blastopore is indicated with an asterisk. Magenta arrowheads indicate mesodermal domains and derivatives, in which gene expression is detected. Blue arrowheads indicate ectodermal expression. Green arrowheads show endodermal expression. Anterior to the top. av, animal view; B, background staining; dv, dorsoventral view; lv, lateral view; vv, vegetal view. Scale bars: 20 μm.

We then searched for components of the FGF signaling pathway. Two FGF receptors were found in *N. anomala* but only one in *T. transversa* and *Ph. harmeri* (Fig. S1). Moreover, all three animals possess one copy of FGF9/16/20 and FGF8/17/18 ligands (Fig. S1). In *T. transversa*, *fgfr* is expressed in a few cells of the vegetal pole at the blastula stage (Fig. 4A1). In early gastrulae, transcripts of the gene demarcate the invaginating endomesoderm (Fig. 4A2,A3) and this expression is retained at the late gastrula stage, in the archenteron, the anterior and posterior mesoderm and the developing coelomic sacs (Fig. 4A4,A5). In larvae, *fgfr* is additionally activated in two anterior-lateral ectodermal patches (Fig. 4A6,A7). The two ligands also exhibit a very distinct expression from each other. *Fgf9/16/20* is expressed in a few cells of the animal pole from the blastula stage up to the larva stage (Fig. 4B1-B7). In contrast, *fgf8/17/18* (Vellutini and Hejnol, 2016) starts to be expressed at the blastula stage in an anterior-ventral ectodermal half ring (Fig. 4C1), whereas in early gastrulae, transcripts of the gene are detected in transverse ventral bands reaching the anterior domain of the blastopore, and the future apical organ (Fig. 4C2,C3). In late gastrulae, *fgf8/17/18* is expressed in two medio-lateral spots, which correspond to the developing coelomic sacs, in two anterior-ventral cellular patches, the apical organ, as well as in one ventral pair of spots proximal to the mouth and another dorsal pair. Also, a new domain of expression at the posterior tip is activated (Fig. 4C4,C5). Finally, in larvae, the ventral expression of *fgf8/17/18* fades and the gene is only expressed anteriorly, in the coelomic sacs and the posterior tip (Fig. 4C6,C7). The analysis of the spatial expression of the three receptors and the two ligands suggests a putative involvement of FGFR and FGF8/17/18 in mesoderm development (see relative expression of *fgfr* and *fgf8/17/18* in Fig. S4).

In *N. anomala*, none of the FGF signaling components is expressed at the blastula stage, which differs from what is observed in *T. transversa* (Fig. 4A8,A15,B8,C8)*.* The expression of both FGF receptors is detected at the early gastrula stage, in the invaginating archenteron and the invaginating mesoderm (Fig. 4A9,A10,A16,A17). In addition, transcripts of *fgfr1* are found in the anterior ectoderm (Fig. 4A9). In late gastrulae, *fgfr1* is expressed in anterior ectodermal cells, the developing coelomic sacs and the tip of the archenteron (Fig. 4A11,A12). At the larva stage, *fgfr1* expression is confined to two anterior pairs (cs2-cs3) of coelomic sacs (Fig. 4A13,A14). *Fgfr2* is mainly expressed in the forming archenteron and the developing coelomic sacs, as well as in two anterior-lateral ectodermal patches at the late gastrula stage (Fig. 4A18,A19). Finally, in larvae, *fgfr2* is expressed in all four pairs of coelomic sacs (cs1-cs2-cs3-cs4) (Fig. 4A20,A21). The expression of two ligands also begins during gastrulation. *Fgf9/16/20* expression is initially detected in the anterior ectoderm in early gastrulae (Fig. 4B9,B10), but in late gastrulae and larvae the ectodermal expression fades and a new mesodermal domain appears in three pairs (cs2-cs3-cs4) of coelomic sacs (Fig. 4B11-B14). *Fgf8/17/18* (Vellutini and Hejnol, 2016) is expressed in two ectodermal bands that encircle the early gastrula, one more posterior near the blastopore and another at the middle part of the embryo (Fig. 4C9,C10). In late gastrulae, transcripts of the gene are detected in three developing pairs of coelomic sacs (cs2-cs3-cs4) and the ectodermal patches adjacent to the second pair (cs2) (Fig. 4C11,C12), whereas at the larva stage the expression of *fgf8/17/18* is restricted to the most posterior pair (cs4) of coelomic sacs (Fig. 4C13,C14). Based on their expression, these data suggest that both receptors and ligands are possibly related to mesoderm development in *N. anomala*.

In *Ph. harmeri*, the FGF receptor is already expressed at the blastula stage, in cells of the vegetal pole (presumptive endomesoderm) (Fig. 4D1). At the early gastrula stage, the gene is expressed in an anterior ventro-lateral cell population of the vegetal plate, the presumptive mesoderm, the anterior blastoporal lip, as well as the anterior ectoderm (Fig. 4D2,D3). In late gastrulae, the gene is expressed in anterior migrating mesodermal cells and a posterior cell cluster located adjacent to the developing intestine (Fig. 4D4,D5). At the pre-tentacle larva stage, the expression of *fgfr* remains in clusters of cells of the pre-oral mesoderm, two ventrolateral muscle tiers, the posterior cell cluster, the ventral ectoderm and the apical organ (Fig. 4D6,D7). *Fgf9/16/20* and *fgf8/17/18* exhibit very different expression profiles. *Fgf9/16/20* is transiently expressed in the forming apical organ until the late gastrula stage (Fig. 4E2-E5). *Fgf8/17/18* exhibits a more dynamic expression, transcripts are detected in the anterior lip of the blastopore, the anterior-ventral ectoderm and the anterior endoderm in early gastrulae (Fig. 4F2,F3), whereas in late gastrulae and pre-tentacle larvae, *fgf8/17/18* is expressed in the anterior-ventral ectoderm of the oral hood, a posterio-ventral group of ectodermal cells and the mouth (Fig. 4F4-F7). These data show that in *Ph. harmeri* too, the expression of FGFR and FGF8/17/18 is possibly associated with mesoderm formation (see relative expression of *fgfr* and *fgf8/17/18* in Fig. S4). Overall, FGF signaling is likely involved in mesoderm development and neuroectodermal patterning in all three organisms. A summary of the expression of the FGF signaling components in *N. anomala*, *T. transversa* and *Ph. harmeri* is provided in Fig. S5.

### Sprouty genes are downstream of FGF signaling pathway in lophophorates

**Fig. 5.**
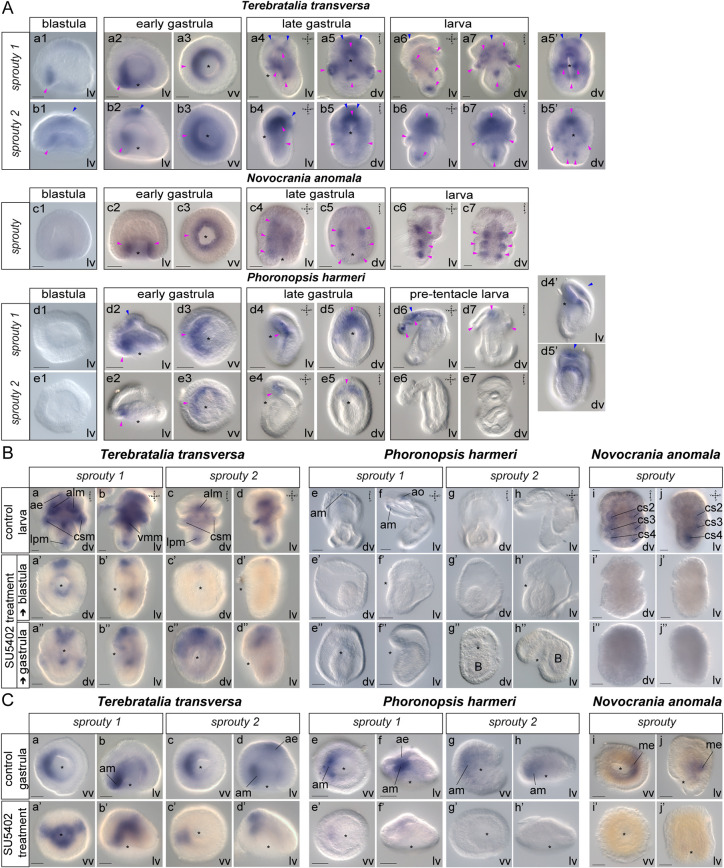
**Gene expression of sprouty genes during the development of *Terebratalia transversa*, *Novocrania anomala* and *Phoronopsis harmeri* and after SU5402 treatments.** (A) Whole-mount *in situ* hybridization (WMISH) of sprouty genes during the blastula, early gastrula, late gastrula and larva stages of the development of *T. transversa* (Aa,Ab), *N. anomala* (Ac) and *Ph. harmeri* (Ad,Ae). Magenta arrowheads indicate mesodermal domains and derivatives, in which gene expression is detected. Blue arrowheads indicate ectodermal expression. On the right, panels Aa5′, Ab5′, Ad4′ and Ad5′ show different focal planes of the embryos. (B) WMISH of sprouty genes in *T. transversa* (Ba-Bd), *N. anomala* (Bi,Bj) and *Ph. harmeri* (Be-Bh) blastula and gastrula embryos treated with 20 µM SU5402 and fixed at the larva stage. (C) WMISH of sprouty genes in *T. transversa* (Ca-Cd), *N. anomala* (Ci,Cj) and *Ph. harmeri* (Ce-Ch) blastula embryos treated with 20 µM SU5402 and fixed at the gastrula stage. The position of the blastopore is indicated with an asterisk. Anterior to the top. ae, anterior ectoderm; alm, apical longitudinal muscles; am, anterior mesoderm; B, background staining; cs, coelomic sac; csm, coelomic sac muscles; dv, dorsoventral view; lpm, lateral pedicle muscles; lv, lateral view; me, mesoderm; vmm, ventral lateral mantle muscles; vv, vegetal view. Scale bars: 20 μm.

We next looked for putative downstream modulators of FGF signaling. We hypothesized that Sprouty family members may be involved in regulating FGF signal interpretation in lophophorates, as previously shown in members of vertebrates and the fruit fly (Hacohen et al., 1998; Minowada et al., 1999). These genes can be downstream antagonists (Casci et al., 1999) or enhancers (Wong et al., 2002) of the MAPK pathway, a major downstream target of FGF signaling (Ornitz and Itoh, 2015). Two sprouty genes were found in *T. transversa* and *Ph. harmeri*, and only one in *N. anomala* (Fig. S1). In all three species the expression of sprouty genes resembled the expression of fgfr genes, both in a spatial and a temporal manner (Fig. 5A), suggesting that these genes might have a regulatory effect on FGF signaling.

We then treated the embryos with SU5402, a selective inhibitor of FGFR (Mohammadi et al., 1997) (Fig. 5B,C) (for a summary of treatments see Fig. S6). When we tested the expression of sprouty genes in larvae treated from the blastula stage, a severe reduction in the mesoderm and mesodermal derivatives was witnessed in all three species (Fig. 5Ba-Bj′). In addition, *Ph. harmeri* exhibited a loss of ectodermal expression (Fig. 5Be-Bf′). When larvae were treated from the gastrula stage, the mesodermal expression of *sprouty* was partly recovered in *Ph. harmeri* and *T. transversa* (Fig. 5Ba″,Bf″) but this was not the case for *N. anomala* (Fig. 5Bi″,Bj″). Ectodermal expression was also recovered in *Ph. harmeri* (Fig. 5Be″,Bf″).

To understand in which stage of development the expression of sprouty genes was affected, we also tested their expression in gastrulae treated from the blastula stage, where we saw that in *Ph. harmeri* and *N. anomala* the mesodermal expression (and also the ectodermal one, in the case of *Ph. harmeri*) was already abolished (Fig. 5Ce-j′), suggesting an early regulation of FGF signaling on mesoderm development. The same was not true for *T. transversa*, where the expression of both sprouty genes remained unaffected in treated gastrulae (Fig. 5Ca-d′), suggesting that FGF signaling is acting at a later developmental stage in this species. These results suggest that sprouty genes are downstream of FGF signaling in all three investigated species, as shown in other organisms.

### Perturbation of FGF signaling results in failure in mesoderm formation in *N. anomala* and *T. transversa*

**Fig. 6.**
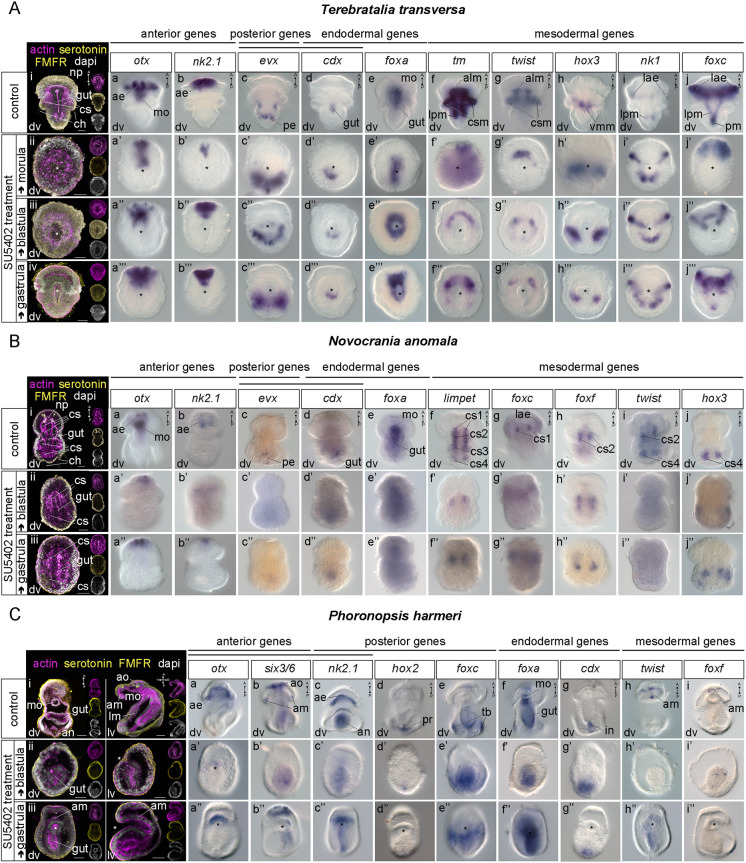
**SU5402 treatments in *Terebratalia transversa*, *Novocrania anomala* and *Phoronopsis harmeri*.** (A) Left: immunohistochemistry of markers of the nervous system (serotonin, FMFRamide; yellow) and musculature (actin; magenta) in *T. transversa* control (i) and morula (ii), blastula (iii) and gastrula (iv) embryos treated with 20 µM SU5402 and fixed at the larva stage. Right: whole-mount *in situ* hybridization (WMISH) of anterior [*otx* (Aa), *nk2.1* (Ab), *foxc* (Aj), *nk1* (Ai)], posterior [*evx* (Ac)], endodermal [*foxa* (Ae), *cdx* (Ad)] genes, and markers of musculature [*tropomyosin*; *tm* (Af)], apical longitudinal and coelomic sac muscles [*twist* (Ag)], ventral mantle lateral muscles [*hox3* (Ah)] and pedicle muscles [*nk1* (Ai), *foxc* (Aj)] in *T. transversa* control and morula, blastula and gastrula embryos treated with 20 µM SU5402 and fixed at the larva stage. (B) Left: immunohistochemistry of markers of the nervous system (serotonin, FMFRamide; yellow) and musculature (actin; magenta) in *N. anomala* control (i) and blastula (ii) and gastrula (iii) embryos treated with 20 µM SU5402 and fixed at the larva stage. Right: WMISH of anterior [*otx* (Ba), *nk2.1* (Bb), posterior genes [*evx* (Bc)], endodermal [*foxa* (Be), *cdx* (Bd)] genes, and markers of the entire musculature [*limpet* (Bf)], anterior coelomic sacs [*foxf* (Bh), *foxc* (Bg)] and posterior coelomic sacs [*twist* (Bi), *hox3* (Bj)] in *N. anomala* control and blastula and gastrula embryos treated with 20 µM SU5402 and fixed at the larva stage. (C) Left: immunohistochemistry of markers of the nervous system (serotonin, FMFRamide; yellow) and musculature (actin; magenta) in *Ph. harmeri* control (i) and blastula (ii) and gastrula (iii) embryos treated with 20 µM SU5402 and fixed at the larva stage. Right: WMISH of anterior [*otx* (Ca), *six3/6* (Cb), *nk2.1* (Cc)], posterior [*nk2.1* (Cc), *hox2* (Cd)], posterio-ventral [*foxc* (Ce)], endodermal [*foxa* (Cf), *cdx* (Cg), *nk2.1* (Cc)] and markers of musculature [*twist* (Ch), *six3/6* (Cb), *foxf* (Ci)] in *Ph. harmeri* control and blastula and gastrula embryos treated with 20 µM SU5402 and fixed at the larva stage. Every fluorescent image is a *z*-projection of merged confocal stacks and nuclei are stained with DAPI. The position of the blastopore is indicated with an asterisk. All samples represent at least two biological and two technical replicates (*n*=10). Lateral views of larvae are shown in Fig. S8. Anterior to the top. ae, anterior ectoderm; alm, apical longitudinal muscles; am, anterior muscles; an, anus; ao, apical organ; ch, chaete; cs, coelomic sac; csm, coelomic sac muscles; dv, dorsoventral view; in, intestine; lae, lateral anterior ectoderm; lm, lateral muscles; lpm, lateral pedicle muscles; mo, mouth; np, neuropile; pe, posterior ectoderm; pm, pedicle muscles; pr, protonephridial rudiment; tb, tentacle bulbs; vmm, ventral lateral mantle muscles. Scale bars: 20 µm.

Based on the mesoderm-related expression of FGF signaling components and sprouty genes we hypothesized that FGF might be involved in mesoderm development in brachiopods. To test this, we treated embryos at different developmental stages with SU5402 (for a summary of treatments and phenotypes see Figs S6 and S7). SU5402 treatment abolished the formation of coelomic sacs, chaetae bundles and neuropile in larvae of both brachiopod species (Fig. 6A,B; Fig. S8A,B).

*T. transversa* treated larvae were not compartmentalized in apical, mantle and pedicle lobes as in controls (Fig. 6Ai), but instead remained spherical with an open blastopore when we treated them from the blastula stage (Fig. 6Aiii). The chaetae bundles were not formed, and the musculature was severely malformed (Fig. 6Aiii). The phenotype was milder in animals treated from a later developmental stage (mid gastrula), with the blastopore migrating more anteriorly and the lobes more prominent (Fig. 6Aiv). To ensure that we inhibited FGFR before mesoderm originated, we also treated larvae from the morula stage, which resulted in spherical embryos with a barely formed blastopore (Fig. 6Aii).

To understand whether this truncated phenotype was due to a failure of axial elongation or a disruption of the anterior-posterior patterning, we tested the expression of anterior (*otx*, *nk2.1*) (Fig. 6Aa-a‴,Ab-b‴) and posterior ectodermal markers (*evx*) (Martín-Durán et al., 2017) (Fig. 6Ac-c‴) and found them unaffected. The expression of the endodermal markers *cdx* and *foxa* (Martín-Durán et al., 2017) was also unaltered (Fig. 6Ad-d‴,Ae-e‴). We then examined the treated animals for the muscle differentiation marker *tropomyosin* (Passamaneck et al., 2015) and a loss of posterior expression was observed (Fig. 6Af-f‴). Interestingly, in larvae treated from the gastrula stage, *tropomyosin* expression was extended slightly more posteriorly compared with the ones treated from the blastula stage (Fig. 6Af‴). We also tested the expression of the mesodermal transcription factor *twist*, a marker of the anterior/apical region (Passamaneck et al., 2015) (Fig. 6Ag-g‴), *hox3*, a marker of the mid/mantle region (Schiemann et al., 2017) (Fig. 6Ah-h‴) and *nk1*, a marker of the posterior/pedicle region (Passamaneck et al., 2015) (Fig. 6Ai-i‴), and found them unchanged. Interestingly, the expression of *foxc*, a marker of the most posterior region (Passamaneck et al., 2015) was lost (Fig. 6Aj-j″), suggesting a role of this gene in a later, differentiation step of mesoderm development (see also Fig. S9). In larvae treated from the gastrula stage, the posterior expression of *foxc* was recovered (Fig. 6Aj‴).

These results suggest that in *T. transversa*, FGF signaling is involved in neuropile formation, coordination of morphogenetic movements of gastrulation, and axial elongation. It does not have a role in mesoderm induction and early formation (see also Fig. S10), but instead it appears to be involved in mesoderm migration and differentiation. In *T. transversa* the direction of axial elongation takes place from anterior-dorsal to posterior (Freeman, 1993; Martín-Durán et al., 2017), so the inhibition in axial elongation is probably coupled with the failure in mesoderm migration and differentiation.

*N. anomala* treated animals did not exhibit the same phenotype that we observed in *T. transversa* (Fig. 6B)*.* Larvae treated from the blastula stage (Fig. 6Bii) were smaller than the controls (Fig. 6Bi), but were not spherical, and they possessed an elongated archenteron without a mouth opening (Fig. 6Bii). Also, mesoderm differentiation was severely impaired; the coelomic sacs were malformed and none of the chaetae bundles were formed (Fig. 6Bii). Similar results were obtained when the embryos were treated from the gastrula stage (Fig. 6Biii).

When we looked at the anterior-posterior patterning genes in *N. anomala* (Martín-Durán et al., 2017), we observed an apical reduction of the ectodermal genes *otx* and *nk2.1* (Fig. 6Ba-a″,Bb-b″) and a complete loss of expression of *otx* in the mouth region (Fig. 6Ba-a″), consistent with the fact that the mouth was not formed. The most ectodermal posterior fate was also impaired, as shown from the reduced expression of *evx* (Fig. 6Bc-c″)*.* The expression of the endodermal markers *cdx* and *foxa* (Martín-Durán et al., 2017), however, remained unaffected (Fig. 6Bd-d″,Be-e″). We then tested the expression of *limpet*, a pan-mesodermal differentiation marker in this species. We saw a severe reduction of expression and detection in only one pair of coelomic sacs (Fig. 6Bf-f″). The expression of the marker of the anterior pair of coelomic sacs (cs2) *foxf* (Martín-Durán et al., 2017) was not affected (Fig. 6Bh-h″), but instead transcripts of *foxc* (Martín-Durán et al., 2017), which, in control larvae, are confined to the most anterior pair of coelomic sacs (cs1), were lost (Fig. 6Bg-g″), suggesting that the formation of cs1 pair was abolished. Finally, the expression of *twist* (Martín-Durán et al., 2017) and the marker of the posterior pair of coelomic sacs *hox3* (Schiemann et al., 2017) was inhibited (Fig. 6Bi-i″,Bj-j″). *Hox3* expression was partly recovered when embryos were treated from the gastrula stage onwards (Fig. 6Bj″), indicating that the input of FGF on *hox3* occurs sometime between the blastula and gastrula stage. In order to understand which step of mesoderm development had been compromised, we also tested the expression of the mesodermal marker *twist* in gastrulae treated from the blastula stage on, and found it downregulated, suggesting that in *N. anomala* FGF acts on mesoderm formation earlier in development compared with *T. transversa* (see also Fig. S10).

These data suggest that in *N. anomala*, FGF signaling is involved in anteroposterior patterning, neuropile and neuroectoderm formation, mouth formation, as well as mesoderm migration and differentiation. However, an impact on axial elongation is not evident, as shown in *T. transversa*, further supported by the fact that in *N. anomala* the direction of axial elongation occurs from posterior-ventral to anterior (Freeman, 2000; Martín-Durán et al., 2017; Nielsen, 1991).

### FGF signaling is upstream of mesoderm induction in *Ph. harmeri*

To test the role of FGF signaling in mesoderm development beyond the brachiopod lineage, we also treated *Ph. harmeri* embryos with SU5402 at different developmental stages (for a summary of treatments and phenotypes see Figs S6 and S7). SU5402 treatment from the blastula stage inhibited the formation of musculature and the apical organ of pre-tentacle larvae (Fig. 6C; Fig. S8C). The treated larvae exhibited a truncated phenotype with a short archenteron compared with the controls (Fig. 6Ci,Cii). Treatment from the gastrula stage onwards, resulted in a milder phenotype (Fig. 6Ciii): the blastopore was shifted anteriorly, some mesodermal cells were present and the apical organ was partly recovered (Fig. 6Ciii).

The expression of the anterior muscle markers *six3/6*, *twist* (Andrikou et al., 2019) and *foxf* was abolished (Fig. 6Cb,Cb′,Ch,Ch′,Ci,Ci′), and the same was observed for the markers of the apical organ *six3/6* and *otx* (Andrikou et al., 2019) (Fig. 6Ca,Ca′,Cb,Cb′), in embryos treated from the blastula stage. Similarly, the posterior endodermal expression of *nk2.1* (Fig. 6Cc,Cc′) and the posterior ectodermal expression of *hox2* (Gąsiorowski and Hejnol, 2020) (Fig. 6Cd,Cd′) was downregulated. The tentacular ectodermal expression of *foxc* was dorsally affected (Fig. 6Ce,Ce′). Moreover, the expression of *foxa* (Andrikou et al., 2019) was reduced (Fig. 6Cf,Cf′). In treatments from the gastrula stage on, the expression of the muscle markers *twist*, *six3/6* and *foxf*, the marker of the apical organ *six3/6* and *otx*, the posterior endodermal marker *nk2.1* and the marker of the mouth *foxa* was partly recovered (Fig. 6Ca″,Cb″,Cc″,Cf″,Ch″,Ci″). To test whether the observed mesoderm malformation was due to a failure in mesoderm induction, we also tested the expression of the earliest mesodermal markers *twist* and *six3/6* in gastrulae treated from the blastula stage on, and found it was abolished (Fig. S10).

These results show that FGF signaling is upstream of apical organ formation, gastrulation movements, mesoderm induction and anteroposterior patterning in *Ph. harmeri*. Moreover, additional participation of FGF in mesoderm migration and differentiation (as seen in brachiopods) might occur due to the recovered but undifferentiated mesoderm in larvae treated from the gastrula stage, as well as the observed coexpression of the mesodermal marker *twist* and *fgfr* throughout development (Fig. S11). Additional experiments are needed to test this hypothesis.

Overall, our data suggest a role of FGF signaling in mesoderm development in the lineage of lophophorates. Moreover, a conserved involvement of FGF in anteroposterior patterning, neuroectoderm formation, morphogenetic movements of gastrulation and axial elongation is observed.

## DISCUSSION

### Expression dynamics of the mesodermal gene battery

Nearly all the genes we studied are expressed during mesodermal development in the investigated lophophorate species; however, the temporal expression dynamics and spatial recruitment of some genes differ (Fig. S3). Although *twist*, *six1/2* and *foxf* are expressed in a similar sequential manner in all three organisms, the remaining genes occupy different temporal regulatory positions. The spatial utilization of the genetic repertoire in the differentiated subsets of mesoderm exhibits only a few cases shared in all three species (e.g. *mef2*, which demarcates the entire mesoderm, and *eya*, which is mostly expressed in the anterior mesoderm), but the other genes show differences in their spatial transcript distribution. Overall, these results suggest that mesoderm development in lophophorates uses a similar set of transcription factors, but their hierarchical deployment differs, suggesting profound differences in their mesodermal patterning and mesoderm regionalization. Data from bryozoans, the potential sister group of phoronids, suggest similar spatial differences in the mesodermal patterning, such as the posterior expression of *foxc* (Vellutini et al., 2017). Moreover, comparative studies of the expression profiles of endomesoderm and ectomesoderm in lophotrochozoans have revealed some intriguing differences, such as the confinement of *twist* expression in the ectomesoderm of the mollusks *Crepidula fornicata* (Perry et al., 2015), *Patella vulgata* (Nederbragt et al., 2002) and the annelid *Capitella teleta* (Dill et al., 2007), but not in the annelids *Alitta* *virens* and *Platynereis dumerilii*, in which *twist* is expressed in both sources of mesoderm (Kozin et al., 2016; Pfeifer et al., 2014; Steinmetz, 2006).

It thus becomes evident that the spatial and temporal differences in lophophorate mesoderm development are observed in more spiralian taxa, which suggests a possible diversification of mesodermal developmental programs and their underlying gene regulatory networks (GRNs). Different circuitries of GRNs orchestrating the formation of homologous mesodermal derivatives have been described in some animals and support the idea that the evolution of GRNs is mainly based on the developmental regulatory demands of each network (Andrikou and Arnone, 2015; Erkenbrack, 2016; Erkenbrack et al., 2018; Hinman and Davidson, 2007). Therefore, alterations in GRN circuitries do not necessarily reflect convergent evolution of the resulting tissues (Davidson and Erwin, 2006; Peter, 2020), but can also be a product of developmental system drift (True and Haag, 2001).

### FGF signaling upstream of different mesodermal populations

FGF signaling is required for the formation of all or most mesoderm, e.g. in hemichordates (Fan et al., 2018; Green et al., 2013) and tunicates (Davidson et al., 2006; Imai et al., 2002; Kim and Nishida, 2001; Yasuo and Hudson, 2007), or a subset of mesoderm, e.g. in vertebrates (Amaya et al., 1993; Draper et al., 2003; Fletcher et al., 2006; Fletcher and Harland, 2008; Yamaguchi et al., 1994), cephalochordates (Bertrand et al., 2011), sea urchins (Andrikou et al., 2015) and nematodes (Photos et al., 2006) (Fig. S12). According to our results, this is similar to lophophorates, in which FGF acts on different levels of mesoderm development. Although in *T. transversa* FGF is only involved in mesoderm migration and differentiation, *N. anomala* uses FGF to form mesodermal subsets and, in *Ph. harmeri*, FGF is upstream of mesoderm induction. It remains unclear why mesodermal subpopulations differ in their promoting requirements and deploy different signals. The acquisition of different signaling pathways, with distinct spatiotemporal expression dynamics and inductive properties, can act as a relay mechanism of the initial signal but can also exhibit diverse functions. An example is the recruitment of Nodal in vertebrate development – although it interacts synergistically with FGF in promoting mesoderm, it also acts differentially in the induction of mesodermal populations (Kimelman, 2006; Mathieu et al., 2004).

### Implications of mesoderm development in gastrulation and axial elongation

Besides having a role in mesoderm development, FGF signaling has conserved functions in neural development and morphogenetic movements of gastrulation in an array of investigated organisms (Fig. S12). In deuterostomes, FGF is involved both in gastrulation (Amaya et al., 1991; Bertrand et al., 2011; Röttinger et al., 2008) and neural induction (Bertrand et al., 2003; De Robertis and Kuroda, 2004; Garner et al., 2016). Also, in the two well-studied ecdysozoans *D. melanogaster* and *C. elegans*, FGF signaling is upstream of axon guidance (Bülow et al., 2004; García-Alonso et al., 2000) and cell migration during gastrulation (in *D. melanogaster*) (Leptin and Affolter, 2004). In the remaining protostomes, data are limited to gastropods and Platyhelminthes, in which FGF signaling is involved in neural development (Cebrià et al., 2002; Pollak et al., 2014). Most likely, the involvement of FGF in these developmental processes was already present before the cnidarian-bilaterian split, as witnessed in sea anemones, in which FGF appears to act upon gastrulation (Matus et al., 2007) and neural development (Matus et al., 2007), and is upstream of apical organ formation (Rentzsch et al., 2008). Our study revealed similar roles of FGF signaling in the investigated lophophorate species. In particular, all three species exhibited defects in their apical organ/neuropile formation, as well as loss of a number of differentiated neurons (e.g. serotonergic neurons in Fig. 6Bii,Biii). Moreover, they all showed impaired gastrulation to some degree. Impaired gastrulation can be either correlated with a failure in mesoderm formation or an indirect effect. For example, in *T. transversa*, in which mesoderm is formed independently of FGF signaling, most likely the role of FGF is only morphogenetic, in orchestrating cell movements during gastrulation. However, in the other two species, in which FGF is somehow involved in mesoderm formation, it is still unclear whether the observed failure in gastrulation movements after FGF inhibition is associated with the lack of mesoderm formation or is an independent event. Another outcome of this study concerns the apparent relationship witnessed between mesoderm development and posterior axis elongation in *T. transversa*. The expression of *fgf8/17/18* mRNA in the growing posterior tip of the embryo in relation to the mesodermal expression of *fgfr* (Fig. S4) suggests that FGF8/17/18 might progressively coordinate the posterior axial elongation of the embryo and mesoderm differentiation, perhaps similarly to what has been described in vertebrates (Dubrulle and Pourquie, 2004).

To summarize, the data provide support for conserved involvement of FGF signaling in gastrulation movements and axial elongation, with the phenotypic severity varying, depending on the developmental mode of mesoderm formation of the investigated species.

### The recurrent use of FGF signaling in mesoderm formation

The role of FGF signaling in mesoderm induction was thought to be restricted to members of deuterostomes. After investigating three species of lophophorates, we are able to show that the mesoderm-inducing ability of this pathway extends to the lineage of protostomes.

However, signaling pathways are often deployed as upstream ‘plug-in’ devices and can be co-opted and exchanged to serve different developmental processes within and among species (Davidson and Erwin, 2006). To determine whether the involvement of FGF signaling in mesoderm formation was already present in the last common ancestor of Bilateria, or whether it was independently co-opted in the lineage of lophophorates, functional studies from more spiralian taxa are required. So far, the only available data in favor of a putative conserved role of FGF in mesoderm induction come from studies in mollusks, in which MAPK – often downstream of the FGF signaling cascade – is upstream of endomesoderm specification (Koop et al., 2007; Kozin et al., 2013; Lambert, 2008; Lambert and Nagy, 2001, 2003), and in bryozoans, as suggested from the activation of the MAPK pathway in the endomesodermal precursor cell (3D blastomere) (Vellutini et al., 2017).

## MATERIALS AND METHODS

### Animal systems

Gravid adult specimens were collected in Bodega Bay, CA, USA (*Ph. harmeri* Pixell, 1912), in Friday Harbor Laboratories, USA. (*T. transversa* Sowerby, 1846), in Espeland Marine Biological Station, Norway (*N. anomala* Müller, 1776) and spawned as previously described (Freeman, 1993, 2000; Rattenbury, 1954). The embryos were kept in clean seawater and collected at various stages of development.

### Gene cloning and orthology assignment

Putative orthologous sequences of genes of interest were identified by tBLASTx search against the transcriptomes of *T. transversa*, *N. anomala* and *Ph. harmeri*. Gene orthology of genes of interest identified by tBLASTx was tested by reciprocal BLAST against NCBI Genbank and followed by phylogenetic analyses. Amino acid alignments were made using MUSCLE. IQ-tree (version 2.0.5) was used to conduct a maximum likelihood phylogenetic analysis. Fragments of the genes of interest were amplified from cDNA of *T. transversa*, *N. anomala* and *Ph. harmeri* by PCR using gene-specific primers. PCR products were purified and cloned into a pGEM-T Easy vector (Promega, A1360) according to the manufacturer's instructions and the identity of inserts confirmed by sequencing. Primer sequences and the size of the products are provided in Table S2.

### SU5402 treatments

SU5402 (Sigma-Aldrich, SML0443) was dissolved in DMSO to a stock solution of 10 mM and then serially diluted in the concentrations of 5 µm, 10 µM and 20 μM in seawater. Higher concentrations than these were lethal to the embryos. SU5402 was added at morula, blastula and gastrula stages up to the fixation stage. A corresponding volume of DMSO was added in the control embryos. Solutions were changed every 24 h. The drug treatments and observed phenotypes are summarized in Figs S6 and S7.

### Whole-mount *in situ* hybridization

Embryos were manually collected, fixed in 4% paraformaldehyde in filtered sea water for 60 min, permeabilized in 100% methanol overnight and processed for colorimetric and double fluorescent *in situ* hybridization as previously described (Andrikou et al., 2019; Martín-Durán et al., 2017; Santagata et al., 2012). Labeled antisense RNA probes were transcribed from linearized DNA using digoxigenin-11-UTP (Roche, 11209256910) and dinitrophenol (DNP) (Mirus, 3825) according to the manufacturer's instructions.

### Whole-mount immunohistochemistry

Embryos were permeabilized in 100% methanol for 1 h, digested with Proteinase K (10 µg ml^−1^; Sigma-Aldrich) for 5 min, fixed in 4% paraformaldehyde in sea water for 30 min, washed for 3 h in phosphate buffer saline containing 0.5% Triton (PTX), washed once in phosphate buffer containing 0.1% Tween-20 (PBT) for 5 min and incubated in 4% sheep serum in PBT for 30 min. The animals were then incubated with commercially available primary antibodies overnight at 4°C, washed three times in PBT, and followed by incubation in 4% sheep serum in PBT for 30 min. Primary antibodies used were: anti-acetylated tubulin mouse monoclonal antibody (1:250, Sigma-Aldrich, T6793); anti-actin mouse monoclonal antibody (1:400, Seven Hills Bioreagents, LMAB-C4); anti-serotonin rabbit monoclonal antibody (1:1000, Sigma-Aldrich, S5545); anti-FMFRamide rabbit monoclonal antibody (1:200, Immunostar, 20091). Specimens were then incubated with secondary anti-rabbit and anti-mouse antibodies Alexa Fluor (1:1000, Life Technologies, A21245/A21424) overnight at 4°C followed by three washes in PBT. Nuclei were stained with DAPI (Invitrogen, D1306) and F-actin was visualized with BODIPY FL Phallacidin (Life Technologies, N354).

### Documentation

Colorimetric whole-mount *in situ* hybridization specimens were imaged using a Zeiss AxioCam HRc mounted on a Zeiss Axioscope A1 equipped with Nomarski optics and processed through Photoshop CS6 (Adobe). Fluorescent-labeled specimens were analyzed with an SP5 confocal laser microscope (Leica, Germany) and processed by the ImageJ software version 2.0.0-rc-42/1.50d (Wayne Rasband, National Institutes of Health). Figure plates were arranged with Illustrator CS6 (Adobe).

## Supplementary Material

Supplementary information

Reviewer comments
