# Supplementary material for: FGF signaling acts on different levels of mesoderm development within Spiralia
Source: Development. 2021 May 17;148(10):dev196089. doi: 10.1242/dev.196089 (PMC8180254; doi:10.1242/dev.196089)
Supplement: Supplementary information [file develop-148-196089-s1.pdf]

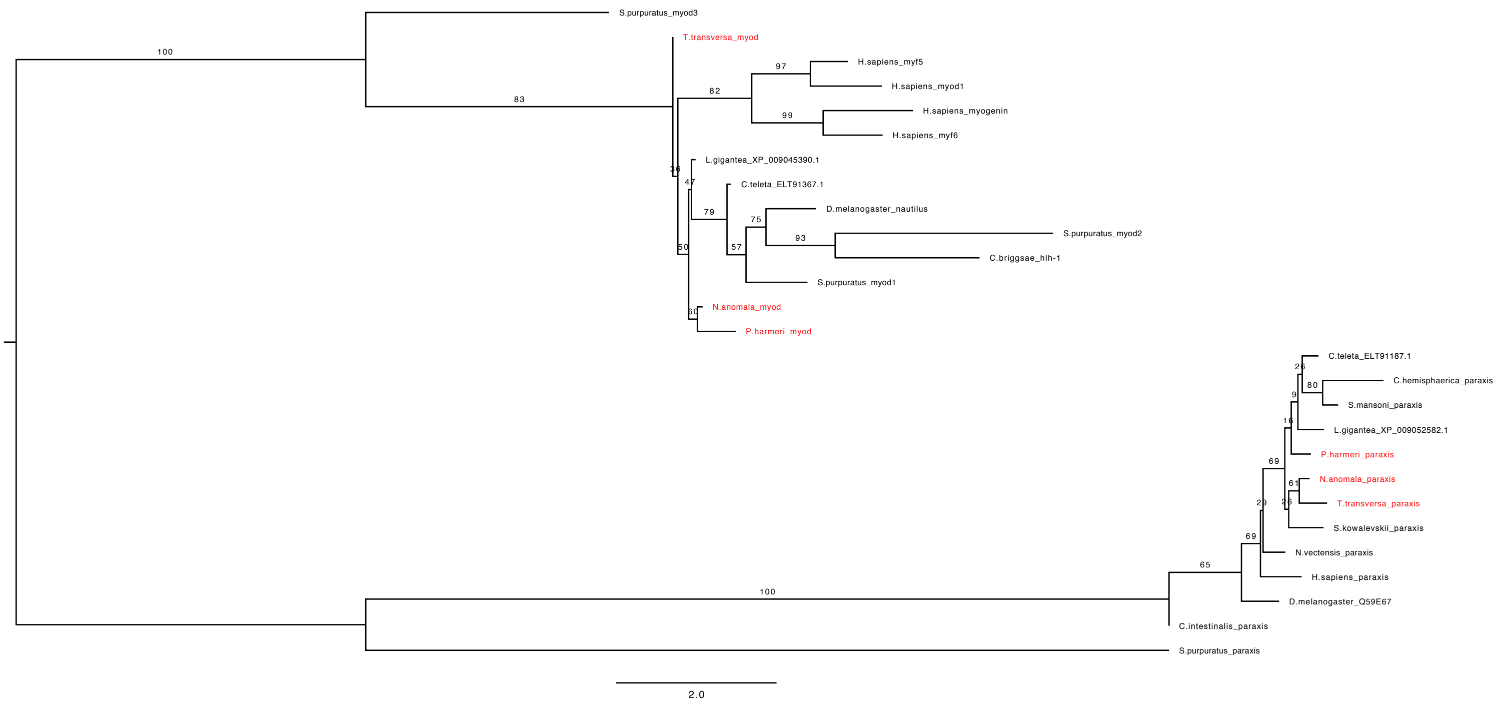

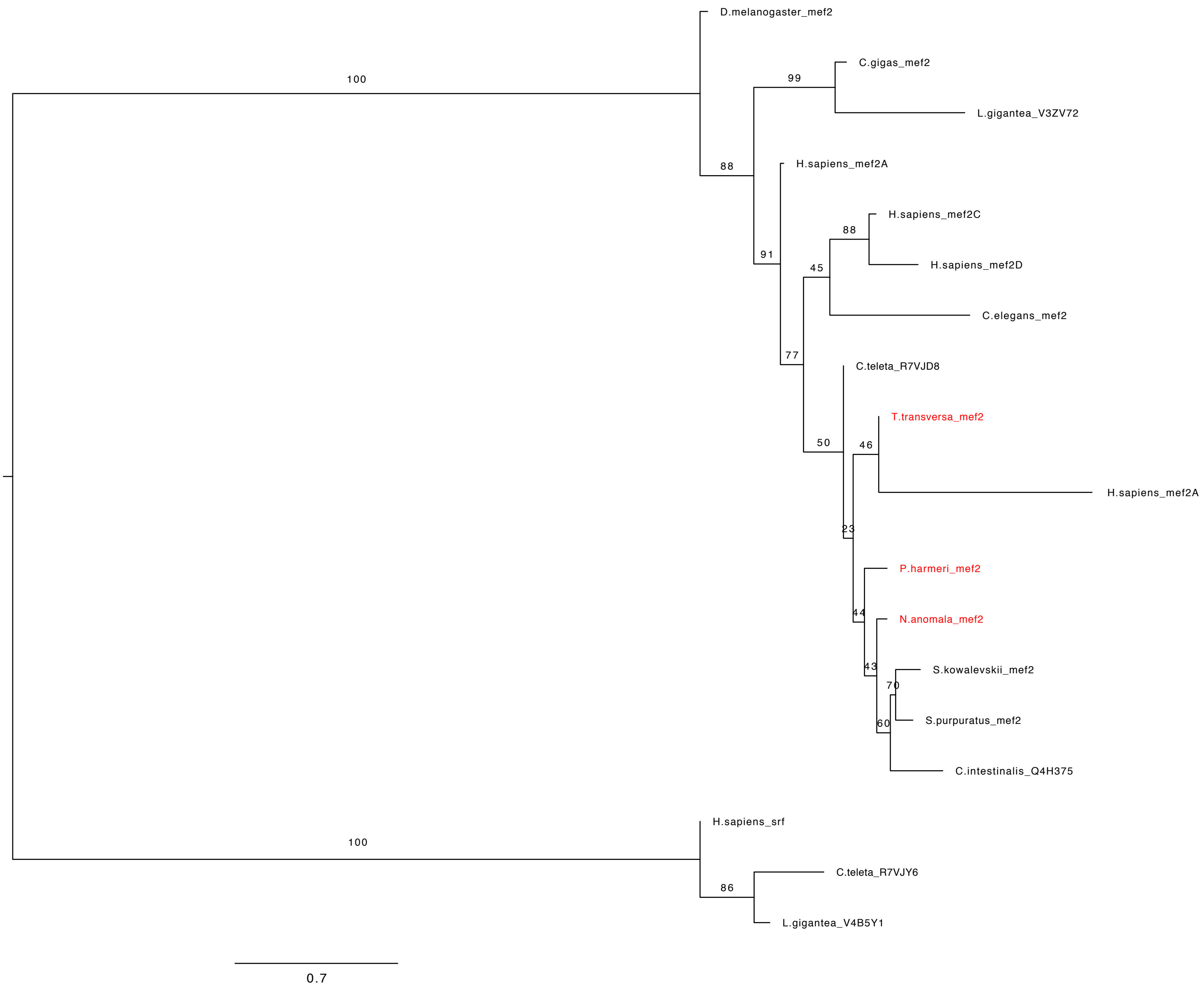

Development • Supplementary information

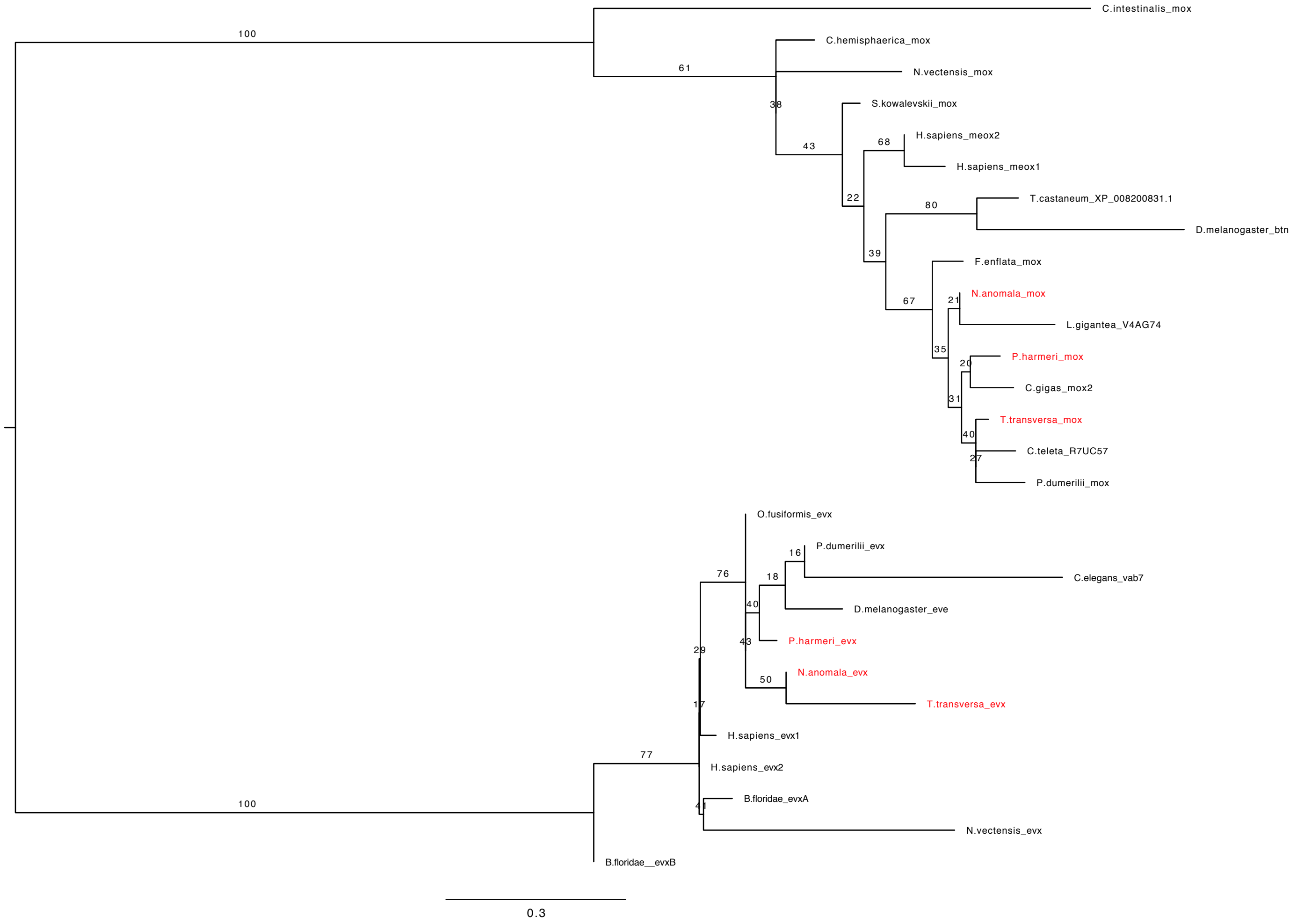
C.intestinalis_mox
C.hemisphaerica_mox
N.vectensis_mox
S.kowalevskii_mox
H.sapiens_meox2
H.sapiens_meox1
T.castaneum_XP_008200831.1
D.melanogaster_btn
F.enflata_mox
N.anomala_mox
L.gigantea_V4AG74
P.harmeri_mox
C.gigas_mox2
T.transversa_mox
C.teleta_R7UC57
P.dumerilii_mox
O.fusiformis_evx
P.dumerilii_evx
C.elegans_vab7
D.melanogaster_eve
P.harmeri_evx
N.anomala_evx
T.transversa_evx
H.sapiens_evx1
H.sapiens_evx2
B.floridae_evxA
N.vectensis_evx
B.floridae__evxB
0.3

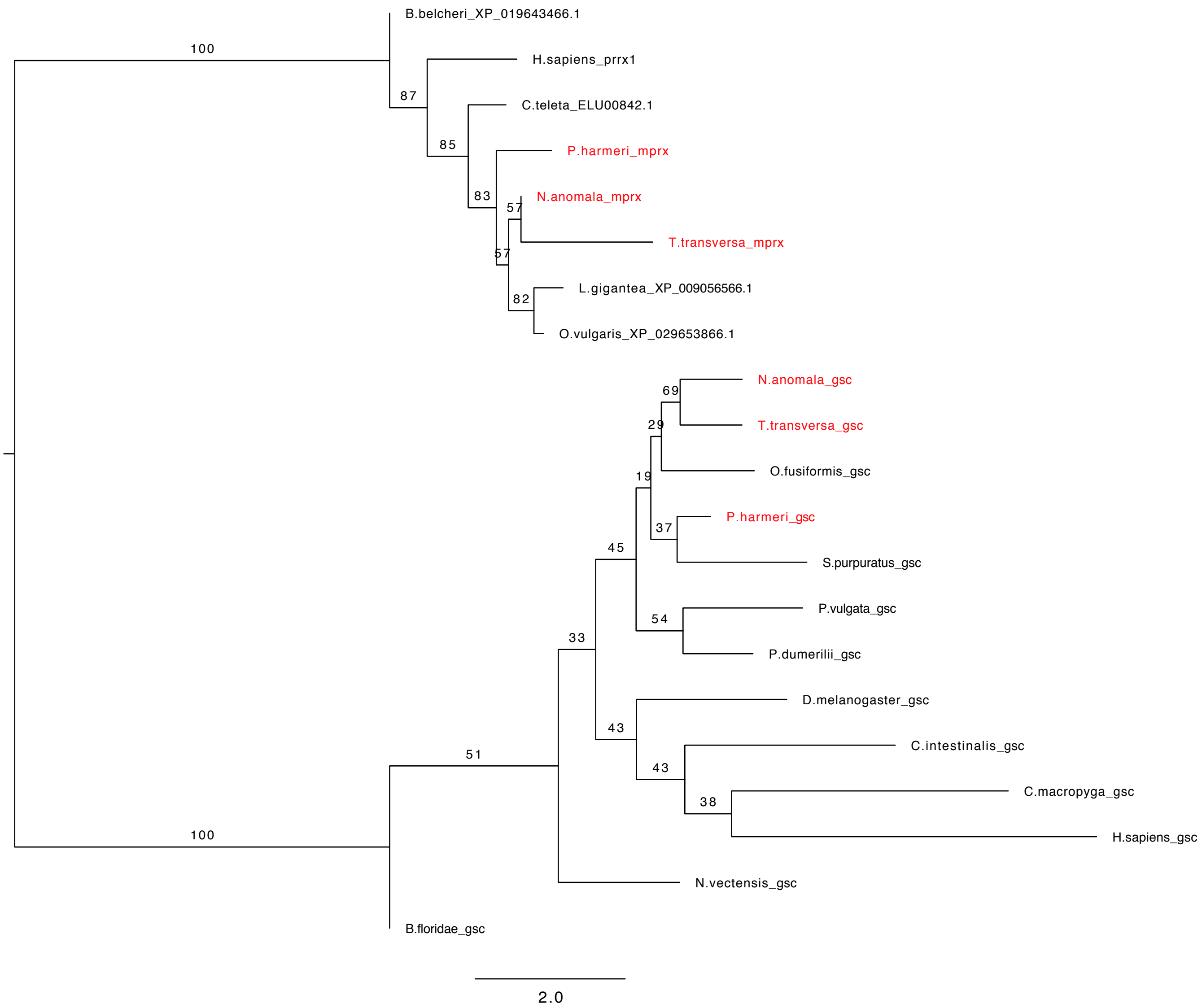
B.belcheri_XP_019643466.1
H.sapiens_prrx1
C.teleta_ELU00842.1
P.harmeri_mprx
N.anomala_mprx
T.transversa_mprx
L.gigantea_XP_009056566.1
O.vulgaris_XP_029653866.1
N.anomala_gsc
T.transversa_gsc
O.fusiformis_gsc
P.harmeri_gsc
S.purpuratus_gsc
P.vulgata_gsc
P.dumerilii_gsc
D.melanogaster_gsc
C.intestinalis_gsc
C.macropyga_gsc
H.sapiens_gsc
N.vectensis_gsc
B.floridae_gsc
2.0

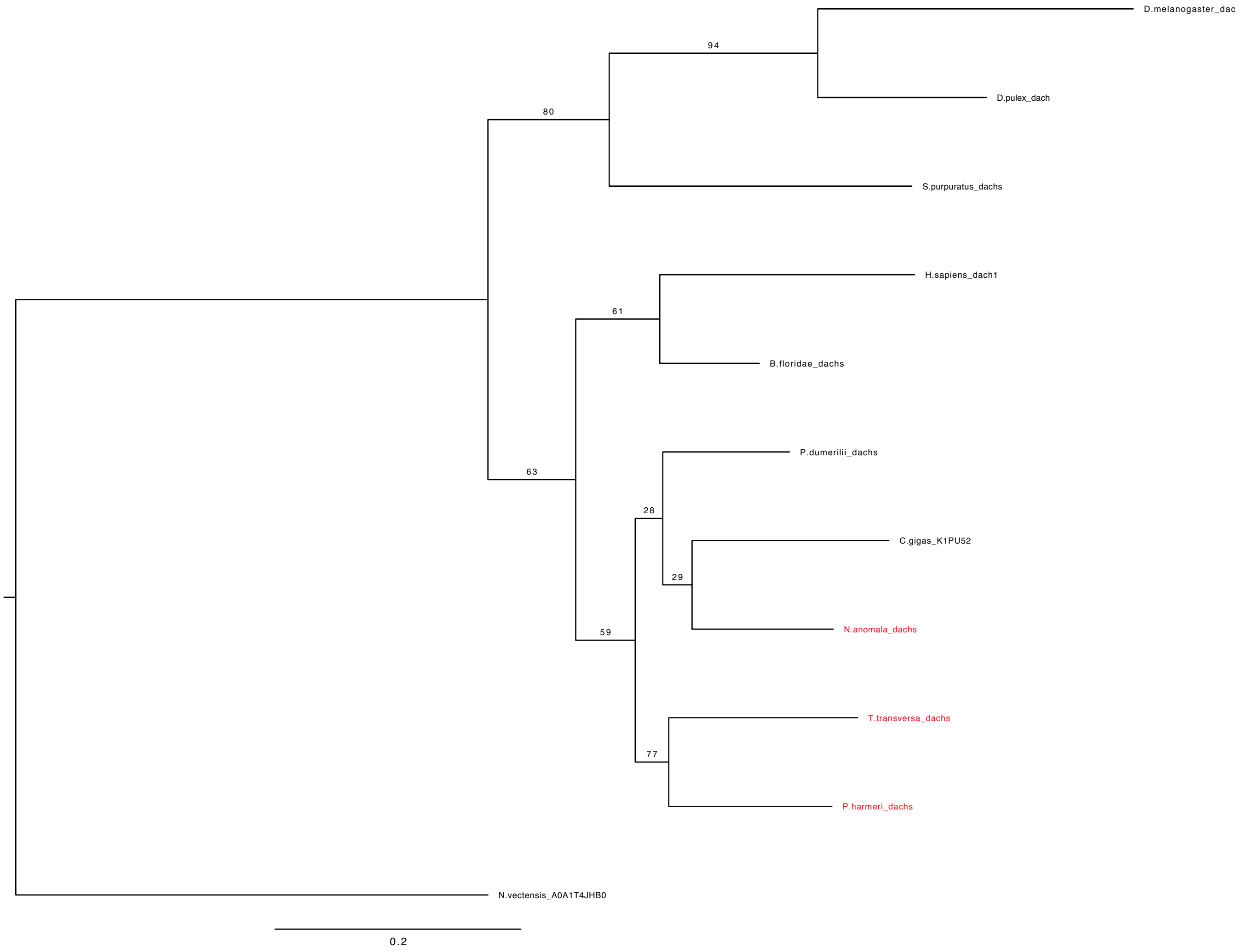

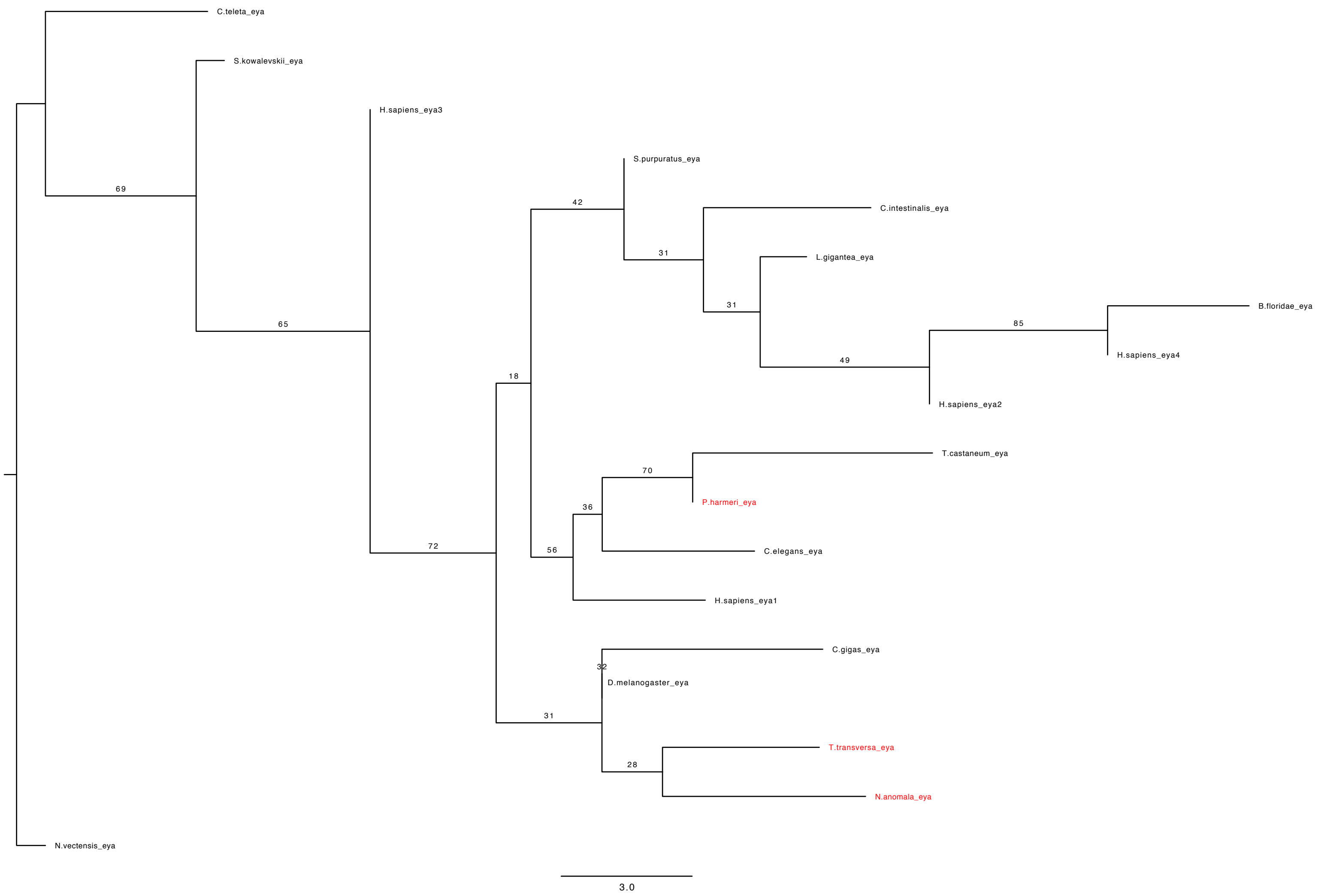

Development • Supplementary information

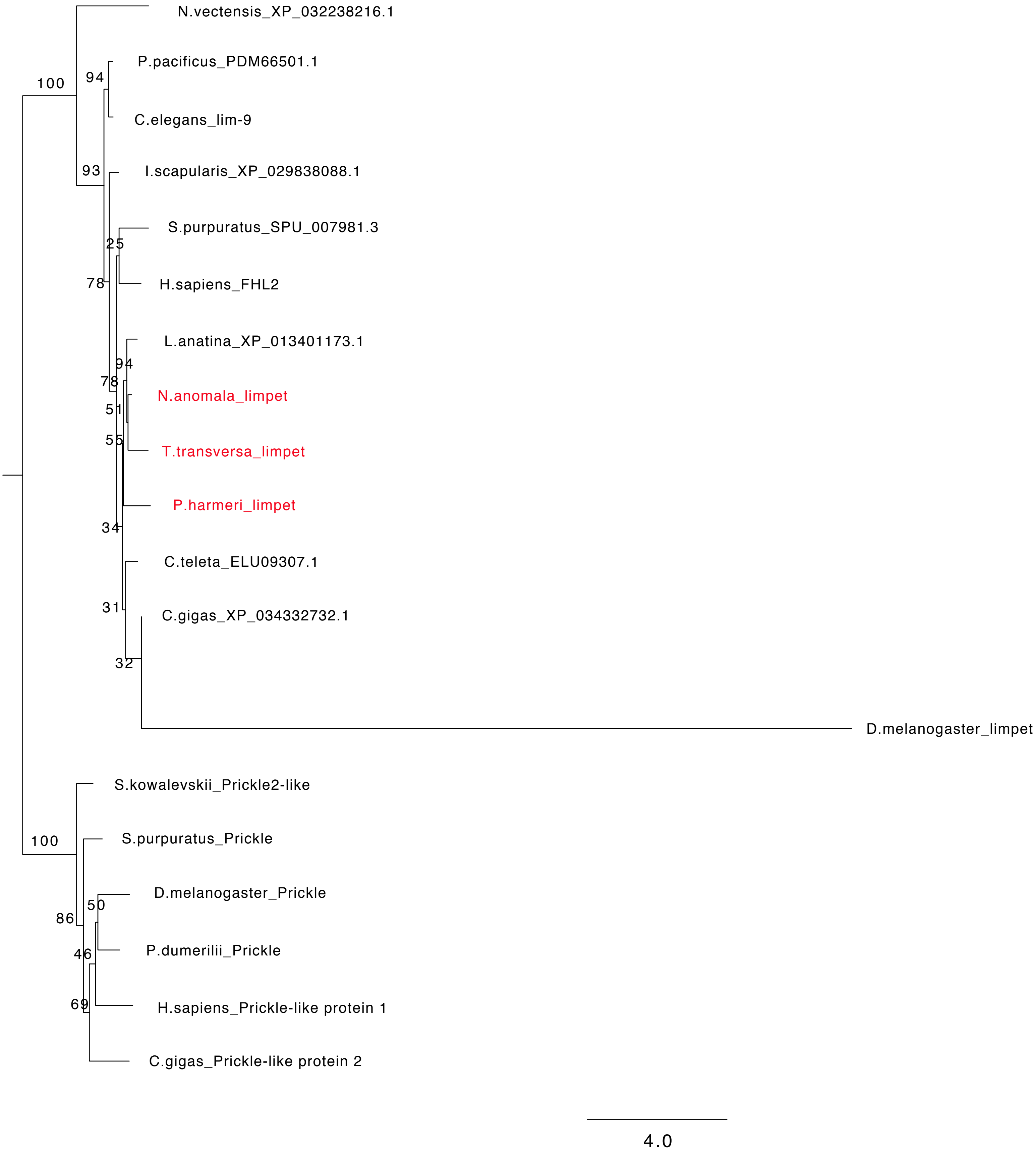

Development • Supplementary information

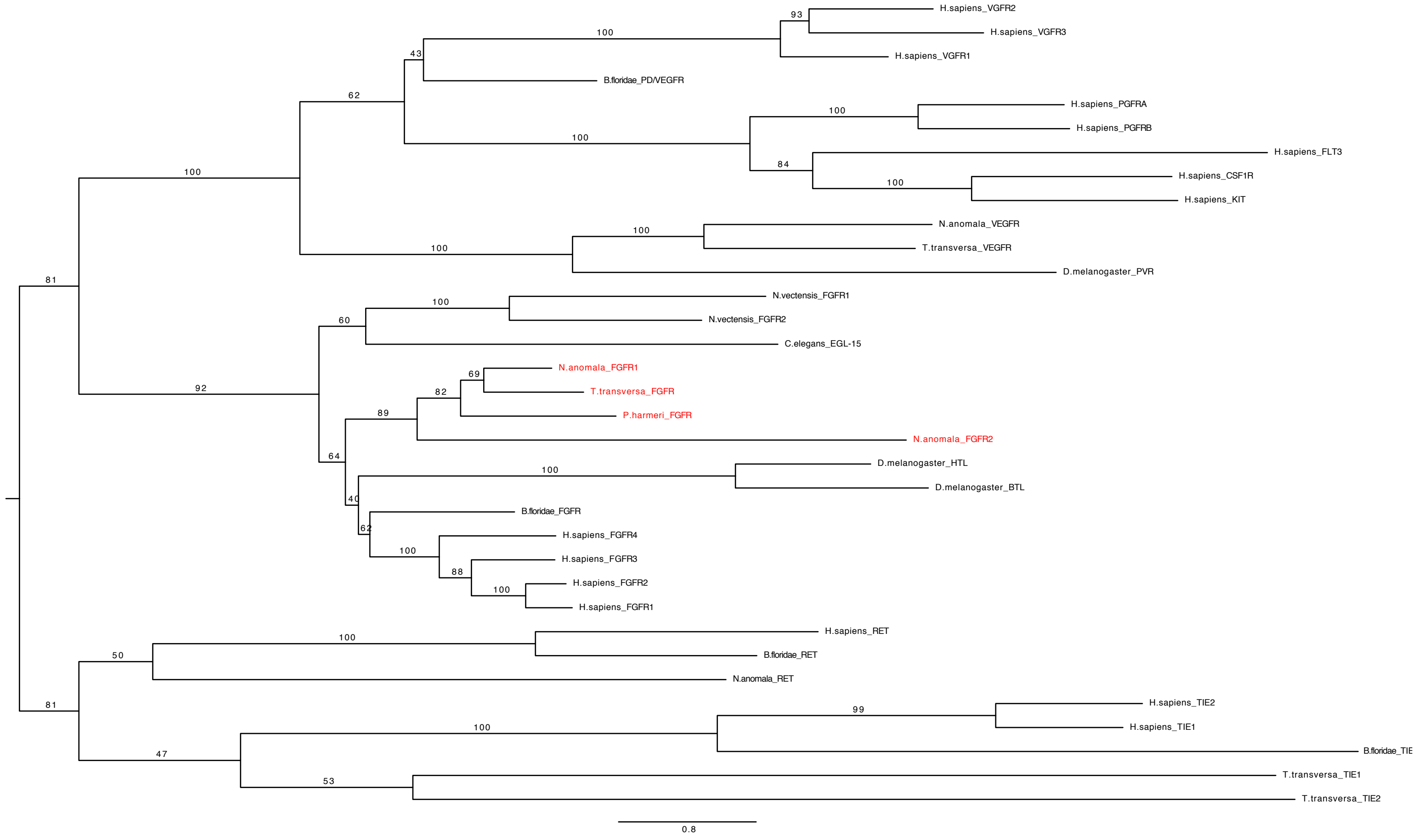
H.sapiens_VGFR2
H.sapiens_VGFR3
H.sapiens_VGFR1
B.floridae_PD/VEGFR
H.sapiens_PGFRA
H.sapiens_PGFRB
H.sapiens_FLT3
H.sapiens_CSF1R
H.sapiens_KIT
N.anomala_VEGFR
T.transversa_VEGFR
D.melanogaster_PVR
N.vectensis_FGFR1
N.vectensis_FGFR2
C.elegans_EGL-15
N.anomala_FGFR1
T.transversa_FGFR
P.harmeri_FGFR
N.anomala_FGFR2
D.melanogaster_HTL
D.melanogaster_BTL
B.floridae_FGFR
H.sapiens_FGFR4
H.sapiens_FGFR3
H.sapiens_FGFR2
H.sapiens_FGFR1
H.sapiens_RET
B.floridae_RET
N.anomala_RET
H.sapiens_TIE2
H.sapiens_TIE1
B.floridae_TIE
T.transversa_TIE1
T.transversa_TIE2
0.8

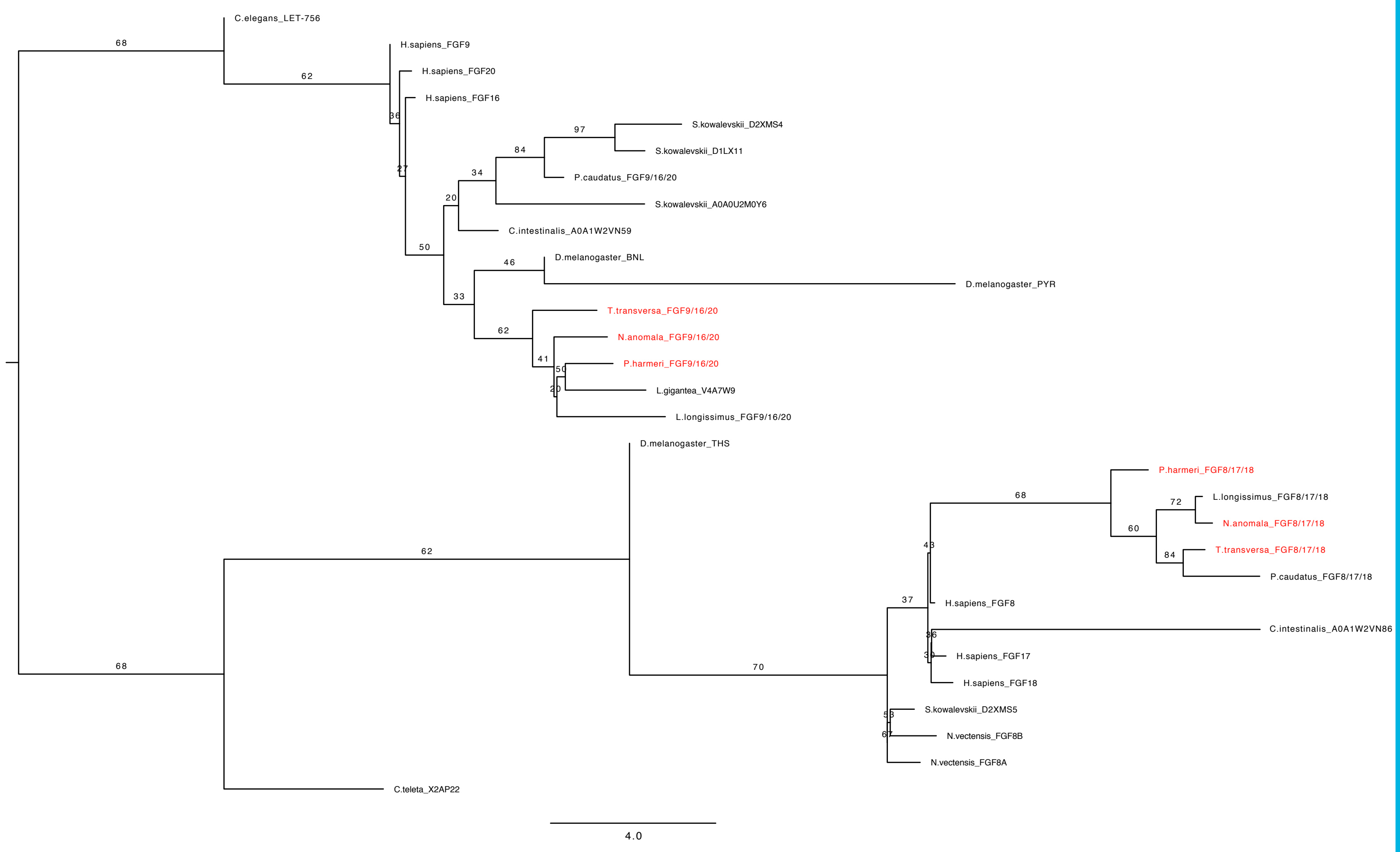
C.elegans_LET-756
H.sapiens_FGF9
H.sapiens_FGF20
H.sapiens_FGF16
S.kowalevskii_D2XMS4
S.kowalevskii_D1LX11
P.caudatus_FGF9/16/20
S.kowalevskii_A0A0U2M0Y6
C.intestinalis_A0A1W2VN59
D.melanogaster_BNL
D.melanogaster_PYR
T.transversa_FGF9/16/20
N.anomala_FGF9/16/20
P.harmeri_FGF9/16/20
L.gigantea_V4A7W9
L.longissimus_FGF9/16/20
D.melanogaster_THS
P.harmeri_FGF8/17/18
L.longissimus_FGF8/17/18
N.anomala_FGF8/17/18
T.transversa_FGF8/17/18
P.caudatus_FGF8/17/18
H.sapiens_FGF8
C.intestinalis_A0A1W2VN86
H.sapiens_FGF17
H.sapiens_FGF18
S.kowalevskii_D2XMS5
N.vectensis_FGF8B
N.vectensis_FGF8A
C.teleta_X2AP22
4.0

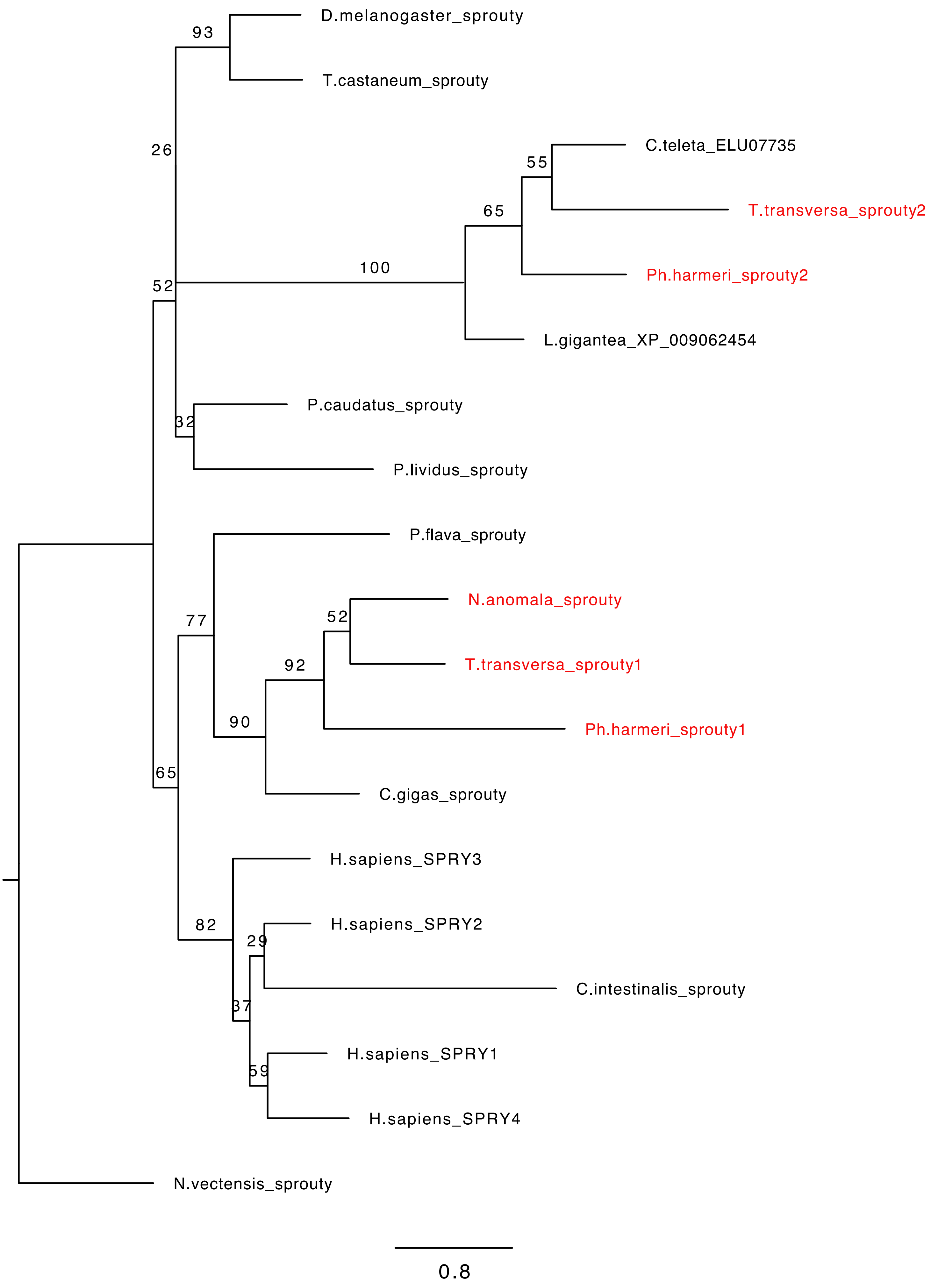

Fig. S1. **Orthology analysis.** Putative orthologous sequences were identified by tBLASTx search of the transcriptome of *T. transversa, N. anomala* and *Ph. harmeri*. Maximum likelihood phylogenetic analysis is supporting orthology. Names of genes or proteins, if available, follow the name of organism(s). *T. transversa, N. anomala* and *Ph. harmeri* sequences are highlighted in red.

Development • Supplementary information

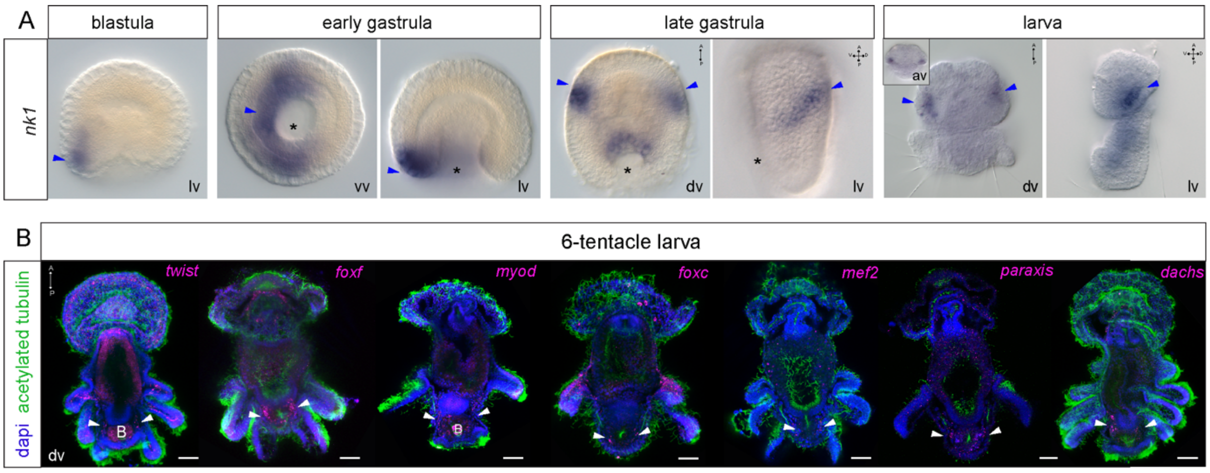

Fig. S2. **Additional gene expression patterns**. A) WMISH of *nk1* in blastulae, early gastrulae, late gastrulae and larvae of *N. anomala*. The inset indicates an anterior view of the embryo. Blue arrowheads indicate ectodermal staining. The position of the blastopore is indicated by an asterisk. B) Fluorescent WMISH of *twist, foxf, myod, foxc, mef2, paraxis* and *dachs* in the 6-tentacle larva stage of *Ph. harmeri*. Every fluorescent image is a Z-projection of merged confocal stacks. Gene expression is in magenta, cilia are labeled green with anti-acetylated tubulin antibody and nuclei are stained blue with DAPI. Posterior mesoderm is indicated with white arrowheads. Anterior to the top. dv, dorsoventral view; lv, lateral view; vv, vegetal view. B, background staining. Scale bar: 20 µm.

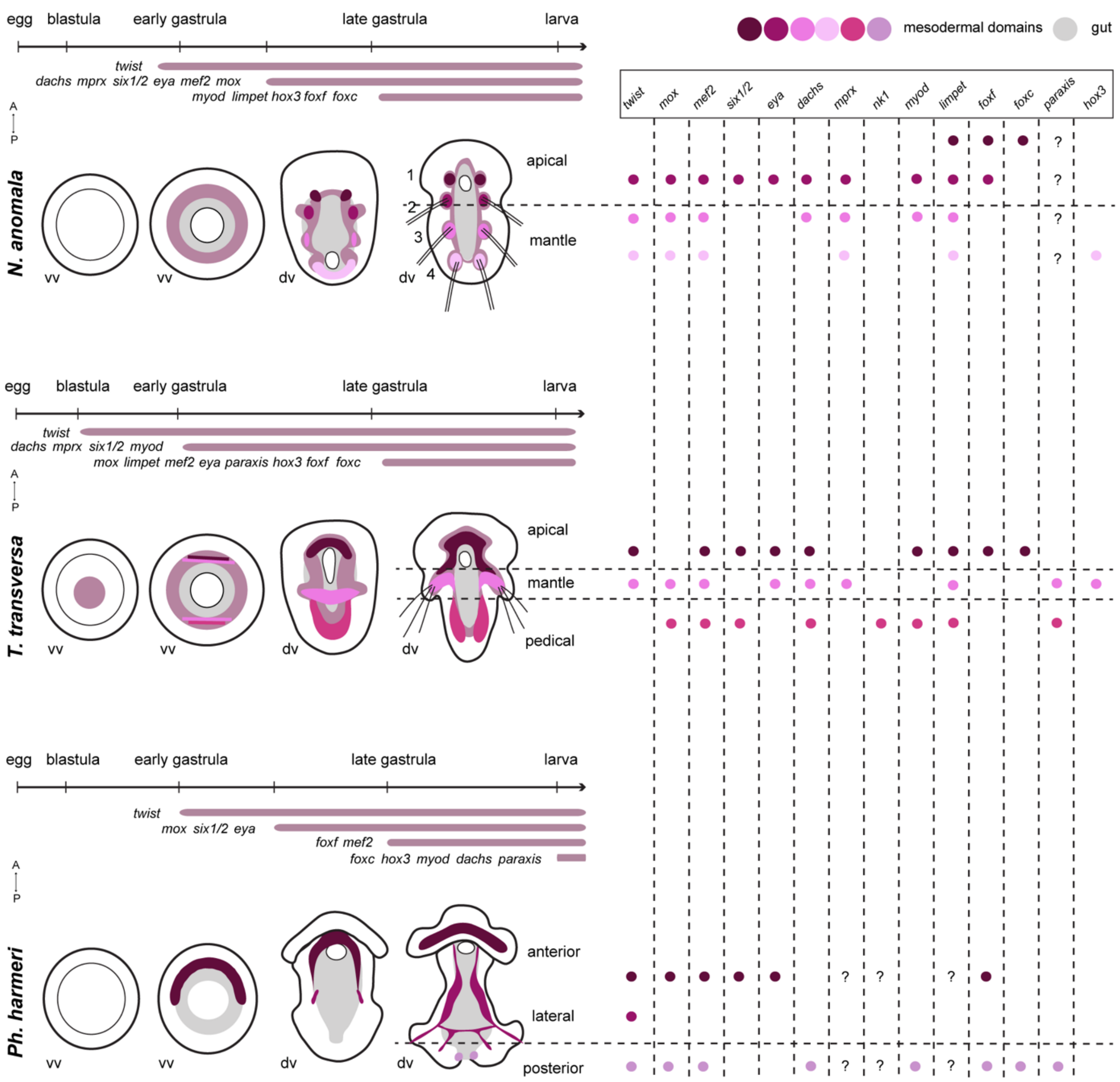

Fig. S3. **Comparison of mesodermal gene expression patterns in representative developmental stages of *N. anomala, T. transversa* and *Ph. harmeri*.** Schematic representation of the temporal and spatial expression patterns of *twist, mox, mef2, six1/2, eya, dachs, mprx, nk1, myod, limpet, foxf, foxc* and *hox3* in blastula, gastrula and larva stages of *N. anomala, T. transversa* and *Ph. harmeri*. *Nk1* and *hox3* do not exhibit a mesodermal expression in *N. anomala* and *Ph. harmeri,* respectively. Question marks indicate that the expression of these genes is not yet known. Drawings are not up to scale. Anterior to the top. dv, dorsoventral view; vv, vegetal view.

Development • Supplementary information

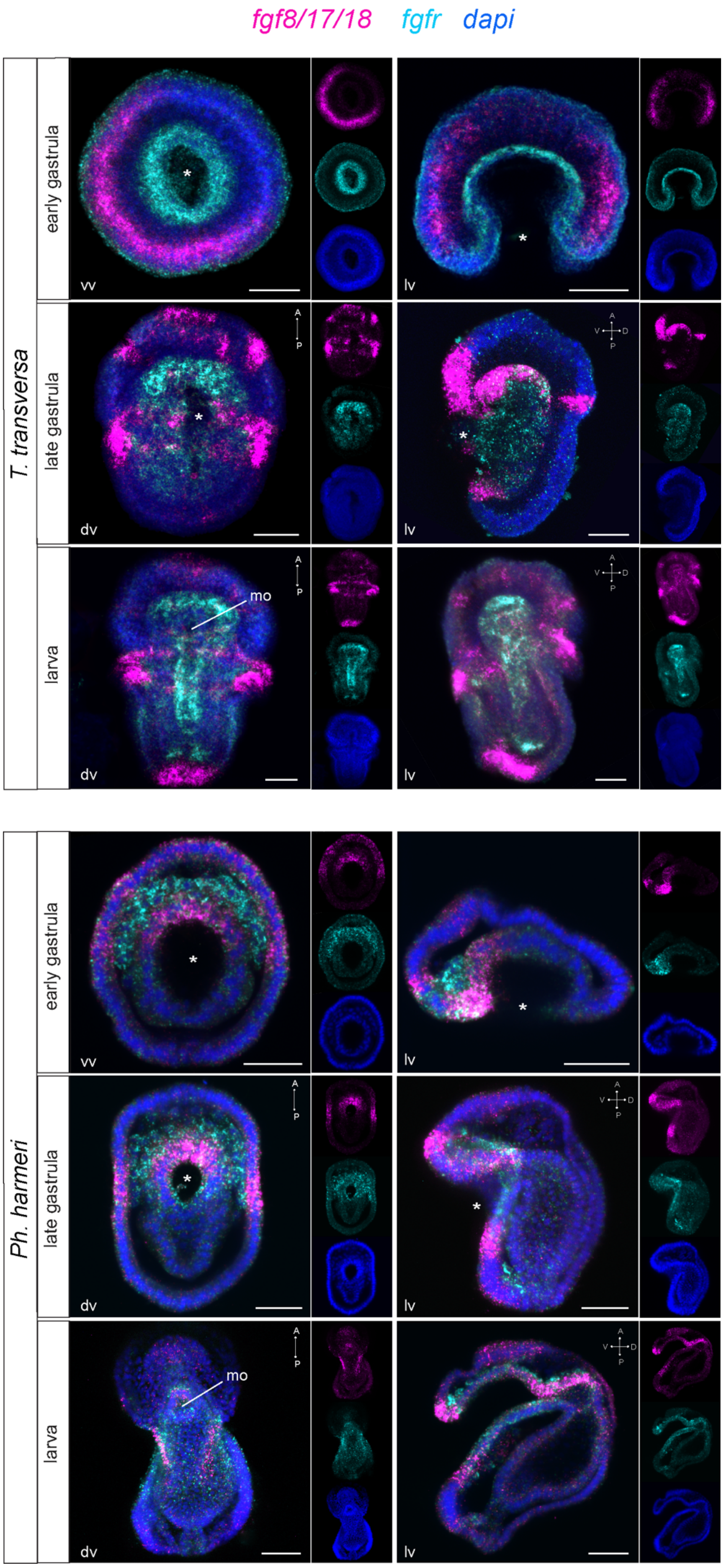

Fig. S4. **Co-expression analysis of *fgfr* (cyan) and *fgf8/17/18* (magenta) by double fluorescent WMISH in *T. transversa* and *Ph. harmeri*.** Every fluorescent image is a Z-projection of merged confocal stacks and nuclei are stained with DAPI. The position of the blastopore is indicated by an asterisk. Anterior to the top. dv, dorsoventral view; lv, lateral view; mo, mouth; vv, vegetal view. Scale bar: 20 μm.

Development • Supplementary information

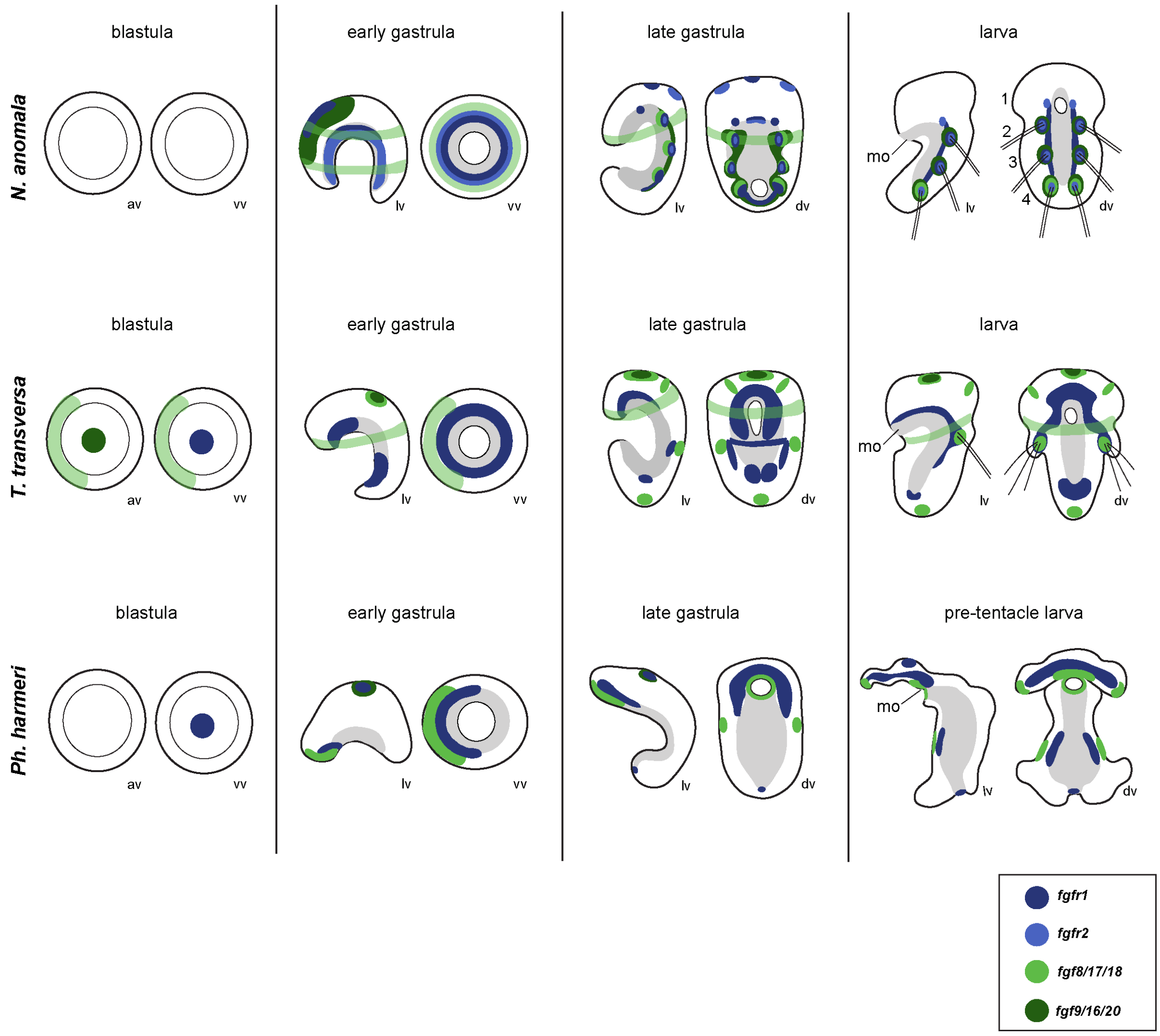

Fig. S5. **Comparison of gene expression patterns of the FGF signaling components in representative developmental stages of *N. anomala, T. transversa* and *Ph. harmeri*.** Schematic representation of the expression patterns of *fgfr1, fgfr2, fgf8/17/18* and *fgf9/16/20* in blastula, gastrula and larva stages of *N. anomala, T. transversa* and *Ph. harmeri*. Drawings are not up to scale. Anterior to the top. av, animal view; dv, dorsoventral view; lv, lateral view; mo, mouth; vv, vegetal view.

Development • Supplementary information

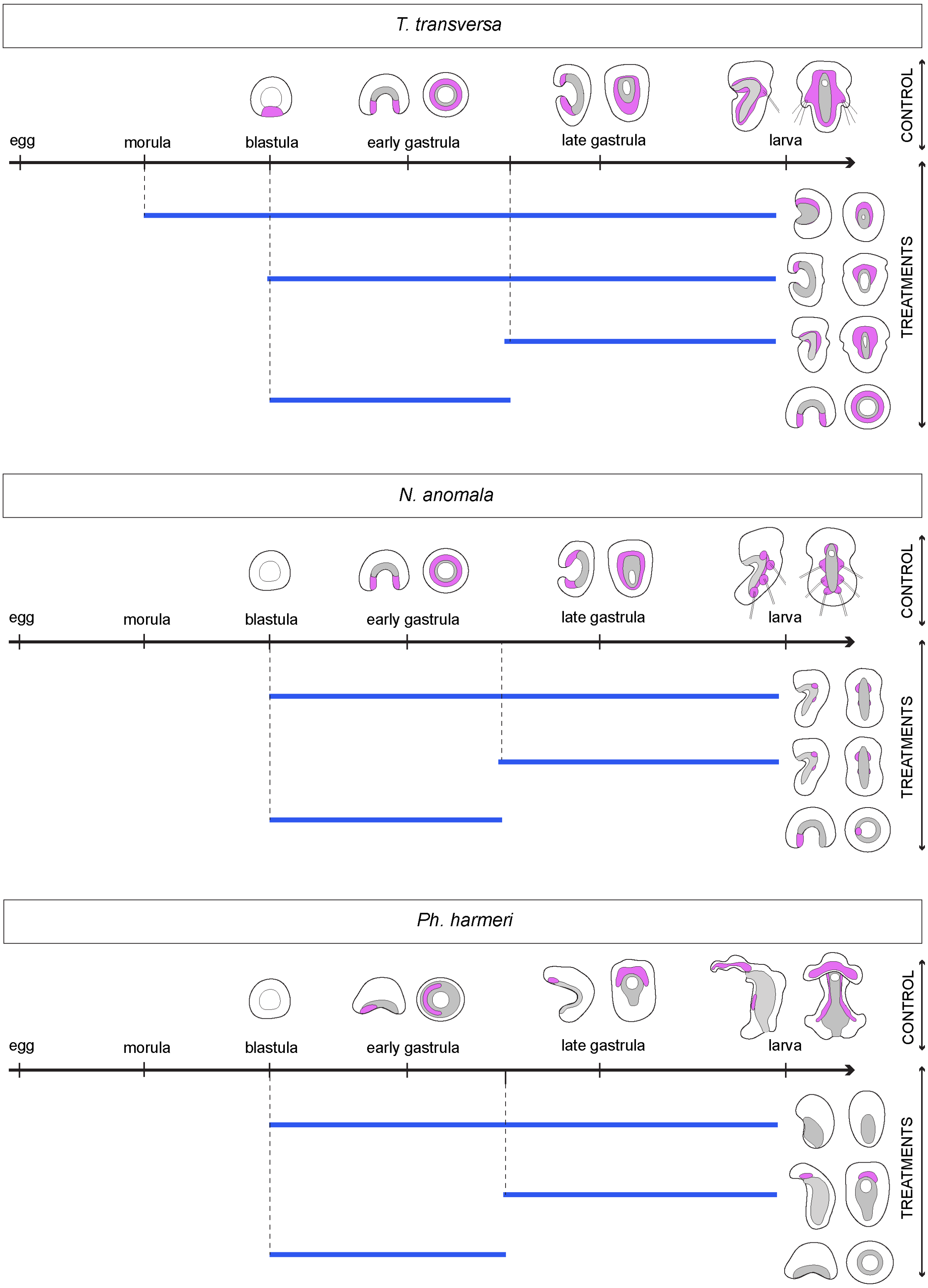

Fig. S6. **Diagrams of 20 μM SU5402 treatments and schemes of observed phenotypes in *T. transversa, N. anomala* and *Ph. harmeri*.** Embryos were treated with 20 μM SU5402 from morula (only in *T. transversa*), blastula and gastrula stages and fixed at larva stage. Embryos treated from blastula stage were also fixed at gastrula stage. Anterior to the top. Drawings are not up to scale.

Development • Supplementary information

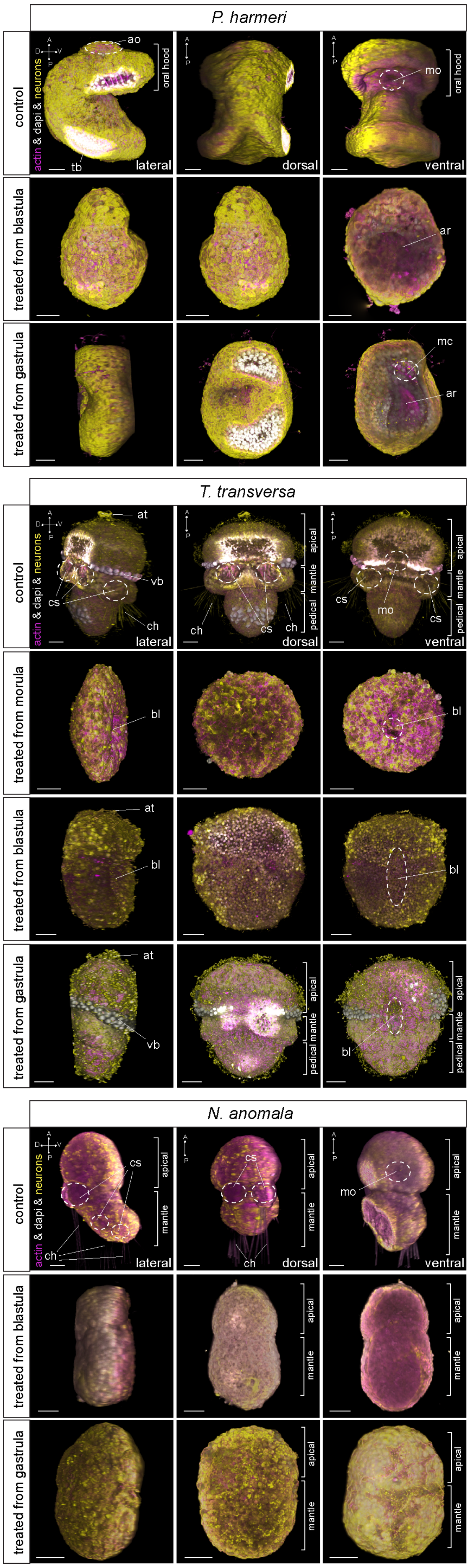

apical organ: absent
muscles: absent
size: smaller
lobes: absent
tentacular bulbs: absent
axial elongation: impaired
A/P axis: impaired
gastrulation: partial

apical organ: absent
muscles: present but reduced
size: smaller
lobes: absent
tentacular bulbs: absent
axial elongation: impaired
A/P axis: impaired
gastrulation: normal, with the blastopore shifted anteriorly

apical tuft: absent
neuropile: absent
muscles and coelomic sacs: absent
size: smaller
lobes: absent
vesicular bodies: absent
chaetae bundles: absent
axial elongation: impaired
A/P axis: normal
gastrulation: no

apical tuft: reduced
neuropile: absent
muscles and coelomic sacs: malformed
size: smaller
lobes: absent
vesicular bodies: absent
chaetae bundles: absent
axial elongation: impaired
A/P axis: normal
gastrulation: partial, arrested with open blastopore

apical tuft: reduced
neuropile: absent
muscles and coelomic sacs: malformed
size: smaller
lobes: smaller but evident, especialy the anterior one
vesicular bodies: formed
chaetae bundles: absent
axial elongation: impaired
A/P axis: normal
gastrulation: partial, arrested with open blastopore, blastopore shifted anteriorly

neuropile and neurons: reduced
muscles and coelomic sacs: malformed
size: smaller
lobes: smaller but evident
chaetae bundles: absent
axial elongation: normal
A/P axis: impaired
gastrulation: gut elongated but no mouth formed

neuropile and neurons: reduced
muscles and coelomic sacs: malformed
size: smaller
lobes: smaller but evident
chaetae bundles: absent
axial elongation: normal
A/P axis: impaired
gastrulation: gut elongated but no mouth formed

Fig. S7. **3D reconstruction and summary of the SU5402 treated phenotypes of *T. transversa, N. anomala* and *Ph. harmeri*.** Muscles and coelomic sacs are stained with magenta, neurons are yellow, and nuclei are grey. Every fluorescent image is a full projection of merged confocal stacks. Anterior to the top. ao, apical organ; ar, archenteron; at, apical tuft; bl, blastopore; ch, chaetae; cs, coelomic sacs; mc, muscle cells; mo, mouth; vb, vesicular bodies. Scale bar: 20 μm.

Development • Supplementary information

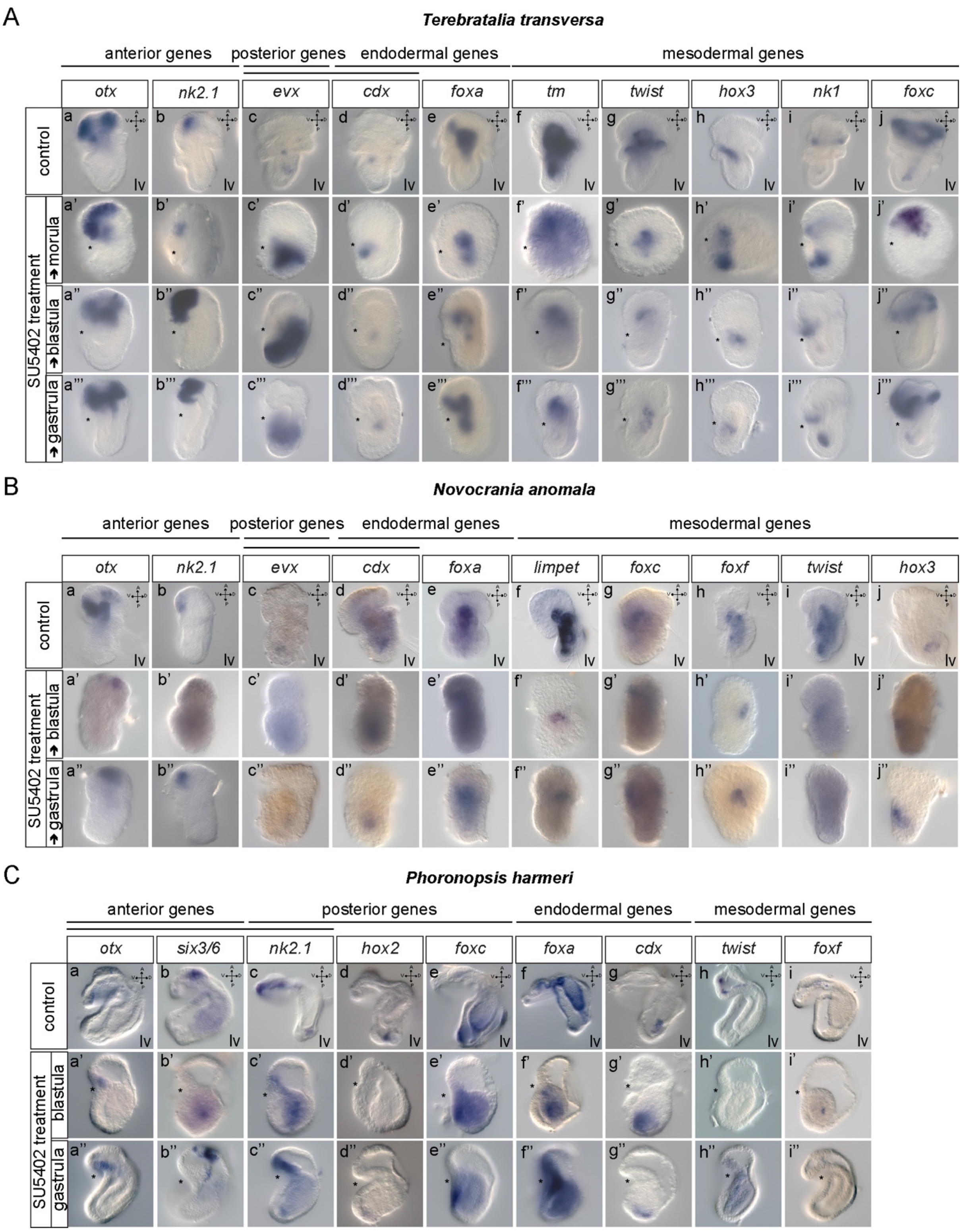

Fig. S8. **Lateral views of *T. transversa, N. anomala* and *Ph. harmeri* SU5402 treated larvae.** (A) WMISH of anterior (*otx, nk2.1, foxc, nk1*), posterior *(evx, cdx)*, endodermal *(foxa, cdx)* genes, and markers of musculature (*tropomyosin (tm))*, apical longitudinal and coelomic sac muscles (*twist),* ventral mantle lateral muscles (*hox3*) and pedicle muscles *(nk1, foxc)* in *T. transversa* morula, blastula and gastrula embryos treated with 20 μM SU5402 and fixed at the larva stage. (B) WMISH of anterior (*otx, nk2.1*), posterior genes (*evx, cdx*), endodermal *(foxa, cdx)* genes, and markers of the entire musculature (*limpet)*, anterior coelomic sacs (*foxf, foxc)* and posterior coelomic sacs *(twist, hox3)* in *N. anomala* blastula and gastrula embryos treated with 20 μM SU5402 and fixed at the larva stage. (C) WMISH of anterior (*otx, six3/6, nk2.1*), posterior (*nk2.1, hox2, cdx*) posterio-ventral (*foxc*), endodermal (*foxa, cdx, nk2.1*) and markers of musculature (*twist*, *six3/6, foxf)* in *Ph. harmeri* blastula and gastrula embryos treated with 20 μM SU5402 and fixed at the larva stage. The position of the blastopore is indicated with an asterisk. All samples represent at least 2 biological and 2 technical replicates (n=10). Anterior to the top. lv, lateral view. Scale bar: 20 μm.

Development • Supplementary information

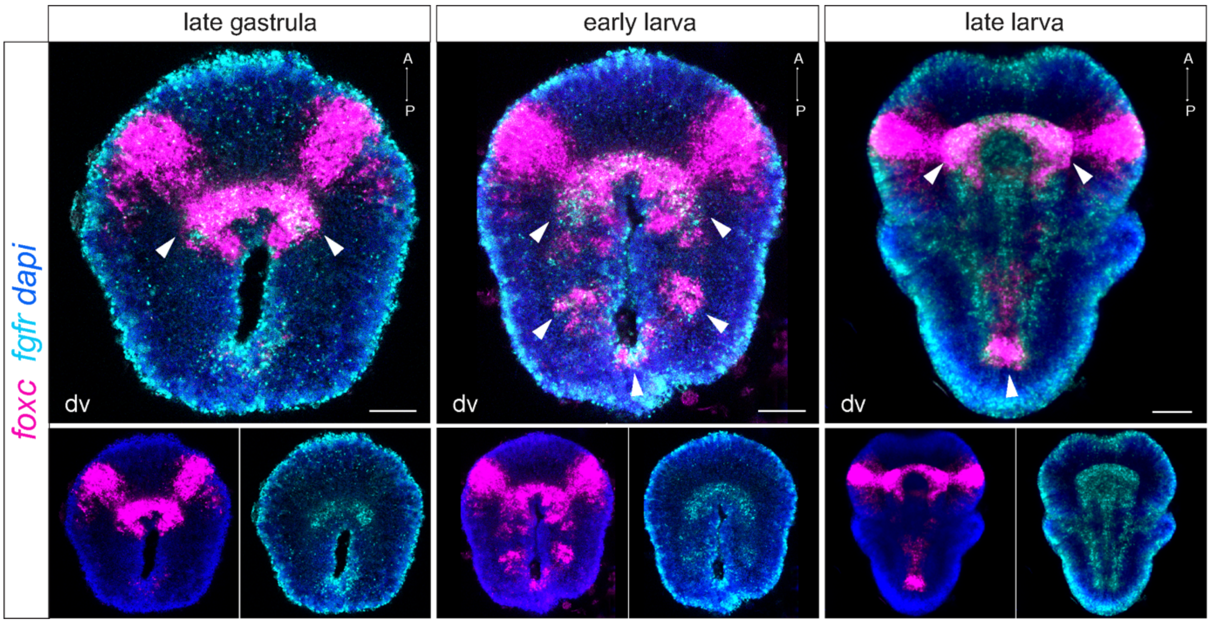

Fig. S9. **Co-expression analysis of *fgfr* (cyan) and *foxc* (magenta) by double fluorescent WMISH in gastrula and larva stages of *T. transversa*.** White arrowheads indicate co-expression. Every fluorescent image is a Z-projection of merged confocal stacks and nuclei are stained with DAPI. Anterior to the top. dv, dorsoventral view. Scale bar: 20 μm.

Development • Supplementary information

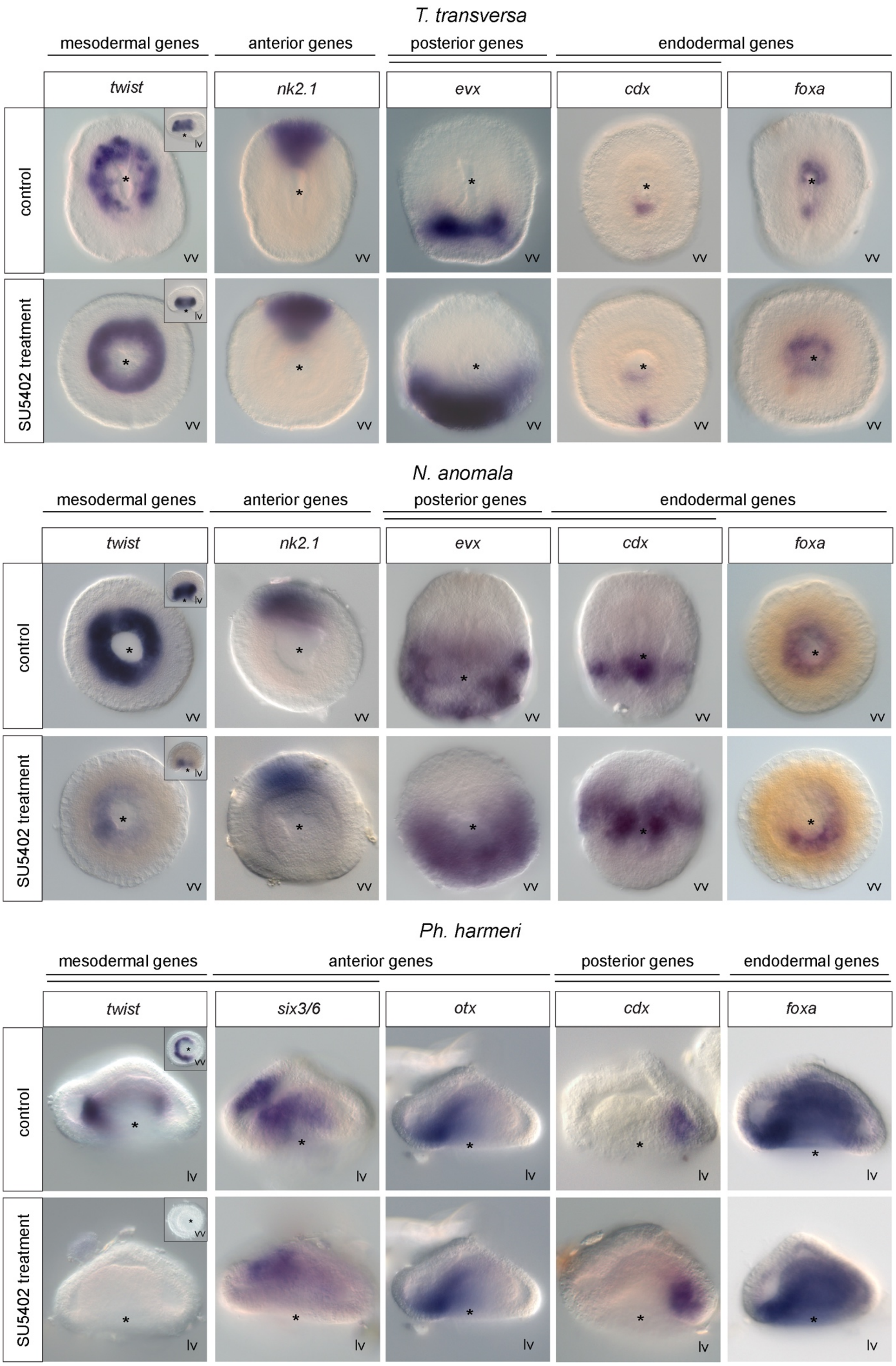

Fig. S10. **WMISH on SU5402 treated *T. transversa, N. anomala* and *Ph. harmeri* embryos fixed at gastrula stage.** WMISH of mesodermal (*twist, six3/6),* anterior (*otx*, *nk2.1*, *six3/6*), posterior (*evx, cdx*) and endodermal genes *(foxa, cdx)* in *T. transversa, N. anomala* and *Ph. harmeri* blastula embryos treated with 20 μM SU5402 and fixed at gastrula stage. The position of the blastopore is indicated with an asterisk. Insets show different views of the embryos. Anterior to the top. lv, lateral view; vv, vegetal view.

Development • Supplementary information

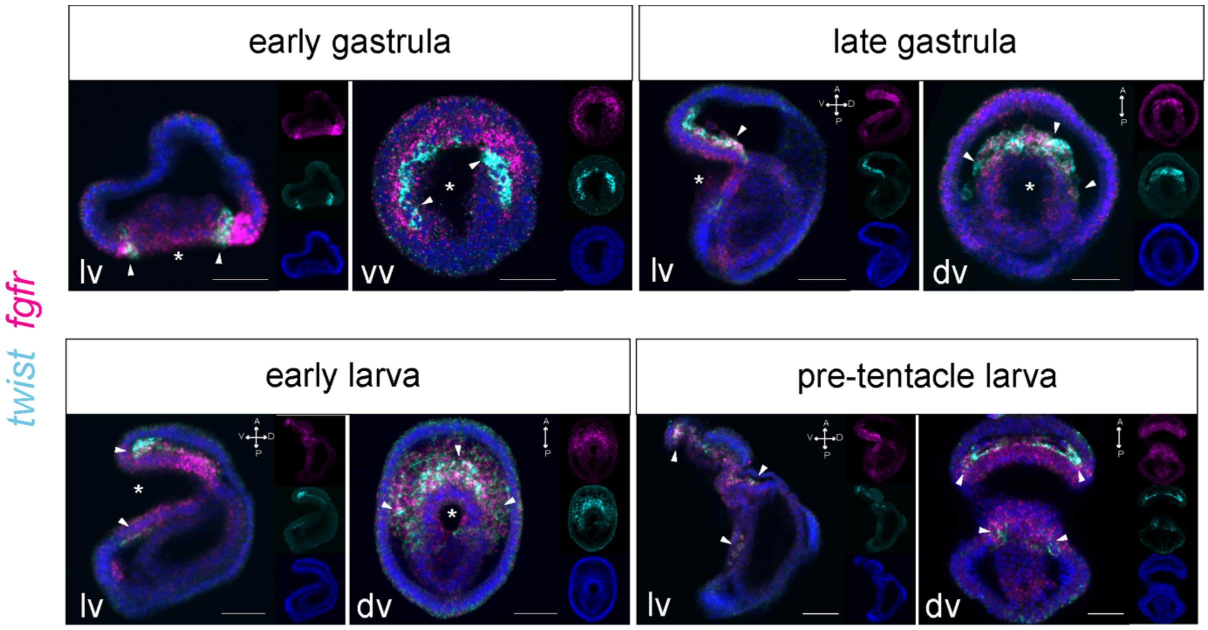

Fig. S11. **Co-expression analysis of *fgfr* (magenta) and *twist* (cyan) by double fluorescent WMISH in gastrula and larva stages of development of *Ph. harmeri*.** White arrowheads indicate co-expression. Every fluorescent image is a Z-projection of merged confocal stacks and nuclei are stained with DAPI. The position of the blastopore is indicated with an asterisk. Anterior to the top. Scale bar: 20 µm.

Development • Supplementary information

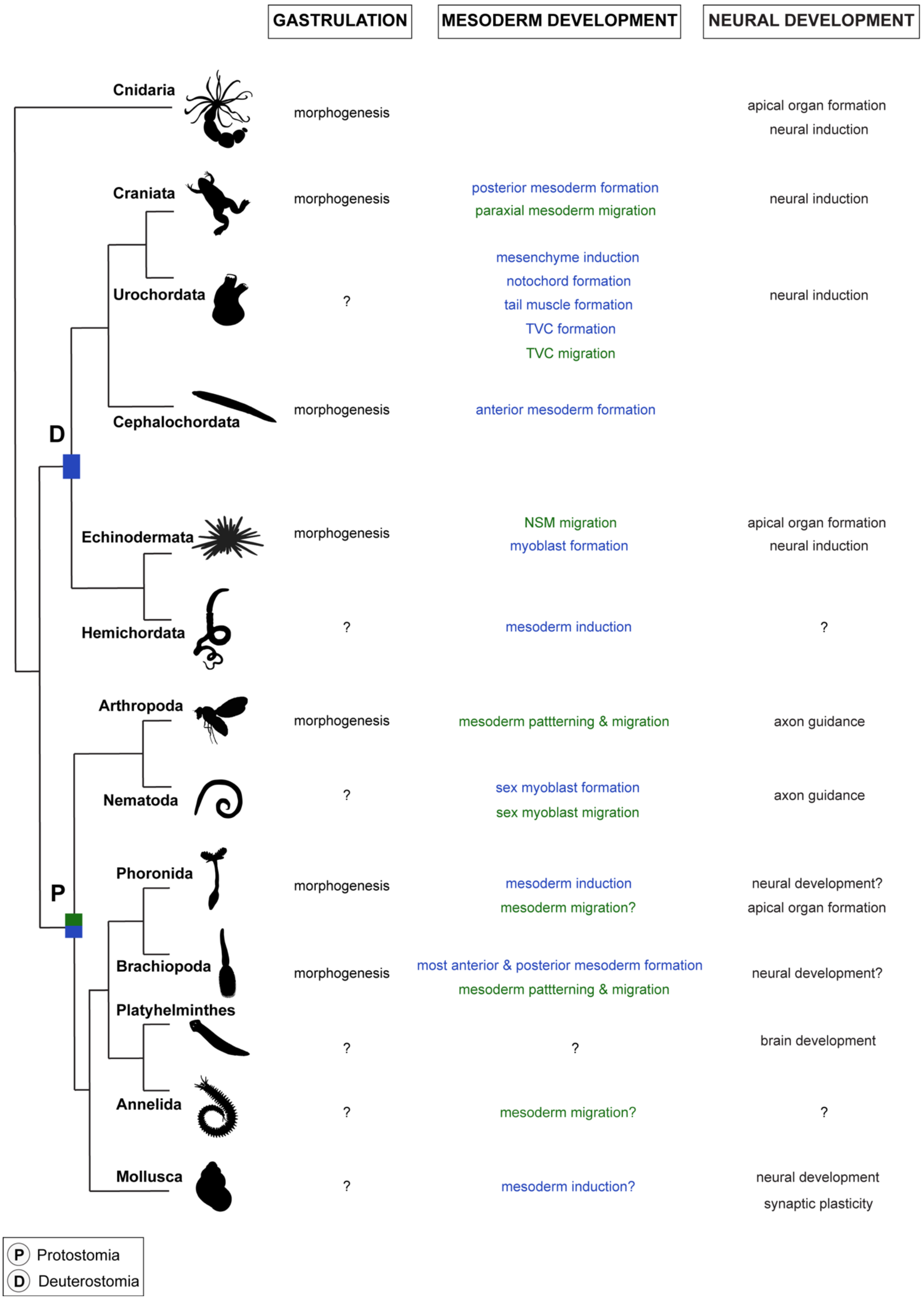

Fig. S12. **The role of FGF signaling in investigated species.** Table summarizing case studies where FGF signaling is found to be upstream of mesoderm development, morphogenetic movements of gastrulation and neural development. Animal illustrations are taken from phylopic.org (CC BY 3.0). D, deuterostomes; NSM, non-skeletogenic mesoderm; P, protostomes; TVC, trunk ventral cells.

Development • Supplementary information

Table S1. Comparison of mesodermal expression patterns and induction signals among Bilateria

Click here to download Table S1

Supplementary references for Table S1

Achim, K., Eling, N., Vergara, H. M., Bertucci, P. Y., Musser, J., Vopalensky, P., Brunet, T., Collier, P., Benes, V., Marioni, J. C., et al. (2018). Whole-Body Single-Cell Sequencing Reveals Transcriptional Domains in the Annelid Larval Body. *Mol Biol Evol* **35**, 1047-1062.

Amin, N. M., Lim, S. E., Shi, H., Chan, T. L. and Liu, J. (2009). A conserved Six-Eya cassette acts downstream of Wnt signaling to direct non-myogenic versus myogenic fates in the *C. elegans* postembryonic mesoderm. *Dev. Biol.* **331**, 350-360.

Amin, N. M., Shi, H. and Liu, J. (2010). The FoxF/FoxC factor LET-381 directly regulates both cell fate specification and cell differentiation in *C. elegans* mesoderm development. *Development* **137**, 1451-1460.

Andrikou, C., Iovene, E., Rizzo, F., Oliveri, P. and Arnone, M. I. (2013). Myogenesis in the sea urchin embryo: the molecular fingerprint of the myoblast precursors. *EvoDevo* **4**, 33.

Andrikou, C., Passamaneck, Y. J., Lowe, C. J., Martindale, M. Q. and Hejnol, A. (2019). Molecular patterning during the development of *Phoronopsis harmeri* reveals similarities to rhynchonelliform brachiopods. *EvoDevo* **10**, 33.

Balagopalan, L., Keller, C. A. and Abmayr, S. M. (2001). Loss-of-function mutations reveal that the Drosophila nautilus gene is not essential for embryonic myogenesis or viability. *Dev Biol* **231**, 374-382.

Baylies, M. K. and Bate, M. (1996). twist: a myogenic switch in Drosophila. *Science* **272**, 1481-1484.

Beach, R. L., Seo, P. and Venuti, J. M. (1999). Expression of the sea urchin MyoD homologue, SUM1, is not restricted to the myogenic lineage during embryogenesis. *Mech Dev* **86**, 209-212.

Beaster-Jones, L., Kaltenbach, S. L., Koop, D., Yuan, S., Chastain, R. and Holland, L. Z. (2008). Expression of somite segmentation genes in amphioxus: a clock without a wavefront? *Dev Genes Evol* **218**, 599-611.

Beh, J., Shi, W., Levine, M., Davidson, B. and Christiaen, L. (2007). FoxF is essential for FGF-induced migration of heart progenitor cells in the ascidian Ciona intestinalis. *Development* **134**, 3297-3305.

Bour, B. A., O'Brien, M. A., Lockwood, W. L., Goldstein, E. S., Bodmer, R., Taghert, P. H., Abmayr, S. M. and Nguyen, H. T. (1995). Drosophila MEF2, a transcription factor that is essential for myogenesis. *Genes Dev* **9**, 730-741.

Brown, D., Wagner, D., Li, X., Richardson, J. A. and Olson, E. N. (1999). Dual role of the basic helix-loop-helix transcription factor scleraxis in mesoderm formation and chondrogenesis during mouse embryogenesis. *Development* **126**, 4317-4329.

Burgess, R., Rawls, A., Brown, D., Bradley, A. and Olson, E. N. (1996). Requirement of the paraxis gene for somite formation and musculoskeletal patterning. *Nature* **384**, 570-573.

Candiani, S., Kreslova, J., Benes, V., Oliveri, D., Castagnola, P., Pestarino, M. and Kozmik, Z. (2003). Cloning and developmental expression of amphioxus Dachschund. *Gene expression patterns : GEP* **3**, 65-69.

Caubit, X., Thangarajah, R., Theil, T., Wirth, J., Nothwang, H. G., Ruther, U. and Krauss, S. (1999). Mouse Dac, a novel nuclear factor with homology to Drosophila dachshund

shows a dynamic expression in the neural crest, the eye, the neocortex, and the limb bud. *Dev Dyn* **214**, 66-80.

Cebrià, F. (2000). Determination, Differentiation and Restitution of the Muscle Pattern During Regeneration and Cell Renewal in Freshwater Planarians. . Barcelona: University of Barcelona.

Chen, Z. F. and Behringer, R. R. (1995). twist is required in head mesenchyme for cranial neural tube morphogenesis. *Genes Dev* **9**, 686-699.

Chu, P. H., Ruiz-Lozano, P., Zhou, Q., Cai, C. and Chen, J. (2000). Expression patterns of FHL/SLIM family members suggest important functional roles in skeletal muscle and cardiovascular system. *Mech Dev* **95**, 259-265.

Cserjesi, P., Lilly, B., Bryson, L., Wang, Y., Sassoon, D. A. and Olson, E. N. (1992). MHox: a mesodermally restricted homeodomain protein that binds an essential site in the muscle creatine kinase enhancer. *Development* **115**, 1087-1101.

Dichoso, D., Brodigan, T., Chwoe, K. Y., Lee, J. S., Llacer, R., Park, M., Corsi, A. K., Kostas, S. A., Fire, A., Ahnn, J., et al. (2000). The MADS-Box factor CeMEF2 is not essential for Caenorhabditis elegans myogenesis and development. *Dev Biol* **223**, 431-440.

Dill, K. K., Thamm, K. and Seaver, E. C. (2007). Characterization of *twist* and *snail* gene expression during mesoderm and nervous system development in the polychaete annelid *Capitella* sp. I. *Dev. Genes Evol.* **217**, 435-447.

Fan, T. P., Ting, H. C., Yu, J. K. and Su, Y. H. (2018). Reiterative use of FGF signaling in mesoderm development during embryogenesis and metamorphosis in the hemichordate *Ptychodera flava*. *BMC Evol. Biol.* **18**, 120.

Furuya, M., Qadota, H., Chisholm, A. D. and Sugimoto, A. (2005). The C. elegans eyes absent ortholog EYA-1 is required for tissue differentiation and plays partially redundant roles with PAX-6. *Dev Biol* **286**, 452-463.

Gabriel, W. N., McNuff, R., Patel, S. K., Gregory, T. R., Jeck, W. R., Jones, C. D. and Goldstein, B. (2007). The tardigrade Hypsibius dujardini, a new model for studying the evolution of development. *Dev Biol* **312**, 545-559.

Good, K., Ciosk, R., Nance, J., Neves, A., Hill, R. J. and Priess, J. R. (2004). The T-box transcription factors TBX-37 and TBX-38 link GLP-1/Notch signaling to mesoderm induction in *C. elegans* embryos. *Development* **131**, 1967-1978.

Green, S. A., Norris, R. P., Terasaki, M. and Lowe, C. J. (2013). FGF signaling induces mesoderm in the hemichordate *Saccoglossus kowalevskii*. *Development* **140**, 1024-1033.

Grifone, R., Demignon, J., Houbron, C., Souil, E., Niro, C., Seller, M. J., Hamard, G. and Maire, P. (2005). Six1 and Six4 homeoproteins are required for Pax3 and Mrf expression during myogenesis in the mouse embryo. *Development* **132**, 2235-2249.

Hacker, U., Kaufmann, E., Hartmann, C., Jurgens, G., Knochel, W. and Jackle, H. (1995). The Drosophila fork head domain protein crocodile is required for the establishment of head structures. *EMBO J* **14**, 5306-5317.

Hammond, K. L., Hill, R. E., Whitfield, T. T. and Currie, P. D. (2002). Isolation of three zebrafish dachshund homologues and their expression in sensory organs, the central nervous system and pectoral fin buds. *Mech Dev* **112**, 183-189.

Harfe, B. D., Branda, C. S., Krause, M., Stern, M. J. and Fire, A. (1998a). MyoD and the specification of muscle and non-muscle fates during postembryonic development of the *C. elegans* mesoderm. *Development* **125**, 2479-2488.

Harfe, B. D., Vaz Gomes, A., Kenyon, C., Liu, J., Krause, M. and Fire, A. (1998b). Analysis of a Caenorhabditis elegans Twist homolog identifies conserved and divergent aspects of mesodermal patterning. *Genes Dev* **12**, 2623-2635.

He, G., Tavella, S., Hanley, K. P., Self, M., Oliver, G., Grifone, R., Hanley, N., Ward, C. and Bobola, N. (2010). Inactivation of Six2 in mouse identifies a novel genetic mechanism controlling development and growth of the cranial base. *Dev Biol* **344**, 720-730.

Heanue, T. A., Reshef, R., Davis, R. J., Mardon, G., Oliver, G., Tomarev, S., Lassar, A. B. and Tabin, C. J. (1999). Synergistic regulation of vertebrate muscle development by Dach2, Eya2, and Six1, homologs of genes required for Drosophila eye formation. *Genes Dev* **13**, 3231-3243.

Hinman, V. F. and Degnan, B. M. (2002). Mox homeobox expression in muscle lineage of the gastropod *Haliotis asinina*: evidence for a conserved role in bilaterian myogenesis. *Dev. Genes Evol.* **212**, 141-144.

Howard-Ashby, M., Materna, S. C., Brown, C. T., Chen, L., Cameron, R. A. and Davidson, E. H. (2006). Gene families encoding transcription factors expressed in early development of Strongylocentrotus purpuratus. *Dev Biol* **300**, 90-107.

Imai, K. S., Hino, K., Yagi, K., Satoh, N. and Satou, Y. (2004). Gene expression profiles of transcription factors and signaling molecules in the ascidian embryo: towards a comprehensive understanding of gene networks. *Development* **131**, 4047-4058.

Imai, K. S., Satoh, N. and Satou, Y. (2002). Early embryonic expression of *FGF4*/6/9 gene and its role in the induction of mesenchyme and notochord in *Ciona savignyi* embryos. *Development* **129**, 1729-1738.

Imai, K. S., Satoh, N. and Satou, Y. (2003). A Twist-like bHLH gene is a downstream factor of an endogenous FGF and determines mesenchymal fate in the ascidian embryos. *Development* **130**, 4461-4472.

Ip, Y. T., Park, R. E., Kosman, D., Yazdanbakhsh, K. and Levine, M. (1992). dorsal-twist interactions establish snail expression in the presumptive mesoderm of the Drosophila embryo. *Genes Dev* **6**, 1518-1530.

Jiang, J., Kosman, D., Ip, Y. T. and Levine, M. (1991). The dorsal morphogen gradient regulates the mesoderm determinant twist in early *Drosophila* embryos. *Genes Dev.* **5**, 1881-1891.

Kiecker, C., Bates, T. and Bell, E. (2016). Molecular specification of germ layers in vertebrate embryos. *Cell Mol Life Sci* **73**, 923-947.

Kim, G. J. and Nishida, H. (2001). Role of the FGF and MEK signaling pathway in the ascidian embryo. *Dev. Growth Differ.* **43**, 521-533.

Kimelman, D. (2006). Mesoderm induction: from caps to chips. *Nat. Rev. Genet.* **7**, 360-372.

Knirr, S., Azpiazu, N. and Frasch, M. (1999). The role of the NK-homeobox gene slouch (S59) in somatic muscle patterning. *Development* **126**, 4525-4535.

Kobayashi, A., Valerius, M. T., Mugford, J. W., Carroll, T. J., Self, M., Oliver, G. and McMahon, A. P. (2008). Six2 defines and regulates a multipotent self-renewing nephron progenitor population throughout mammalian kidney development. *Cell Stem Cell* **3**, 169-181.

Koop, D., Richards, G. S., Wanninger, A., Gunter, H. M. and Degnan, B. M. (2007). The role of MAPK signaling in patterning and establishing axial symmetry in the gastropod *Haliotis asinina*. *Dev. Biol.* **311**, 200-212.

Development • Supplementary information

Kozin, V. and Kostyuchenko, R. (2016). Evolutionary conservation and variability of the mesoderm development in spiralia: A peculiar pattern of nereid polychaetes. *Biol. Bull.* **43**, 216-225.

Kozmik, Z., Holland, N. D., Kreslova, J., Oliveri, D., Schubert, M., Jonasova, K., Holland, L. Z., Pestarino, M., Benes, V. and Candiani, S. (2007). Pax-Six-Eya-Dach network during amphioxus development: conservation in vitro but context specificity in vivo. *Dev. Biol.* **306**, 143-159.

Kume, T., Jiang, H., Topczewska, J. M. and Hogan, B. L. (2001). The murine winged helix transcription factors, Foxc1 and Foxc2, are both required for cardiovascular development and somitogenesis. *Genes Dev* **15**, 2470-2482.

Lambert, J. D. (2008). Mesoderm in spiralians: the organizer and the 4d cell. *J. Exp. Zool. B. Mol. Dev. Evol.* **310**, 15-23.

Lambert, J. D. and Nagy, L. M. (2001). MAPK signaling by the D quadrant embryonic organizer of the mollusc *Ilyanassa obsoleta*. *Development* **128**, 45-56.

Lambert, J. D. and Nagy, L. M. (2003). The MAPK cascade in equally cleaving spiralian embryos. *Dev. Biol.* **263**, 231-241.

Lauri, A., Brunet, T., Handberg-Thorsager, M., Fischer, A. H., Simakov, O., Steinmetz, P. R., Tomer, R., Keller, P. J. and Arendt, D. (2014). Development of the annelid axochord: insights into notochord evolution. *Science* **345**, 1365-1368.

Leptin, M. (1991). twist and snail as positive and negative regulators during Drosophila mesoderm development. *Genes Dev* **5**, 1568-1576.

Leussink, B., Brouwer, A., el Khattabi, M., Poelmann, R. E., Gittenberger-de Groot, A. C. and Meijlink, F. (1995). Expression patterns of the paired-related homeobox genes MHox/Prx1 and S8/Prx2 suggest roles in development of the heart and the forebrain. *Mech Dev* **52**, 51-64.

Liu, Y. H., Jakobsen, J. S., Valentin, G., Amarantos, I., Gilmour, D. T. and Furlong, E. E. (2009). A systematic analysis of Tinman function reveals Eya and JAK-STAT signaling as essential regulators of muscle development. *Dev Cell* **16**, 280-291.

Lowe, C. J., Terasaki, M., Wu, M., Freeman, R. M., Jr., Runft, L., Kwan, K., Haigo, S., Aronowicz, J., Lander, E., Gruber, C., et al. (2006). Dorsoventral patterning in hemichordates: insights into early chordate evolution. *PLoS Biol* **4**, e291.

Lu, M. F., Cheng, H. T., Kern, M. J., Potter, S. S., Tran, B., Diekwisch, T. G. and Martin, J. F. (1999). prx-1 functions cooperatively with another paired-related homeobox gene, prx-2, to maintain cell fates within the craniofacial mesenchyme. *Development* **126**, 495-504.

Mahlapuu, M., Ormestad, M., Enerback, S. and Carlsson, P. (2001). The forkhead transcription factor Foxf1 is required for differentiation of extra-embryonic and lateral plate mesoderm. *Development* **128**, 155-166.

Mankoo, B. S., Collins, N. S., Ashby, P., Grigorieva, E., Pevny, L. H., Candia, A., Wright, C. V., Rigby, P. W. and Pachnis, V. (1999). Mox2 is a component of the genetic hierarchy controlling limb muscle development. *Nature* **400**, 69-73.

Mankoo, B. S., Skuntz, S., Harrigan, I., Grigorieva, E., Candia, A., Wright, C. V., Arnheiter, H. and Pachnis, V. (2003). The concerted action of Meox homeobox genes is required upstream of genetic pathways essential for the formation, patterning and differentiation of somites. *Development* **130**, 4655-4664.

Martin, B., Schneider, R., Janetzky, S., Waibler, Z., Pandur, P., Kuhl, M., Behrens, J., von der Mark, K., Starzinski-Powitz, A. and Wixler, V. (2002). The LIM-only protein FHL2

interacts with beta-catenin and promotes differentiation of mouse myoblasts. *J Cell Biol* **159**, 113-122.

Martín-Durán, J., Passamaneck, Y. J., Martindale, M. Q. and Hejnol, A. (2016). The developmental basis for the recurrent evolution of deuterostomy and protostomy. *Nat. Ecol. Evol.* **1**, 5.

Martin-Duran, J. M., Amaya, E. and Romero, R. (2010). Germ layer specification and axial patterning in the embryonic development of the freshwater planarian Schmidtea polychroa. *Dev Biol* **340**, 145-158.

Materna, S. C. and Davidson, E. H. (2012). A comprehensive analysis of Delta signaling in pre-gastrular sea urchin embryos. *Dev. Biol.* **364**, 77-87.

Materna, S. C., Ransick, A., Li, E. and Davidson, E. H. (2013). Diversification of oral and aboral mesodermal regulatory states in pregastrular sea urchin embryos. *Dev. Biol.* **375**, 92-104.

Mazet, F., Amemiya, C. T. and Shimeld, S. M. (2006). An ancient Fox gene cluster in bilaterian animals. *Current biology : CB* **16**, R314-316.

Mazet, F., Hutt, J. A., Milloz, J., Millard, J., Graham, A. and Shimeld, S. M. (2005). Molecular evidence from Ciona intestinalis for the evolutionary origin of vertebrate sensory placodes. *Dev Biol* **282**, 494-508.

Meedel, T. H., Chang, P. and Yasuo, H. (2007). Muscle development in Ciona intestinalis requires the b-HLH myogenic regulatory factor gene Ci-MRF. *Dev Biol* **302**, 333-344.

Minguillon, C. and Garcia-Fernandez, J. (2002). The single amphioxus Mox gene: insights into the functional evolution of Mox genes, somites, and the asymmetry of amphioxus somitogenesis. *Dev Biol* **246**, 455-465.

Molkentin, J. D., Black, B. L., Martin, J. F. and Olson, E. N. (1995). Cooperative activation of muscle gene expression by MEF2 and myogenic bHLH proteins. *Cell* **83**, 1125-1136.

Morgan, M. J. and Madgwick, A. J. (1999). The LIM proteins FHL1 and FHL3 are expressed differently in skeletal muscle. *Biochem Biophys Res Commun* **255**, 245-250.

Nederbragt, A. J., Lespinet, O., van Wageningen, S., van Loon, A. E., Adoutte, A. and Dictus, W. J. (2002). A lophotrochozoan twist gene is expressed in the ectomesoderm of the gastropod mollusk *Patella vulgata*. *Evol. Dev.* **4**, 334-343.

Niro, C., Demignon, J., Vincent, S., Liu, Y., Giordani, J., Sgarioto, N., Favier, M., Guillet-Deniau, I., Blais, A. and Maire, P. (2010). Six1 and Six4 gene expression is necessary to activate the fast-type muscle gene program in the mouse primary myotome. *Dev Biol* **338**, 168-182.

Oliver, G., Wehr, R., Jenkins, N. A., Copeland, N. G., Cheyette, B. N., Hartenstein, V., Zipursky, S. L. and Gruss, P. (1995). Homeobox genes and connective tissue patterning. *Development* **121**, 693-705.

Opstelten, D. J., Vogels, R., Robert, B., Kalkhoven, E., Zwartkruis, F., de Laaf, L., Destree, O. H., Deschamps, J., Lawson, K. A. and Meijlink, F. (1991). The mouse homeobox gene, S8, is expressed during embryogenesis predominantly in mesenchyme. *Mech Dev* **34**, 29-41.

Ormestad, M., Astorga, J., Landgren, H., Wang, T., Johansson, B. R., Miura, N. and Carlsson, P. (2006). Foxf1 and Foxf2 control murine gut development by limiting mesenchymal Wnt signaling and promoting extracellular matrix production. *Development* **133**, 833-843.

Passamaneck, Y. J., Hejnol, A. and Martindale, M. Q. (2015). Mesodermal gene expression during the embryonic and larval development of the articulate brachiopod *Terebratalia transversa*. *EvoDevo* **6**, 10.

Perry, K. J., Lyons, D. C., Truchado-Garcia, M., Fischer, A. H., Helfrich, L. W., Johansson, K. B., Diamond, J. C., Grande, C. and Henry, J. Q. (2015). Deployment of regulatory genes during gastrulation and germ layer specification in a model spiralian mollusc *Crepidula*. *Dev. Dyn.* **244**, 1215-1248.

Pfeifer, K., Schaub, C., Wolfstetter, G. and Dorresteijn, A. (2013). Identification and characterization of a twist ortholog in the polychaete annelid Platynereis dumerilii reveals mesodermal expression of Pdu-twist. *Dev Genes Evol* **223**, 319-328.

Potthoff, M. J., Arnold, M. A., McAnally, J., Richardson, J. A., Bassel-Duby, R. and Olson, E. N. (2007). Regulation of skeletal muscle sarcomere integrity and postnatal muscle function by Mef2c. *Mol Cell Biol* **27**, 8143-8151.

Potthoff, M. J. and Olson, E. N. (2007). MEF2: a central regulator of diverse developmental programs. *Development* **134**, 4131-4140.

Price, A. L. and Patel, N. H. (2008). Investigating divergent mechanisms of mesoderm development in arthropods: the expression of Ph-twist and Ph-mef2 in Parhyale hawaiensis. *J Exp Zool B Mol Dev Evol* **310**, 24-40.

Qadota, H., Mercer, K. B., Miller, R. K., Kaibuchi, K. and Benian, G. M. (2007). Two LIM domain proteins and UNC-96 link UNC-97/pinch to myosin thick filaments in Caenorhabditis elegans muscle. *Mol Biol Cell* **18**, 4317-4326.

Ranganayakulu, G., Zhao, B., Dokidis, A., Molkentin, J. D., Olson, E. N. and Schulz, R. A. (1995). A series of mutations in the D-MEF2 transcription factor reveal multiple functions in larval and adult myogenesis in Drosophila. *Dev Biol* **171**, 169-181.

Rudnicki, M. A., Schnegelsberg, P. N., Stead, R. H., Braun, T., Arnold, H. H. and Jaenisch, R. (1993). MyoD or Myf-5 is required for the formation of skeletal muscle. *Cell* **75**, 1351-1359.

Saudemont, A., Dray, N., Hudry, B., Le Gouar, M., Vervoort, M. and Balavoine, G. (2008). Complementary striped expression patterns of NK homeobox genes during segment formation in the annelid Platynereis. *Dev Biol* **317**, 430-443.

Schubert, M., Meulemans, D., Bronner-Fraser, M., Holland, L. Z. and Holland, N. D. (2003). Differential mesodermal expression of two amphioxus MyoD family members (AmphiMRF1 and AmphiMRF2). *Gene Expr. Patterns* **3**, 199-202.

Scimone, M. L., Kravarik, K. M., Lapan, S. W. and Reddien, P. W. (2014). Neoblast specialization in regeneration of the planarian Schmidtea mediterranea. *Stem Cell Reports* **3**, 339-352.

Shimeld, S. M., Boyle, M. J., Brunet, T., Luke, G. N. and Seaver, E. C. (2010). Clustered Fox genes in lophotrochozoans and the evolution of the bilaterian Fox gene cluster. *Dev. Biol.* **340**, 234-248.

Steinmetz, P. R. H. (2006). Comparative molecular and morphogenetic characterisation of larval body regions in the polychaete annelid *Platynereis dumerilii*. In *Biologie*: Philipps-Universität Marburg.

Sweet, H. C., Gehring, M. and Ettensohn, C. A. (2002). LvDelta is a mesoderm-inducing signal in the sea urchin embryo and can endow blastomeres with organizer-like properties. *Development* **129**, 1945-1955.

Development • Supplementary information

ten Berge, D., Brouwer, A., Korving, J., Martin, J. F. and Meijlink, F. (1998). Prx1 and Prx2 in skeletogenesis: roles in the craniofacial region, inner ear and limbs. *Development* **125**, 3831-3842.

Tokuoka, M., Satoh, N. and Satou, Y. (2005). A bHLH transcription factor gene, Twist-like 1, is essential for the formation of mesodermal tissues of Ciona juveniles. *Dev Biol* **288**, 387-396.

Tomancak, P., Berman, B. P., Beaton, A., Weiszmann, R., Kwan, E., Hartenstein, V., Celniker, S. E. and Rubin, G. M. (2007). Global analysis of patterns of gene expression during Drosophila embryogenesis. *Genome Biol* **8**, R145.

Tu, Q., Brown, C. T., Davidson, E. H. and Oliveri, P. (2006). Sea urchin Forkhead gene family: phylogeny and embryonic expression. *Dev Biol* **300**, 49-62.

Vellutini, B. C., Martín-Durán, J. M. and Hejnol, A. (2017). Cleavage modification did not alter blastomere fates during bryozoan evolution. *BMC Biol.* **15**, 33.

Winchell, C. J., Valencia, J. E. and Jacobs, D. K. (2010). Expression of Distal-less, dachshund, and optomotor blind in Neanthes arenaceodentata (Annelida, Nereididae) does not support homology of appendage-forming mechanisms across the Bilateria. *Dev Genes Evol* **220**, 275-295.

Winnier, G. E., Hargett, L. and Hogan, B. L. (1997). The winged helix transcription factor MFH1 is required for proliferation and patterning of paraxial mesoderm in the mouse embryo. *Genes Dev* **11**, 926-940.

Wu, S. Y., Yang, Y. P. and McClay, D. R. (2008). Twist is an essential regulator of the skeletogenic gene regulatory network in the sea urchin embryo. *Dev Biol* **319**, 406-415.

Xiong, G., Qadota, H., Mercer, K. B., McGaha, L. A., Oberhauser, A. F. and Benian, G. M. (2009). A LIM-9 (FHL)/SCPL-1 (SCP) complex interacts with the C-terminal protein kinase regions of UNC-89 (obscurin) in Caenorhabditis elegans muscle. *J Mol Biol* **386**, 976-988.

Xu, P. X., Adams, J., Peters, H., Brown, M. C., Heaney, S. and Maas, R. (1999). Eya1-deficient mice lack ears and kidneys and show abnormal apoptosis of organ primordia. *Nat Genet* **23**, 113-117.

Yasui, K., Zhang, S. C., Uemura, M., Aizawa, S. and Ueki, T. (1998). Expression of a twist-related gene, Bbtwist, during the development of a lancelet species and its relation to cephalochordate anterior structures. *Dev Biol* **195**, 49-59.

Zaffran, S., Kuchler, A., Lee, H. H. and Frasch, M. (2001). biniou (FoxF), a central component in a regulatory network controlling visceral mesoderm development and midgut morphogenesis in *Drosophila*. *Genes Dev.* **15**, 2900-2915.

Zhang, Y., Wang, L., Shao, M. and Zhang, H. (2007). Characterization and developmental expression of AmphiMef2 gene in amphioxus. *Sci China C Life Sci* **50**, 637-641.

Table S2 . Primers, chosen regions of probes and probe size for investigated genes

Click here to download Table S2
