# Supplementary material for: FGF signaling acts on different levels of mesoderm development within Spiralia
Source: Development. 2021 May 17;148(10):dev196089. doi: 10.1242/dev.196089 (PMC8180254; doi:10.1242/dev.196089)
Supplement: Reviewer comments [file develop-148-196089_review_history.pdf]

FGF signaling acts on different levels of mesoderm development within Spiralia

Carmen Andrikou and Andreas Hejnol
DOI: 10.1242/dev.196089

Editor: James Briscoe

Review timeline

Original submission:	17 August 2020
Editorial decision:	16 October 2020
First revision received:	16 February 2021
Editorial decision:	12 March 2021
Second revision received:	7 April 2021
Accepted:	8 April 2021

Original submission

First decision letter

MS ID#: DEVELOP/2020/196089

MS TITLE: FGF signaling induces mesoderm in members of Spiralia

AUTHORS: Carmen Andrikou and Andreas Hejnol

I apologise that it has taken longer than normal to get back to you, but one of the referees was delayed in returning their report. I have now received all the referees' reports on the above manuscript, and have reached a decision. The referees' comments are appended below, or you can access them online: please go to BenchPress and click on the 'Manuscripts with Decisions' queue in the Author Area.

As you will see, the referees express considerable interest in your work, but have some significant criticisms and recommend a substantial revision of your manuscript before we can consider publication. All three referees have constructive criticisms about the presentation and explanation of the data. They identify places where they would like the results to be more accurately and precisely described, and the findings placed in context of what is already known. In addition, the referees raise several issues about the interpretation of some of the data that should be addressed. Experimentally, the main issue is the specificity of SU5402. Including experimental evidence that SU5042 inhibits FGF signaling in the species you are studying would address this concern.

If you are able to revise the manuscript along the lines suggested, which is likely to involve further experiments, I will be happy receive a revised version of the manuscript. Your revised paper will be re-reviewed by one or more of the original referees, and acceptance of your manuscript will depend on your addressing satisfactorily the reviewers' major concerns. Please also note that Development will normally permit only one round of major revision.

We are aware that you may be experiencing disruption to the normal running of your lab that make experimental revisions challenging. If it would be helpful, we encourage you to contact us to discuss your revision in greater detail. Please send us a point-by-point response indicating where you are able to address concerns raised (either experimentally or by changes to the text) and

© 2021. Published by The Company of Biologists under the terms of the Creative Commons Attribution License (https://creativecommons.org/licenses/by/4.0/).

where you will not be able to do so within the normal timeframe of a revision. We will then provide further guidance. Please also note that we are happy to extend revision timeframes as necessary.

Please attend to all of the reviewers' comments and ensure that you clearly highlight all changes made in the revised manuscript. Please avoid using 'Tracked changes' in Word files as these are lost in PDF conversion. I should be grateful if you would also provide a point-by-point response detailing how you have dealt with the points raised by the reviewers in the 'Response to Reviewers' box. If you do not agree with any of their criticisms or suggestions please explain clearly why this is so.

Reviewer 1

Advance summary and potential significance to field

In their article entitled “FGF signalling induces mesoderm in members of Spiralia”, Carmen Andrikou and Andreas Hejnol describe the expression pattern of genes known as mesodermal/muscle markers in other bilaterian animals in one brachiopod and one phoronid species. They then assess the role of FGF signalling in the formation of the mesoderm in these species and in another brachiopod species by looking at the expression of the receptor and ligand genes during development, and by using pharmacological treatments with SU5402. FGF has been shown to be required at different steps of mesoderm or mesodermal derivatives formation in diverse bilaterian phyla (deuterostomes and ecdysozoans) and the work presented here brings potentially some indications suggesting that this signalling pathway would also play similar although variable roles in members of the Spiralia group. The authors present many data, most of them technically sounds. Although the question is extremely interesting and the use of non-classical models brings new insights into our understanding of mesoderm evolution in bilaterian, I have some major concerns detailed bellow.

Comments for the author

- Major global concerns:

1. All through the manuscript, there are some inaccuracies on the use of the term “mesoderm”. The mesoderm is a germ layer that during development form some specific structures among which muscles, but not only. The authors often use mesoderm to describe muscles, as for example in Figure 1: the actin immunostaining show the muscles and not the mesoderm that at the larval stage does not exist anymore.
2. Another concern is the use of the term induction. Induction of the mesoderm corresponds to the phenomenon through which cells (usually for a mesendodermal precursor) receive a signal, coming from other cells, that allows them to adopt a mesodermal fate. Further processes such as patterning, migration or differentiation are not considered as an induction phenomenon. Here the authors often inaccurately refer to the term induction. For example, in the abstract, the authors write “FGF signalling is involved in mesoderm induction in deuterostomes”: this is quite incorrect as it is not the case in many deuterostomes although FGF signal is implicated at different steps of mesoderm development in all deuterostomes. The whole manuscript should be corrected by using the precise and appropriate terms when speaking about mesoderm formation. This is crucial in order to really give the possibility to compare the role of signalling pathways in the different steps of this process between metazoan lineages.
3. The use of GRN is also often inappropriate. GRN refers to genes for which a functional interaction has been demonstrated. In their work the authors describe transcription factor toolkit rather than GRN (for example page 4 end of the introduction).
4. SU5402 is an inhibitor of the vertebrate FGFR1. It has been shown to inhibit the FGF signalling pathway in other deuterostomes through the use of other molecules that would impact this pathway at a different steps (for example MAPK inhibitors) or by showing that it is downregulating the expression of genes orthologous to known FGF signalling pathway targets in vertebrates. In their article, the authors do not test in any way the real potentiality of SU5402 to inhibit the FGF signalling pathway. It is also to note that the authors propose the species studied to have several FGF receptor genes (2 or 3 in the brachiopods). However, the phylogenetic tree presented (Supp Data) is not very well supported and fails to include sequences of receptors that were characterized as being closely related to FGFR and PDVEGFR families but that are neither members of FGFR nor PDVEGFR groups and that were called ExTK (see D’Aniello et al., 2008). The

© 2021. Published by The Company of Biologists under the terms of the Creative Commons Attribution License (https://creativecommons.org/licenses/by/4.0/).

authors should be more careful in assigning the FGFR name to the RTK genes described here as from the phylogeny presented some FGFR might rather belong to this ExTK group. Overall, it seems hard from the presented data to support the fact that the phenotypes observed after treatment are due to the inhibition of the FGF pathway.

- Specific comments on the text:

5. In the introduction the authors write: “deuterostomes generally form mesoderm by outpouchings of their invaginating endoderm (archenteron), a mechanism named enterocoely”. Enterocoely refers to the formation of the coelom and not the mesoderm per se.
6. page 4 first line: replace “mesoderm” by “muscles”.
7. page 4 second line: 4 coelomic sacs are described for T. transversa, but it rather seems that they have only one, at least from Fig 1 and from all the other figures concerning this species.
8. page 4 last sentence, the authors write “Despite our observed differences in the presumptive mesodermal gene regulatory networks (GRN), our results indicate a conservation of the inductive role of FGF signalling pathway in mesoderm with deuterostomes.”. As I said before, here there is no analysis of GRN, but rather of TF toolkit, and the authors are not demonstrating a putative mesoderm inductive capacity at least for the brachiopods studied here for which SU5402 treatment alters mesoderm formation but not its induction if there is any.
9. page 5: I would rather say that “indicating that mesoderm is specified” rather than “indicating that mesoderm originates”.
10. page 6: “in N. anomala it is expressed in most of the mesoderm but acquires a stronger expression at the posterior one (cs4)”: Replace by “in N. anomala it is expressed in most of the mesoderm but acquires a stronger expression in the posterior coelomic sac (cs4)”
11. page 6: “Dachs seems to be absent from the anterior mesodermal pattering in Ph. harmeri”: rather replace by “Dachs is not expressed during anterior mesodermal patterning in Ph. harmeri”.
12. page 6: “it mainly occupies anterior (apical) mesodermal fates”: transcripts do not occupy a fate.
13. page 9: the expression of fgfr1 in anterior scattered cells for T. transversa is not visible and not indicated on the WISH pictures presented (Fig4A4).
14. page 9: “The analysis of the spatial expression of the three receptors and the two ligands suggests a putative involvement of FGFR1 and FGF8/17/18 in mesoderm development (see co-expression of fgfr1 and fgf8/17/18 in Fig. S4)”. I don’t see any overlap of expression between fgfr1 and fgf8/17/18.
15. page 10: it is not clear if “cs” corresponds to coelomic sac or chaete sacs. Please specify all through the manuscript.
16. page 12: The effects observed for the treatment starting at the blastula stage in T. transversa suggest a failure of correct gastrulation movements rather than a failure in axial elongation as no blastopore is formed.
17. page 16: “To test whether the role of FGF signalling in mesoderm induction extends beyond the brachiopod lineage..”: the authors did not show a failure in mesoderm induction after SU5402 treatment although they show it affects mesoderm and mesodermal structures development.
18. page 16: “To test wether this was due”: please explain to what “this”refers.
19. page 16: “These results show that FGF signalling is upstream of anteroposterior patterning”: I don’t see which result shows this implication. It is not because the expression of two genes (normally expressed anteriorly in the mouth or posteriorly) is abolished that the A/P patterning is affected. The expression of A/P patterning genes should be tested to answer this point.
20. page 20: second paragraph: I think comparing FGFR1 and FGF8/17/18 potentially conserved role in posterior elongation between vertebrates and brachiopods, from the data presented, is taking a shortcut that should not be included in the discussion. It is clear that even among ecdysozoans, and among deuterostomes, “posterior elongation” has different meanings and implies different biological processes. Moreover the authors write: “The role of FGF signalling in the posterior axial elongation of T. transversa resembles mechanisms typically deployed by a number of deuterostomes in order to coordinate embryonic patterning and elongation of posterior tissues: in the tunicate species Ciona intestinalis, FGF, together with the canonical WNT and Retinoic Acid (RA) signalling, are establishing the posterior patterning of the tail (Pasini et al., 2012), in amphioxus FGFand RA are coordinating the posterior elongation (Bertrand et al., 2015)” and are therefore mixing posterior growth and patterning of posterior tissues. In ascidians, what has been shown in Pasini et al. is not associated to posterior elongation. In cephalochordates, RA and FGF are not controlling posterior elongation in contrary to what is known in vertebrates.

© 2021. Published by The Company of Biologists under the terms of the Creative Commons Attribution License (https://creativecommons.org/licenses/by/4.0/).

21. p20-p21: “A coordinated interplay of WNT and FGF signalling in regulating posterior axial elongation seems also likely, suggesting a putative conserved developmental signalling mechanism in orchestrating posterior regionalization. “. To my opinion there are no data presented in the manuscript that could support such proposition.
22. p21: please explain what is “presursor 3D”
23. p22: for phylogenetic reconstructions, it is indicated that ML inference was used in the material and methods section whereas in the FigS1 legend the authors indicate they used Bayesian inference. Given the numbers indicated as supports in the trees, it might have been a ML analysis. Please clarify this point.
24. p22: SU5402 treatment: I believe SU5402 was first dissolve in DMSO at a high concentration to prepare a stock solution and then dissolved in sea water at the indicated concentrations.
25. p22: please specify what PBT and PBX mean.
26. p22-p23: please indicate the dilution used for the antibodies.

• Figure comments:
27. In most of the figures some information are missing: scale bars, full orientation of the embryos/larva (dorsal part for lateral views?). Moreover, for larva, I imagine that vv does not mean vegetal view but rather ventral view.
28. Some inserts are not visible (too small).
29. Inserts in Fig.4, corresponding to fluorescent in situ hybridization are very small. Moreover, after zooming they do not show the same results as with the chromogenic in situ hybridization: this should be explained and commented, or chromogenic in situ hybridization should be undertaken again.
30. In almost all the figures, SU5402 is written SU502.
31. It is not clear why muscles are detected thanks to actin, tropomyosin or HB9 immunostaining (for example between Fig. 5 and Fig. S8). The same holds for nervous system, which is detected thanks to serotonin+FMRFamide or serotonin+FMRFamide and acetylated tubulin.
32. FigS4: please show individual (not merged) staining and indicated regions with overlapping expression if any.
33. Fig S11: indicate the name of the clades presented in the tree.

<u>Reviewer 2</u>

Advance summary and potential significance to field

The authors provide gene expression profiles of multiple genes focusing on mesoderm development in brachipods and phoronids. By observing the effects of SU5402, they suggested that FGF signaling perform essential role in the mesoderm gene expressions and mesoderm development in the species of Spiralia.
Based on these results, the authors argue that the FGF signaling may have certain roles in mesoderm development of the common ancestors of bilaterians.

Comments for the author

The main concern I have in this manuscript is the weakness of the argument to suggest the role of FGF signaling in bilaterians. As the authors discuss, careful consideration is required to deduce the ancestral role of FGF signaling, and independent co-options is not highly unlikely. Many species develop mesoderm without the role of the FGF signaling, such as echinoderms. Mesoderm development is not something that FGF control everything, but rather more hierarchial process as the authors discuss in the manuscript. I wonder how to justify the argument of conserved role of FGF signaling in mesoderm development ignoring the differences of FGF roles among species (i.e., FGF for all mesodemr, or for part of the mesoderm). Considering these issues, I hesitate to interpret the results from two closely related taxa, brachipods and phoronids to represent the Spiralians.

Furthermore. I was not quite sure about the scientific impact of the fact that FGF signaling is involved in mesoderm formation in bilaterian ancestors. I cannot extend my curiosity from the conclusion, even though it will more confidently shown in the future studies. I like to see deeper

© 2021. Published by The Company of Biologists under the terms of the Creative Commons Attribution License (https://creativecommons.org/licenses/by/4.0/).

argument about the implications on the evolution of mesoderm, drawn from the conclusion that FGF has conserved (although I doubt it is well conserved) role in mesoderm development.

I also draw the attention of the authors about the specificity of the drug SU5402. It is widely used, but how do the authors examined whether any side effects do the drug have in the species examined here. Based on the expression patterns of FGF-related genes, it is safer to say that SU5402 did inhibit the function fo FGFR but the issue is how to exclude the possibility that the drug has side-effects. Perhaps the readers of DEVELOPMENT have strong concern on this issue.

Reviewer 3

Advance summary and potential significance to field

this is an interesting, and quite thoroughly documented manuscript on the potential for conserved mechansims of FGF-mediated mesoderm induction in diverse animals. It is certainly, in principle, appropriate for Development, but there are several shortcomings and interpretations that should be addressed. The overall message is not a radical but simple one (indeed if it had been we would be reading this in one of the weeklies), but is nevertheless an interesting set of observations to those interested in the evolution of Development. Many people assume that the core signaling pathways are conserved in function throughout animals, but the alternative view is that with a limited number of signaling pathways available, animals have coopted them in different ways. So this is useful even to make the point that there is no simple conserved function for signaling in development, even if the basal function of FGF remains obscure.

Comments for the author

We apologize for the slow review. By the time that the first reviewer realized that they were out of their depth, a more expert reviewer colleague had to be recruited, and this all took time, along with following through the rather dense presentation of the manuscript.
Nenetheless, this is an interesting, and quite thoroughly documented manuscript on the potential for conserved mechansims of FGF-mediated mesoderm induction in diverse animals. It is certainly, in principle appropriate for Development, but there are several shortcomings and interpretations that should be addressed. The overall message is not a radical but simple one (indeed if it had been we would be reading this in one of the weeklies), but is nevertheless an interesting set of observations to those interested in the evolution of Development. Many people assume that the core signaling pathways are conserved in function throughout animals, but the alternative view is that with a limited number of signaling pathways available animals have coopted them in different ways. So this is useful even to make the point that there is no simple conserved function for signaling in development, even if the basal function of FGF remains obscure.
1. the title emphasizes the Spiralia, but as is clear from figure 1. the animals tested are the Lophophorata.
Should this figure contain all of the groups that are referenced below? Or maybe have a more detailed phylogeny in the supplement. this point comes up explicitly in the discussion with the statement/confession
“To determine whether the involvement of FGF signaling in mesoderm formation was already present in the lastcommon ancestor of Bilateria, or whether it was independently co-opted in the lineage of lophophorates functional studies from more spiralian taxa are required.”
2. Many biologists would be surprised that deuterostomes generally form mesoderm by enterocoely. How widespread is this? Indeed, it seems to be a property of a fairly scattered selection of clades, both proto- and deuterostomes. Indeed, is this morphology section useful, since the morphological appearance of mesoderm is not necessarily relevant to the signaling mechanisms?
3. The more important issue of conservation of signaling is presented as a pretty solid phalanx of references without satisfactory discussion for the reader to interpret without doing a lot of extra reading. Perhaps with elimination of the morphology, this could be expanded to say something that would educate the reader. Or a more detailed phylogeny and references used could be put into the supplement as a figure with a summary of what pathways are used. In the context of this work, such a figure would be useful. For example which animals use Notch/FGF/BMP, and what about Nodal/Smad2? The list makes me feel a bad scholar, but instead of educating me, it puts me off reading further. 23 refs. How many are on signaling pathways? If they are mostly about the

© 2021. Published by The Company of Biologists under the terms of the Creative Commons Attribution License (https://creativecommons.org/licenses/by/4.0/).

transcription factors, then that is a separate topic and while it could be summarized in amore detailed figure, Gerhart Kirschner style. Here though the transcription factors are the tools, and while they should be justified, the emphasis in the text should be on the FGF signaling aspect.
4. page 3. “These animals exhibit “deuterostome-like” features in their development, such as radial cleavage and enterocoely (Zimmer, 1997).” If the mechanisms are examples of convergent evolution, then the more general conserrvation of mechanisms is something that could really only be addressed with an even more thorough survey of different clades. If morphological aspects of development are leading the way, then perhaps the signaling pathway follows? Its not as clear as it could be why these species were chosen. Of course availability is a strong motivator, and maybe that is enough.
5. There is something of a conflict stating that mesoderm in T. transversa- Could the authors clarify by what criterion it originates in the blastula, becuase it is hard for thes specified cells to be between the ectoderm and endoderm, so not mesoderm. The definition is presumably the twist expression, Do the authors wish to explicitly define twist expression as diagnostic for mesoderm?

6. Figure 1 presents the morphology of larvae. These are stained differently, but more importantly the pictures lack enough detail in labeling to be useful. (Anterior is at the top, but Ventral Dorsal, and is there Left right asymmetry?) Again this could be remedied by larger pictures in the supplement, where the relevant parts of mesoderm are also labeled. Indeed, one could hope that these confocal stacks could be submitted as rotating movies so that the newbie can get an idea of the 3D morphology.
7. We are confused about the use of T transversa. Could the authors clarify to what extent they are relying on results of Passamanek et al. versus showing their data? They say they are presenting results on T. transversa in the Results subtitle, but are these all Passamanek results?
8. twist, mox, six1/2 eya, mef2, dachs, paraxis, foxc, mprx, myod, limpet, foxf and nk1 Explain the rationale for these choices? Maybe a figure of a standard deuterostome and protostome decorated with gene expression patterns may give a rationale for these as conserved mesodermal markers. Or reference a good summary of thei conservation of expression in mesoderm. Indeed, the authors later point to the use of Mapkinase in molluscs, and so it is surprising that this stain was not used in the current analysis.
9. While the authors are to be commended for their extensive dataset, the panels are owerwhelming, and sometimes lack clear orientations, and would benefit from schematics . I would suggest that the authors choose the most illustrative examples, and put the backup data in the supplement, so that the panels can get more spacious treatment. They would also benefit from more labeling, so for example in the vegetal views which are dorsal and ventral sides? Is there a L/R difference?
10. “suggesting neuronal expression (Fig. 4B1). This expression persists in the gastrula and early larva stages
(Fig. 4B2-3) but in late larvae it disappears This is rathee vague. why is the early expression suspected to be neuronal. has there been independent staining to show neurons in the domain?
11. With respect to the FGF ligand and receptor analysis, the data presented could benefit from abit more digestion, since it seems particulalry that some regions are sensitive while others are not (eg “We saw a severe reduction of expression and detection in only one anterior coelomic sac”) . The text is pretty dense here, and maybe a table might summarize the results more clearly? By the time one has worked through the details, it is hard to remember much to see that the statement the staement “Overall, our data suggest an evident role of FGF signaling in mesoderm development in the lineage of lophophorates.” is justified or not.
Again a table, and in the text a brief sumamry of the most salient data (and a chocie of which genes to illustrate in the main text) would be useful. Could some of the changes be indirect cproblems from the early inhibitions?
12. What was the control for FGF inhibition by SU5402? Is sprouty a conserved response? Or was phospho MAP kinase tested?
13.
Minor points 1. The way mesoderm is formed differs between embryos. should be between species, or embryos of different species.
2. Spatiotemporal patterning of mesoderm. Could be expressed more precisely as Spatiotemproal expression of mesodermal markers during development?
3. “are only detected at the late gastrula stage” begin to be detected?
4. the earliest mesdermal marker is twist. Do the references cover the species being used here already, or is this a more general statement. As stated its hard to tell what is meant.

© 2021. Published by The Company of Biologists under the terms of the Creative Commons Attribution License (https://creativecommons.org/licenses/by/4.0/).

5. “Larvae are cleared from fgfr3 transcripts” fgfr3 is no longer deteced in larviae 6. expression “disappears” and odd choice of words perhaps fades, declines isnot presented 7. SU5402 was dissolved... it makes it sound as though the final stock conentrations were 5,
10 and 20 micromolar, but one assumes instead that there was a stock solution that was further diluted to these final concentraiton for application to embryos?

8. abbreviations such as SW and Ptx, PBT are used. Are these properly referenced elsewhere? I assume SW is artificail sea water?
9. procedure for fluorescent WMISH?
10. please add accession numbers

First revision

Author response to reviewers' comments

We thank the reviewers for their positive responses and helpful comments, which we addressed as following:

Reviewer comments:

Reviewer 1 Advance summary and potential significance to fieldIn their article entitled “FGF signalling induces mesoderm in members of Spiralia”, Carmen Andrikou and Andreas Hejnol describe the expression pattern of genes known as mesodermal/muscle markers in other bilaterian animals in one brachiopod and one phoronid species. They then assess the role of FGF signalling in the formation of the mesoderm in these species and in another brachiopod species by looking at the expression of the receptor and ligand genes during development, and by using pharmacological treatments with SU5402. FGF has been shown to be required at different steps of mesoderm or mesodermal derivatives formation in diverse bilaterian phyla (deuterostomes and ecdysozoans) and the work presented here brings potentially some indications suggesting that this signalling pathway would also play similar although variable roles in members of the Spiralia group. The authors present many data, most of them technically sounds. Although the question is extremely interesting and the use of non-classical models brings new insights into our understanding of mesoderm evolution in bilaterian, I have some major concerns detailed bellow.

Reviewer 1 Comments for the authorMajor global concerns:
1. All through the manuscript, there are some inaccuracies on the use of the term “mesoderm”. The mesoderm is a germ layer that during development form some specific structures among which muscles, but not only. The authors often use mesoderm to describe muscles, as for example in Figure 1: the actin immunostaining show the muscles and not the mesoderm that at the larval stage does not exist anymore.

Response: The reviewer is right. We corrected now the erroneously use of the word mesoderm throughout the manuscript, using it as a term only in the early development, whereas in the later stages we now refer specifically to mesoderm derivatives (e.g. muscles, coeloms).

2. Another concern is the use of the term induction. Induction of the mesoderm corresponds to the phenomenon through which cells (usually for a mesendodermal precursor) receive a signal, coming from other cells, that allows them to adopt a mesodermal fate. Further processes such as patterning, migration or differentiation are not considered as an induction phenomenon. Here the authors often inaccurately refer to the term induction. For example, in the abstract, the authors write “FGF signalling is involved in mesoderm induction in deuterostomes”: this is quite incorrect as it is not the case in many deuterostomes although FGF signal is implicated at different steps of mesoderm development in all deuterostomes. The whole manuscript should be corrected by using the precise and appropriate terms when speaking about mesoderm formation. This is crucial in order to really give the possibility to compare the role of signalling pathways in the different steps of this process between metazoan lineages.

© 2021. Published by The Company of Biologists under the terms of the Creative Commons Attribution License (https://creativecommons.org/licenses/by/4.0/).

Response: The reviewer is correct. We restricted the use of the word induction to the earliest stage of mesoderm formation and corrected the misleading use throughout the manuscript. For the later stages of mesoderm development appropriate terminology was used (e.g. migration, differentiation).

3. The use of GRN is also often inappropriate. GRN refers to genes for which a functional interaction has been demonstrated. In their work the authors describe transcription factor toolkit rather than GRN (for example page 4 end of the introduction).

Response: We agree with the reviewer. We tested the inappropriate use of GRN throughout the manuscript and eliminated it.

4. SU5402 is an inhibitor of the vertebrate FGFR1. It has been shown to inhibit the FGF signalling pathway in other deuterostomes through the use of other molecules that would impact this pathway at a different steps (for example MAPK inhibitors) or by showing that it is downregulating the expression of genes orthologous to known FGF signalling pathway targets in vertebrates. In their article, the authors do not test in any way the real potentiality of SU5402 to inhibit the FGF signalling pathway. It is also to note that the authors propose the species studied to have several FGF receptor genes (2 or 3 in the brachiopods). However, the phylogenetic tree presented (Supp Data) is not very well supported and fails to include sequences of receptors that were characterized as being closely related to FGFR and PDVEGFR families but that are neither members of FGFR nor PDVEGFR groups and that were called ExTK (see D'Aniello et al., 2008). The authors should be more careful in assigning the FGFR name to the RTK genes described here as from the phylogeny presented some FGFR might rather belong to this ExTK group. Overall, it seems hard from the presented data to support the fact that the phenotypes observed after treatment are due to the inhibition of the FGF pathway.

Response: We agree with the reviewer that the manuscript lacks direct evidence. We therefore tested the protein expression of phospho-MAPK -usually downstream of FGF signaling- in SU5042 treated embryos and found it downregulated. Moreover, we cloned and tested the expression of sprouty genes – conserved downstream modulators of FGF signaling- in treated embryos and also found it impaired. These results are now shown in Fig S10-11. However, due to the COVID outbreak, we couldn't conduct additional experiments with MAPK inhibitors. As stated in the materials and methods section, gravid animals are caught in the wild once per year during a short time window of gravidity and in different places (Friday harbor (USA), Monterey bay (USA), Bergen (Norway)) in the world. Therefore, due to the COVID outbreak we were not able to collect any animals last year (and this year either) in order to have access to living embryos and perform additional inhibitor experiments.
Concerning the FGFR phylogeny, the reviewer is absolutely right, and we thank him/her for pointing this out. We think that the problem was that we used sequences from several protostomes that probably were not well annotated as FGFR genes (maybe are also ExTK?) and our sequences seemed well nested within them. We now repeated our phylogenetic analysis using the ExTK sequences from the D'Aniello et al., 2008 paper and some well annotated FGFR sequences from protostomes (e.g. C.elegans and D.melanogaster) and N. vectensis. Indeed, 3 of our sequences (FGFR2 and FGFR3 of Terebratalia transversa, and FGFR3 of Novocrania anomala) group with sequences form the ExTK group. This means that only one sequence of T. transversa, FGFR1; two sequences of N. anomala, FGFR1 and FGFR2, and one of Ph. harmeri, FGFR1 are bona fide FGFR genes. Therefore, we provided now a new FGFR phylogenetic tree (S1), and changed Fig 4 and S5 accordingly, as well as the description in the text. The interpretation of the data doesn't change though, because the FGFR genes are mesodermally expressed in all three investigated species.

Specific comments on the text:
5. In the introduction the authors write: "deuterostomes generally form mesoderm by outpouchings of their invaginating endoderm (archenteron), a mechanism named enterocoely". Enterocoely refers to the formation of the coelom and not the mesoderm per se.

Response: We agree with the reviewer, this part was corrected as following: "Mesoderm can originate in an epithelial mode by outpouchings of the invaginating endoderm (for example in most deuterostomes (Franz, 1924; Hennig, 1984; Hyman, 1955; Hyman, 1959; Swalla, 1993) and two

© 2021. Published by The Company of Biologists under the terms of the Creative Commons Attribution License (https://creativecommons.org/licenses/by/4.0/).

clades of protostomes, the Chaetognatha (Hertwig, 1896; Kapp, 2000; Matus et al., 2006) and the Brachiopoda (Conklin, 1902; Kowalevsky, 1874; Plenk, 1913)), or in a mesenchymal mode from one or more precursor cells that internalize during gastrulation (for example in spiralian species where the source of mesoderm can be of endodermal (e.g. the micromere 4d) and ectodermal origin (e.g. micromeres from the animal pole/anterior end of the blastopore) (summarized in Henry and Martindale, 1999; Kozin and Kostyuchenko, 2016; Lambert, 2008; Lyons and Henry, 2014) and in ecdysozoans where mesoderm originates either from internalization of vegetal endomesodermal cells (Martin-Duran and Hejnol, 2015; Sulston et al., 1983), or cells of the blastoderm (Eriksson and Tait, 2012; Hartenstein et al., 1985))."

6. page 4 first line: replace "mesoderm" by "muscles".

Response: We replaced it according to the reviewer suggestion.

7. page 4 second line: 4 coelomic sacs are described for T. transversa, but it rather seems that they have only one, at least from Fig 1 and from all the other figures concerning this species.

Response: We now added in Fig 1 lateral views of all three species in order to show better the morphology of the larvae. Three out of four coelomic sacs with chaetae bundles are now better visible in T. transversa. We also provided lateral views of the animals in the remaining figures of the manuscript; we hope that now the morphology is more evident.

8. page 4 last sentence, the authors write "Despite our observed differences in the presumptive mesodermal gene regulatory networks (GRN), our results indicate a conservation of the inductive role of FGF signalling pathway in mesoderm with deuterostomes.". As I said before, here there is no analysis of GRN, but rather of TF toolkit, and the authors are not demonstrating a putative mesoderm inductive capacity at least for the brachiopods studied here for which SU5402 treatment alters mesoderm formation but not its induction if there is any.

Response: This sentence was changed according to the reviewer suggestion, as following: "Our results suggest an overall conserved involvement of FGF in mesoderm migration and differentiation in all three lophophorate species tested. Moreover, they show a similar mesoderm-inducing role of FGF in Ph. harmeri with members of deuterostomes."

9. page 5: I would rather say that "indicating that mesoderm is specified" rather than "indicating that mesoderm originates".

Response: We changed this sentence according to the reviewer suggestion.

10. page 6: "in N. anomala it is expressed in most of the mesoderm but acquires a stronger expression at the posterior one (cs4)": Replace by "in N. anomala it is expressed in most of the mesoderm but acquires a stronger expression in the posterior coelomic sac (cs4)"

Response: We changed this sentence as following: "in N. anomala it is expressed in three pairs of coelomic sacs (c2-c3-c4) but acquires a stronger expression in the most posterior one (cs4) (Fig. 2A6-7)"

11. page 6: "Dachs seems to be absent from the anterior mesodermal pattering in Ph. harmeri": rather replace by "Dachs is not expressed during anterior mesodermal patterning in Ph. harmeri".

Response: We changed this sentence as following: Dachs is not expressed in the anterior region in Ph. harmeri (Fig. 3F2-9)

12. page 6: "it mainly occupies anterior (apical) mesodermal fates": transcripts do not occupy a fate.

Response: The sentence is now modified according to the reviewer suggestion, as following: "Dachs is not expressed in the anterior region in Ph. harmeri (Fig. 3F2-9), while in T. transversa it

© 2021. Published by The Company of Biologists under the terms of the Creative Commons Attribution License (https://creativecommons.org/licenses/by/4.0/).

demarcates the entire musculature (Passamaneck et al., 2015), and in N. anomala it confines in the anterior pairs of coelomic sacs (c2-c3) (Fig. 2F6-7).

13. page 9: the expression of fgfr1 in anterior scattered cells for T. transversa is not visible and not indicated on the WISH pictures presented (Fig4A4).

Response: The reviewer is right, fgfr1 is indeed not expressed in anterior scattered cells but instead in two anterior-lateral ectodermal patches. We corrected our description as following: "In larvae, fgfr is additionally activated in two anterior-lateral ectodermal patches (Fig. 4A6-7)."

14. page 9: "The analysis of the spatial expression of the three receptors and the two ligands suggests a putative involvement of FGFR1 and FGF8/17/18 in mesoderm development (see co-expression of fgfr1 and fgf8/17/18 in Fig. S4)". I don't see any overlap of expression between fgfr1 and fgf8/17/18.

Response: We didn't mean that the expression overlaps, we were simply referring the co-expression analysis (double FISH) used in order to show the relative expression of the ligand and the receptor. Thus, to avoid confusion, we modified this sentence as: "(see relative expression of fgfr and fgf8/17/18 in Fig. S4).

15. page 10: it is not clear if "cs" corresponds to coelomic sac or chaete sacs. Please specify all through the manuscript.

Response: We apologize for the misunderstanding. Cs is referring to coelomic sacs and not to chaetae sacs but the fact that the first coelomic sac in N. anomala does not project in chaetae bundles led to confusion. We now corrected this thoughout the manuscript.

16. page 12: The effects observed for the treatment starting at the blastula stage in T. transversa suggest a failure of correct gastrulation movements rather than a failure in axial elongation as no blastopore is formed.

Response: In fact, it is difficult to completely separate the two processes. T. transversa is a protostome, meaning that the blastopore moves anteriorly in order to form the mouth. This movement of the blastopore happens during the axial elongation (Martin Duran et al 2016, Freeman 1993). In the treatments starting from the morula stage, the blastopore is not formed so gastrulation fails to occur. However, when starting at the blastula stage, the blastopore is formed but cannot move anteriorly, so the embryos are rested in this pre-axial elongation phase, suggesting that the axial elongation fails to occur. In order to test this hypothesis, we also conducted treatments starting from the gastrula stage in order to skip the beginning of gastrulation and see whether the embryos would be able to go through axial elongation. In fact, although in these embryos the blastopore has moved slightly more anteriorly compared to the ones treated from the blastula stage, they never managed to elongate as the control ones. Finally, the fact that the expression of the anterior markers was unaltered while the mesodermal posterior expression was impaired further indicates that -since the axial elongation takes place from anterior to posterior in this species- the failure in axial elongation is probably coupled with a failure in mesoderm migration and differentiation.

17. page 16: "To test whether the role of FGF signalling in mesoderm induction extends beyond the brachiopod lineage..": the authors did not show a failure in mesoderm induction after SU5402 treatment although they show it affects mesoderm and mesodermal structures development.

Response: We agree with the reviewer. We corrected this sentence as: "To test the role of FGF signaling in mesoderm development beyond the brachiopod lineage, we also treated embryos of another lophophorate species, Ph. harmeri with SU5402 at different developmental stages (for a summary of treatments see Figs S6 and S7)."

18. page 16: "To test wether this was due": please explain to what "this"refers.

Response: We changed this sentence according to the reviewer suggestion: "To test whether the observed mesoderm malformation was due to a failure in mesoderm induction, we also tested the

© 2021. Published by The Company of Biologists under the terms of the Creative Commons Attribution License (https://creativecommons.org/licenses/by/4.0/).

expression of the earliest mesodermal markers twist and six3/6 in gastrulae treated from the blastula stage on, and found it abolished (Fig. S9).”

19. page 16: “These results show that FGF signalling is upstream of anteroposterior patterning”: I don’t see which result shows this implication. It is not because the expression of two genes (normally expressed anteriorly in the mouth or posteriorly) is abolished that the A/P patterning is affected. The expression of A/P patterning genes should be tested to answer this point.

Response: If the reviewer refers to Hox genes as possible A/P markers, the only one that could be tested is pb/hox2 cause the others are not expressed yet in the larva stage of Ph. harmeri (Gąsiorowski and Hejnol 2020). Moreover, they are not ideal genes for testing A/P patterning in this animal, since they are all -even the so called “anterior hox” genes- expressed posteriorly (Gąsiorowski and Hejnol 2020). Therefore, we tested now the expression of pb/hox2 and also of cdx in the treated embryos. These results are now included in Fig 6. In total we have tested 3 genes pattering anterior regions (otx, nk2.1, six3/6) and 4 genes for posterior fates (nk2.1, cdx, foxc and pb/hox2). The expression of otx and six3/6 -markers of the apical organ- and most of the posterior genes (nk2.1, foxc, pb/hox) is downregulated in the treated embryos, suggesting that the A/P patterning is somehow impaired.

20. page 20: second paragraph: I think comparing FGFR1 and FGF8/17/18 potentially conserved role in posterior elongation between vertebrates and brachiopods, from the data presented, is taking a shortcut that should not be included in the discussion. It is clear that even among ecdysozoans, and among deuterostomes, “posterior elongation” has different meanings and implies different biological processes. Moreover the authors write: “The role of FGF signalling in the posterior axial elongation of T. transversa resembles mechanisms typically deployed by a number of deuterostomes in order to coordinate embryonic patterning and elongation of posterior tissues: in the tunicate species Ciona intestinalis, FGF, together with the canonical WNT and Retinoic Acid (RA) signalling, are establishing the posterior patterning of the tail (Pasini et al., 2012), in amphioxus FGFand RA are coordinating the posterior elongation (Bertrand et al., 2015)” and are therefore mixing posterior growth and patterning of posterior tissues. In ascidians, what has been shown in Pasini et al. is not associated to posterior elongation. In cephalochordates, RA and FGF are not controlling posterior elongation in contrary to what is known in vertebrates.

Response: We have now eliminated the comparative part of tunicates and cephalochordates and only refer to the vertebrate situation, where FGF is involved in posterior elongation and mesoderm differentiation: “Another outcome of this study concerns the evident relationship witnessed between mesoderm development and posterior axis elongation in T. transversa. The expression of fgf8/11/18 mRNA in the growing posterior tip of the embryo in relation to fgfr1 (Fig. S4), suggests that FGF8/11/18 might act as a gradient that progressively coordinates the posterior axial elongation of the embryo and mesoderm differentiation, similar to what has been described in vertebrates (Dubrulle and Pourquie, 2004)”.

21. p20-p21: “A coordinated interplay of WNT and FGF signalling in regulating posterior axial elongation seems also likely, suggesting a putative conserved developmental signalling mechanism in orchestrating posterior regionalization. “. To my opinion there are no data presented in the manuscript that could support such proposition.

Response: The reviewer is correct as this sentence was meant to be only speculative. We have now eliminated it from the text.

22. p21: please explain what is “presursor 3D”

Response: We changed this sentence according to the reviewer suggestion: “Moreover, a putative conserved FGF signaling event on mesoderm induction is possibly also present in bryozoans, as suggested from the activation of the MAPK pathway in the endomesodermal precursor cell (3D blastomere) (Vellutini et al., 2017), which would imply that the inductive role of FGF signaling in mesoderm formation could have been present in the last common ancestor of lophophorates.”

© 2021. Published by The Company of Biologists under the terms of the Creative Commons Attribution License (https://creativecommons.org/licenses/by/4.0/).

23. p22: for phylogenetic reconstructions, it is indicated that ML inference was used in the material and methods section whereas in the FigS1 legend the authors indicate they used Bayesian inference. Given the numbers indicated as supports in the trees, it might have been a ML analysis. Please clarify this point.

Response: The reviewer is right. We changed this sentence in the Material and Methods part, accordingly.

24. p22: SU5402 treatment: I believe SU5402 was first dissolve in DMSO at a high concentration to prepare a stock solution and then dissolved in sea water at the indicated concentrations.

Response: The reviewer is correct. We changed this sentence as following: "SU5402 was dissolved in DMSO to a stock solution of 10 mM and then serially diluted in the concentrations of 5 um, 10 uM and 20 μM in seawater."

25. p22: please specify what PBT and PBX mean.

Response: We changed this sentence as following: "Embryos were permeabilized in 100% Methanol for 1 hour, digested with Proteinase K (10 μg ml−1) for 5 minutes, fixed in 4% paraformaldehyde in sea water for 30 minutes, washed for 3 hours in phosphate buffer saline containing 0,5% Triton (PTX), washed once in phosphate buffer containing 0,1% Tween-20 (PBT) for 5 minutes and incubated in 4% sheep serum in PBT for 30 min."

26. p22-p23: please indicate the dilution used for the antibodies.

Response: We now added all the dilutions used for the antibodies.

Figure comments:
27. In most of the figures some information are missing: scale bars, full orientation of the embryos/larva (dorsal part for lateral views?). Moreover, for larva, I imagine that vv does not mean vegetal view but rather ventral view.

Response: The reviewer is right. We have now included scale bars in all fluorescent images and provided full orientation of all early embryos (vegetal views and lateral views) and larvae (dorsoventral and lateral views).

28. Some inserts are not visible (too small).

Response: The reviewer is correct. We increased the size of the insets.

29. Inserts in Fig.4, corresponding to fluorescent in situ hybridization are very small. Moreover, after zooming they do not show the same results as with the chromogenic in situ hybridization: this should be explained and commented, or chromogenic in situ hybridization should be undertaken again.

Response: The reviewer is correct; the inset was indeed too small. However, it both cases, fluorescent and colorimetric, the signal was demarcating endodermal and mesodermal cells delaminating from the archenteron in both receptors (and some additional ectodermal staining in the case of fgfr1). Nevertheless, due to the data density of the figure and in order to have consistency in our panels, we decided to eliminate the fluorescent images.

30. In almost all the figures, SU5402 is written SU502.

Response: The reviewer is right, we corrected it.

31. It is not clear why muscles are detected thanks to actin, tropomyosin or HB9 immunostaining (for example between Fig. 5 and Fig. S8). The same holds for nervous system, which is detected thanks to serotonin+FMRFamide or serotonin+FMRFamide and acetylated tubulin.

© 2021. Published by The Company of Biologists under the terms of the Creative Commons Attribution License (https://creativecommons.org/licenses/by/4.0/).

Response: HB9 staining was shown elsewhere (Vellutini et al 2016) to label musculature in T. transversa and therefore we decided to use it as a diagnostic marker. However, it is true that having two independent stainings for the same purpose can be rather confusing and so we eliminated HB9 and only used actin for labeling musculature in order to keep it consistent. In the same logic, for staining the nervous system we removed the acetylated tubulin and used only the serotonin+FMRFamide stainings.

32. FigS4: please show individual (not merged) staining and indicated regions with overlapping expression if any.

Response: Individual stainings are now provided in Fig S4.

33. Fig S11: indicate the name of the clades presented in the tree.

Response: The clade names are now provided in Fig S12 (previously S11).

Reviewer 2 Advance summary and potential significance to field
The authors provide gene expression profiles of multiple genes focusing on mesoderm development in brachipods and phoronids. By observing the effects of SU5402, they suggested that FGF signaling perform essential role in the mesoderm gene expressions and mesoderm development in the species of Spiralia. Based on these results, the authors argue that the FGF signaling may have certain roles in mesoderm development of the common ancestors of bilaterians.

Reviewer 2 Comments for the author
The main concern I have in this manuscript is the weakness of the argument to suggest the role of FGF signaling in bilaterians. As the authors discuss, careful consideration is required to deduce the ancestral role of FGF signaling, and independent co-options is not highly unlikely. Many species develop mesoderm without the role of the FGF signaling, such as echinoderms. Mesoderm development is not something that FGF control everything, but rather more hierarchial process as the authors discuss in the manuscript. I wonder how to justify the argument of conserved role of FGF signaling in mesoderm development ignoring the differences of FGF roles among species (i.e., FGF for all mesodemr, or for part of the mesoderm). Considering these issues, I hesitate to interpret the results from two closely related taxa, brachipods and phoronids to represent the Spiralians. Furthermore. I was not quite sure about the scientific impact of the fact that FGF signaling is involved in mesoderm formation in bilaterian ancestors. I cannot extend my curiosity from the conclusion, even though it will more confidently shown in the future studies. I like to see deeper argument about the implications on the evolution of mesoderm, drawn from the conclusion that FGF has conserved (although I doubt it is well conserved) role in mesoderm development. I also draw the attention of the authors about the specificity of the drug SU5402. It is widely used, but how do the authors examined whether any side effects do the drug have in the species examined here. Based on the expression patterns of FGF-related genes, it is safer to say that SU5402 did inhibit the function fo FGFR, but the issue is how to exclude the possibility that the drug has side-effects. Perhaps the readers of DEVELOPMENT have strong concern on this issue.

Response: The reviewer raised two important concerns.
Firstly, on the ancestral role of FGF signaling on mesoderm induction and its implications on mesoderm evolution. The reviewer is correct, it was indeed never our intention to suggest an ancestry of the inductive role of FGF signaling in mesoderm, we were simply highlighting the repetitive use of FGF on mesoderm induction in another group outside deuterostomes. Indeed, what we see in lophophorates might simply be a product of cooption. One cannot be sure unless more spiralian species are tested, as we say in the discussion. Parts where the ancestral role of FGF signaling on mesoderm induction could have been unintentionally implied, have now been modified (e.g. the last sentence of the abstract: “Our findings suggest that the mesoderm-inducing role of FGF extends the group of deuterostomes” instead of “Our findings suggest that the mesoderm-inducing role of FGF predates the origin of deuterostomes”).
Secondly, on the specificity of SU5402 on FGF signaling. To address the specificity of SU5402 we tested the protein expression of phospho-MAPK (downstream of FGF signaling) in fixed SU5042 treated embryos and found it impaired, suggesting that MAPK signaling is affected. Moreover, we cloned and tested the expression of sprouty genes – conserved downstream modulators of FGF signaling- and also found it impaired. These results are now shown in Fig S10-11. However, due to

© 2021. Published by The Company of Biologists under the terms of the Creative Commons Attribution License (https://creativecommons.org/licenses/by/4.0/).

the COVID outbreak, we unfortunately were not able to collect any animals and access to living embryos and therefore we couldn't conduct additional experiments with more inhibitors (e.g. inhibiting MAPK and supposing that MAPK is downstream of FGF signaling) as gravid animals are caught in the wild once per year during a short time window of gravidity and in different places (Friday harbor (USA), Monterey bay (USA), Bergen (Norway)) in the world.

Reviewer 3 Advance summary and potential significance to field
This is an interesting, and quite thoroughly documented manuscript on the potential for conserved mechansims of FGF-mediated mesoderm induction in diverse animals. It is certainly, in principle, appropriate for Development, but there are several shortcomings and interpretations that should be addressed. The overall message is not a radical but simple one (indeed if it had been we would be reading this in one of the weeklies), but is nevertheless an interesting set of observations to those interested in the evolution of Development. Many people assume that the core signaling pathways are conserved in function throughout animals, but the alternative view is that with a limited number of signaling pathways available, animals have coopted them in different ways. So this is useful even to make the point that there is no simple conserved function for signaling in development, even if the basal function of FGF remains obscure.

Reviewer 3 Comments for the author

We apologize for the slow review. By the time that the first reviewer realized that they were out of their depth, a more expert reviewer colleague had to be recruited, and this all took time, along with following through the rather dense presentation of the manuscript.

1. the title emphasizes the Spiralia, but as is clear from figure 1. the animals tested are the Lophophorata. Should this figure contain all of the groups that are referenced below? Or maybe have a more detailed phylogeny in the supplement. this point comes up explicitly in the discussion with the statement/confession "To determine whether the involvement of FGF signaling in mesoderm formation was already present in the lastcommon ancestor of Bilateria, or whether it was independently co-opted in the lineage of lophophorates, functional studies from more spiralian taxa are required."

Response: We added the names of all the groups that are refenced in the introduction in Fig. S12, where we already have a detailed phylogeny with the role of FGF signaling in bilaterian species.

2. Many biologists would be surprised that deuterostomes generally form mesoderm by enterocoely. How widespread is this? Indeed, it seems to be a property of a fairly scattered selection of clades, both proto- and deuterostomes. Indeed, is this morphology section useful, since the morphological appearance of mesoderm is not necessarily relevant to the signaling mechanisms?

Response: We agree with the reviewer, mesoderm formation by enterocoely is a property of a selection of protostome clades (Chaetognatha and Brachiopoda), which are mentioned in the introduction and some deuterostomes. Indeed, not all mesodermal populations are formed by enterocoely in deuterostomes (e.g. head mesoderm in vertebrates), so in order to avoid confusion this part was re-written as following: "The way mesoderm is formed varies between embryos of different species. Mesoderm can originate in an epithelial mode by outpouchings of the invaginating endoderm (for example in most deuterostomes (Franz, 1924; Hennig, 1984; Hyman, 1955; Hyman, 1959; Swalla, 1993) and two clades of protostomes, the Chaetognatha (Hertwig, 1896; Kapp, 2000; Matus et al., 2006) and the Brachiopoda (Conklin, 1902; Kowalevsky, 1874; Plenk, 1913)), or in a mesenchymal mode from one or more precursor cells that internalize during gastrulation (for example in spiralian species where the source of mesoderm can be of endodermal (e.g. the micromere 4d) and ectodermal origin (e.g. micromeres from the animal pole/anterior end of the blastopore) (summarized in Henry and Martindale, 1999; Kozin and Kostyuchenko, 2016; Lambert, 2008; Lyons and Henry, 2014) and in ecdysozoans where mesoderm originates either from internalization of vegetal endomesodermal cells (Martin-Duran and Hejnol, 2015; Sulston et al., 1983), or cells of the blastoderm (Eriksson and Tait, 2012; Hartenstein et al., 1985))."
We believe the morphological section is helpful for the reader in order to get familiar with the differences in mesoderm formation between animals, but also to see that our species of interest (lophophorates) form mesoderm in a similar way to some deuterostomes (e.g. hemichordates).

© 2021. Published by The Company of Biologists under the terms of the Creative Commons Attribution License (https://creativecommons.org/licenses/by/4.0/).

3. The more important issue of conservation of signaling is presented as a pretty solid phalanx of references, without satisfactory discussion for the reader to interpret without doing a lot of extra reading. Perhaps with elimination of the morphology, this could be expanded to say something that would educate the reader. Or a more detailed phylogeny and references used could be put into the supplement as a figure with a summary of what pathways are used. In the context of this work, such a figure would be useful. For example which animals use Notch/FGF/BMP, and what about Nodal/Smad2? The list makes me feel a bad scholar, but instead of educating me, it puts me off reading further. 23 refs. How many are on signaling pathways? If they are mostly about the transcription factors, then that is a separate topic and while it could be summarized in amore detailed figure, Gerhart Kirschner style. Here though the transcription factors are the tools, and while they should be justified, the emphasis in the text should be on the FGF signaling aspect.

Response: The reviewer is right. We now added a table S1 with a summary of expression of the mesodermal transcription factors in bilaterians as well as the mesoderm-inducing pathways used by different species together with their references. The reader can now place the table next to the manuscript and be up to date.

4. page 3. “These animals exhibit “deuterostome-like” features in their development, such as radial cleavage and enterocoely (Zimmer, 1997).” If the mechanisms are examples of convergent evolution, then the more general conserrvation of mechanisms is something that could really only be addressed with an even more thorough survey of different clades. If morphological aspects of development are leading the way, then perhaps the signaling pathway follows? Its not as clear as it could be why these species were chosen. Of course availability is a strong motivator, and maybe that is enough.

Response: We apologize if the species choice was not clear. We chose these species for a number of reasons. First and most importantly we wanted to broad the taxon sampling by testing a clade within Spiralia, of which not only we had access to embryos and working techniques, but also previous knowledge about the development. These embryos exhibit enterocoely, which makes it easy to compare with deuterostomes where the role of FGF in mesoderm induction has been demonstrated (e.g. Green et al 2013). Secondly, we wanted to test closely related species that develop differently not only in their gastrulation mode (deuterostomy vs protostomy), but also in their way they develop mesoderm (origin, compartmentalization, differentiation) in order to conduct our evolutionary comparisons.

5. There is something of a conflict stating that mesoderm in T. transversa- Could the authors clarify by what criterion it originates in the blastula, becuase it is hard for thes specified cells to be between the ectoderm and endoderm, so not mesoderm. The definition is presumably the twist expression, Do the authors wish to explicitly define twist expression as diagnostic for mesoderm?

Response: We understand the concern of the reviewer. Although mesoderm cannot morphologically be discriminated yet, the expression of this mesodermal marker suggests that the mesodermal cells are already specified as early as the blastula stage and start to migrate between ectoderm and endoderm few hours later, at the early gastrula stage. Mesoderm appearance and patterning in T. transversa has been already described in previous papers (Martín-Durán et al., 2016; Passamaneck et al., 2015) and their diagnostic marker was twist, where they show that mesoderm is specified already at the blastula stage.

6. Figure 1 presents the morphology of larvae. These are stained differently, but more importantly the pictures lack enough detail in labeling to be useful. (Anterior is at the top, but Ventral Dorsal, and is there Left right asymmetry?) Again this could be remedied by larger pictures in the supplement, where the relevant parts of mesoderm are also labeled. Indeed, one could hope that these confocal stacks could be submitted as rotating movies so that the newbie can get an idea of the 3D morphology.

Response: The reviewer is right. We have now added in Fig. 1 a lateral view of all three species and added labelling (dorsoventral, anteroposterior) for better orientation and understanding of the morphology. We didn’t add L/R as there is no left right asymmetry seen in these embryos (at least in the investigated developmental stages). Moreover, we generated a figure (Fig. S7), where we provide 3D reconstructions of all three species.

© 2021. Published by The Company of Biologists under the terms of the Creative Commons Attribution License (https://creativecommons.org/licenses/by/4.0/).

7. We are confused about the use of T transversa. Could the authors clarify to what extent they are relying on results of Passamanek et al. versus showing their data? They say they are presenting results on T. transversa, in the Results subtitle, but are these all Passamanek results?

Response: We are sorry if this was not clear enough. The data on mesodermal gene expression during the development of T. transversa are from Passamaneck et al paper, but the expression analysis of the FGF components as well as the inhibitor experiments belong to our study.

8. twist, mox, six1/2 eya, mef2, dachs, paraxis, foxc, mprx, myod, limpet, foxf and nk1 Explain the rationale for these choices? Maybe a figure of a standard deuterostome and protostome decorated with gene expression patterns may give a rationale for these as conserved mesodermal markers. Or reference a good summary of thei conservation of expression in mesoderm. Indeed, the authors later point to the use of Mapkinase in molluscs, and so it is surprising that this stain was not used in the current analysis.

Response: We now added a table S1 with a summary of expression of all these mesodermal transcription factors in bilaterians. As for the MAPK expression, in order to address the specificity of the SU5402 inhibitor we tested the phospho-MAPK protein localization in control and treated embryos and found it downregulated compared to the controls. These data are now presented in Fig. S10.

9. While the authors are to be commended for their extensive dataset, the panels are owerwhelming, and sometimes lack clear orientations, and would benefit from schematics. I would suggest that the authors choose the most illustrative examples, and put the backup data in the supplement, so that the panels can get more spacious treatment. They would also benefit from more labeling, so for example in the vegetal views, which are dorsal and ventral sides? Is there a L/R difference?

Response: The reviewer is right; we have labeled better the panels in the main figures and added dorsoventral and lateral views, to ensure a better understanding of the embryo's orientation and morphology. We also generated a figure (Fig S7), where we provide 3D reconstructions of all control and treated embryos as well as a summary with the resulting phenotypes. There was no notable L/R difference least in the developmental stages investigated.

10. "suggesting neuronal expression (Fig. 4B1). This expression persists in the gastrula and early larva stages (Fig. 4B2-3) but in late larvae it disappears This is rathee vague. why is the early expression suspected to be neuronal. has there been independent staining to show neurons in the domain?

Response: We agree with the reviewer, indeed we didn't show independent stainings to confirm that this is neuronal expression. However, during the revision process we realized that fgfr2 (and two more genes) was not a bona fide FGFR sequence -as pointed out from reviewer 1- but instead it groups with another Tyrosine Kinase group, the so called ExTK (D'Aniello et al., 2008). Therefore, we provided a new phylogeny, eliminated the description of fgfr2 from the text (and of the other two genes as well) and modified Figs 4 and S5.

11. With respect to the FGF ligand and receptor analysis, the data presented could benefit from abit more digestion, since it seems particulalry that some regions are sensitive while others are not (eg "We saw a severe reduction of expression and detection in only one anterior coelomic sac"). The text is pretty dense here, and maybe a table might summarize the results more clearly? By the time one has worked through the details, it is hard to remember much to see that the statement the staement "Overall, our data suggest an evident role of FGF signaling in mesoderm development in the lineage of lophophorates." is justified or not. Again a table, and in the text a brief sumamry of the most salient data (and a chocie of which genes to illustrate in the main text) would be useful. Could some of the changes be indirect cproblems from the early inhibitions?

Response: We now added a new figure with a detailed summary of the phenotypes observed due to the inhibitor experiments (Fig. S7). We also provide summary sentences with the key findings in each animal in the Result section.

© 2021. Published by The Company of Biologists under the terms of the Creative Commons Attribution License (https://creativecommons.org/licenses/by/4.0/).

12. What was the control for FGF inhibition by SU5402? Is sprouty a conserved response? Or was phospho MAP kinase tested?

Response: We now included further evidence of FGF inhibition by SU5402, by testing both sprouty gene expression and phosphor-MAPK protein expression in the treated embryos and found them both impaired. These results are now presented in Fig S10-11.

Minor points:
1. The way mesoderm is formed differs between embryos. should be between species, or embryos of different species.

Response: We changed this sentence according to the reviewer suggestion: "The way mesoderm is formed varies between embryos of different species."

2. Spatiotemporal patterning of mesoderm. Could be expressed more precisely as Spatiotemproal expression of mesodermal markers during development?

Response: We changed the title of our results section according to the reviewer suggestion to "The spatiotemporal expression of mesodermal markers during development differs between N. anomala, T. transversa and Ph. harmeri"

3. "are only detected at the late gastrula stage" begin to be detected?

Response: We changed this sentence according to the reviewer suggestion.

4. the earliest mesdermal marker is twist. Do the references cover the species being used here already, or is this a more general statement. As stated its hard to tell what is meant.

Response: The references being used cover the Ph. harmeri and T. transversa species. To avoid confusion we moved the reference a bit further down in the sentence which is now as following: The earliest mesodermal marker is twist, whose expression initiates at the early gastrula stage and demarcates the entire mesoderm in both species (Figs 2A2-3 and 3A2-3) (Andrikou et al., 2019; Martín-Durán et al., 2016), indicating that mesoderm is specified before its morphological separation from endoderm."

5. "Larvae are cleared from fgfr3 transcripts" fgfr3 is no longer deteced in larviae

Response: We eliminated the sentence as fgfr3 was not a bona fide FGFR sequence.

6. expression "disappears" and odd choice of words perhaps fades, declines isnot presented

Response: We changed the word "disappears" to "fades", according to the reviewer suggestion.

7. SU5402 was dissolved... it makes it sound as though the final stock conentrations were 5, 10 and 20 micromolar, but one assumes instead that there was a stock solution that was further diluted to these final concentraiton for application to embryos?

Response: The reviewer is correct. We changed this sentence as following: "SU5402 was dissolved in DMSO to a stock solution of 10 mM and then serially diluted in the concentrations of 5 um, 10 uM and 20 µM in seawater."

8. abbreviations such as SW and Ptx, PBT are used. Are these properly referenced elsewhere? I assume SW is artificail sea water?

Response: We changed this sentence according to the reviewer suggestion: "Embryos were permeabilized in 100% Methanol for 1 hour, digested with Proteinase K (10 µg ml−1) for 5 minutes, fixed in 4% paraformaldehyde in sea water for 30 minutes, washed for 3 hours in phosphate buffer saline containing 0,5% Triton (PTX), washed once in phosphate buffer containing 0,1% Tween-20

© 2021. Published by The Company of Biologists under the terms of the Creative Commons Attribution License (https://creativecommons.org/licenses/by/4.0/).

(PBT) for 5 minutes and incubated in 4% sheep serum in PBT for 30 min.” Also, we changed “SW” to “filtered sea water”.

9. procedure for fluorescent WMISH?

Response: The procedure for both fluorescent WMISH is referenced in (Andrikou et al., 2019; Martín-Durán et al., 2016; Santagata et al., 2012). We now changed the sentence as following: “Embryos were manually collected, fixed in 4% paraformaldehyde in filtered sea water for 60 minutes, permeabilized in 100% Methanol overnight and processed for colorimetric and double fluorescent in situ hybridization as described in (Andrikou et al., 2019; Martín-Durán et al., 2016; Santagata et al., 2012).”

10. please add accession numbers

Response: The accession numbers have now been added in the text (Data availability).

Second decision letter

MS ID#: DEVELOP/2020/196089

MS TITLE: FGF signaling induces mesoderm in members of Spiralia

AUTHORS: Carmen Andrikou and Andreas Hejnol

I have now received all the referees reports on the above manuscript, and have reached a decision. The referees' comments are appended below, or you can access them online: please go to BenchPress and click on the 'Manuscripts with Decisions' queue in the Author Area.

The overall evaluation is positive and we would like to publish a revised manuscript in Development, provided that the referees' comments can be satisfactorily addressed. As you will see, the referees' comments can mostly be addressed with clarifications and changes to the text. I tend to agree with Referee 1 that the phosphoErk1/2 data is less convincing than the Sprouty1/2 in situ hybridisation and you might consider removing the phosphoErk data from the manuscript. I also think that describing these data before the phenotype of SU5402-treated embryos makes logical sense, however, this is your decision.Please attend to all of the reviewers' comments in your revised manuscript and detail them in your point-by-point response. If you do not agree with any of their criticisms or suggestions explain clearly why this is so.

We are aware that you may currently be unable to access the lab to undertake experimental revisions. If it would be helpful, we encourage you to contact us to discuss your revision in greater detail. Please send us a point-by-point response indicating where you are able to address concerns raised (either experimentally or by changes to the text) and where you will not be able to do so within the normal timeframe of a revision. We will then provide further guidance. Please also note that we are happy to extend revision timeframes as necessary.

Reviewer 1

Advance summary and potential significance to field

As previously said,in their article entitled “FGF signalling induces mesoderm in members of Spiralia”, Carmen Andrikou and Andreas Hejnol describe the expression pattern of genes known as mesodermal/muscle markers in other bilaterian animals in one brachiopod and one phoronid species. They then assess the role of FGF signalling in the formation of the mesoderm in these species and in another brachiopod species by looking at the expression of the receptor and ligand genes during development, and by using pharmacological treatments with SU5402. FGF has been

© 2021. Published by The Company of Biologists under the terms of the Creative Commons Attribution License (https://creativecommons.org/licenses/by/4.0/).

shown to be required at different steps of mesoderm or mesodermal derivatives formation in diverse bilaterian phyla (deuterostomes and ecdysozoans) and the work presented here brings potentially some indications suggesting that this signalling pathway would also play similar although variable roles in members of the Spiralia group. The authors present many data, most of them technically sounds. The authors have answered most of my comments and have made a big effort by undertaking new experiments in order to test for the potential specificity of SU5402. However, I still have some concerns detailed below.

Comments for the author

1. The anti-dpERK1/2 immunostaining was undertaken only on two species over three. In the corresponding FigS10, the authors indicate that the pictures correspond to larva, but I believe they rather correspond to gastrula stage embryos. I feel the results are not really convincing. Although the nuclear signal is lost in treated embryos, the few nuclei labelled in control embryos do not seem to correspond to cells expressing FGFR. I know that immunostaining against dpERK1/2 is not efficient in many species and I would not present these results. The data on Sprouty genes expression is much more convincing to me, with the loss of expression in treated embryos observed early in N. anomala and Ph. harmeri, and later in T. transversa. Therefore, I think these results should be presented as a main figure, and before the analysis of the SU5402-treated embryos phenotype, not as the last results section (so before the “Perturbation of FGF signalling...” section).
2. Concerning MAPK, in the main text and the figure legends, the authors are not analysing the expression of MAPK (MAPK is a big family with many members), they are analysing the translocation into the nucleus of the active form (diphosphorylated) of ERK1/2 which is described as the downstream effector of the MAPK pathway activated by FGFR.
3. FigS7 is absolutely not informative 4. In all the figures the insets are extremely small and sometimes covering the main picture. Moreover, panel labels are also often overlapping with the embryo, which renders the nice results very hard to see even at 300% zoom. This should really be modified.
5. Title: again, I see a problem with the use of “induction” in the title 6. Introduction page 3: “in T. transversa mesoderm originates... » I believe the mesoderm does not originate but is specified at the blastula stage.
7. Results, page 5, twice : “(c2-c3-c4)”: I believe it is rather” (cs2-cs3-cs4)”. Same think page 6 8. Results, page 11: “Moreover, an additional participation of FGF in mesoderm migration...”: I do not understand how, only based on coexpression of fgfr and twist, a function of FGF signal in mesoderm migration can be speculated.
9. Discussion section, page 15: “impaired gastrulation can occur either as a direct...”: impaired gastrulation is proposed to be due to impaired mesoderm formation. However, it is rather impaired mesoderm formation that results from gastrulation defects/blocking. I think the results should be discussed in this sense.
10. Discussion section page 15 : “The expression of fgf8/11/18”: it is “fgf8/17/18”. Moreover, there are absolutely no evidence for a gradient and the sentence is really too speculative.
11. Discussion section, page 16: MAPK, as I said before, corresponds in a fact to a multiplicity of pathways and precisions are needed. Moreover, MAPK pathways are activated by a wide range of signals, among which many RTKs (Receptor Tyrosine Kinase) that are not FGFRs, and bilaterians all possess many RTKs that might be using MAPK as a cytoplasmic output of their activation. Hence, the last sentence is a real shortcut that should be avoided.
12. Materials and methods: for double fluorescent ISH, were only DIG-labelled probes used?
13. “dv” is used for dorsoventral view. But I believe it is or dorsal or ventral views. It should be specified more clearly in all the figures.
14. I am not a native English speaker, but I think a little bit of editing is required.
15. uM or µM are used 16. Please add line numbers

Reviewer 2

Advance summary and potential significance to field

It was my misunderstanding that the author suggest conserved role of FGF signaling in the bilaterians.

© 2021. Published by The Company of Biologists under the terms of the Creative Commons Attribution License (https://creativecommons.org/licenses/by/4.0/).

Comments for the author

My comments were properly addressed in the revised manuscript. I like their additional experiment on the specificity of SU5402.

Reviewer 3

Advance summary and potential significance to field

As before

Comments for the author

Minor revisions:
Abstract

1. "FGF signaling is involved in mesoderm induction in members of deuterostomes (e.g. tunicates hemichordates) [...]": I think that other examples could be better for defining Deuterostomia.
2. "Moreover, inhibitor experiments demonstrate that FGF [...]": I suggest changing the name of the approach i.e "the use of the inhibitor X".
Introduction
1. "Mesoderm is an embryonic germ layer of bilaterians that gives rise to tissues residing between the ectoderm and endoderm, such as coeloms and muscles (Hyman, 1951; Ruppert, 1991). The way mesoderm is formed varies between embryos of different species. Mesoderm can originate in an epithelial mode by outpouchings of the invaginating endoderm (for example in most deuterostomes (Franz, 1924; Hennig, 1984; Hyman, 1955; Hyman, 1959; Swalla, 1993) and two clades of protostomes, the Chaetognatha (Hertwig, 1896; Kapp, 2000; Matus et al., 2006) and the Brachiopoda (Conklin, 1902; Kowalevsky, 1874; Plenk, 1913)), or in a mesenchymal mode from one or more precursor cells that internalize during gastrulation (for example in spiralian species, [...]": Coeloms are not tissue but cavities, generated by the gastrulation process, but not by mesoderm itself. On the other hand, "epithelial and mesenchymal" are not defined types of gastrulation, as invagination and delamination are. I think that a more proper term is required here.
2. "This molecular conservation has been commonly used as an argument for the homology of this germ layer [...]": This paragraph didn't mention molecules. I consider "mechanisms" to be more accurate.
3. "TVC and tail muscle in tunicates [...]": This abbreviation hasn't been defined before 4.
"These data show that in all three organisms, mesoderm development exhibit differences not only in the recruitment of regulatory genes but also in their temporal and spatial expression profiles, suggesting diverse underlying patterning mechanisms [...]": I suggest a review of this paragraph. Recruitment on in situ experiments suggest a gene recruitment, which I consider inaccurate, and makes it redundant considering the next part of the sentence. I think that "recruitment of regulatory genes" is incorrect, and recruitment could be used for proteins and other factors that actually are translocated between areas.

Results

1. "FGF signaling acts through MAPK and is regulated by sprouty genes in Lophophorates": While the experiment clearly demonstrates that sprouty, as a target gene for ERK, is affected by the treatment, no experiment is carried out to demonstrate or suggest that sprouty is a master regulator of mesoderm formation. This experiment demonstrates that there is no mesoderm (due to the treatment) and that sprouty (a target) is affected, but only by targeting sprouty one could make such a claim.
2. To understand in which step of development the expression of sprouty genes [...]": Stage of development 3.
"FGF signal interpretation via MAPK as shown in other organisms [...]": I suggest a change to "integration" or "modulate FGF transduction" or "modulate FGF signaling"

© 2021. Published by The Company of Biologists under the terms of the Creative Commons Attribution License (https://creativecommons.org/licenses/by/4.0/).

4. About the antibody staining: Because of the nature of the technique, activation of the gene can't be established. Therefore, "expression of" should be substituted by "activation of" [ERK, the pathway].
5. Orthologs and paralogs: In general, the pipeline seems correct. However, the authors used transcriptomes instead of genomes to find all the potential orthologs and paralogs in the three species. Certain disadvantages are derived from this approach, although I am aware of the possible difficulties here and the orthology work on the manuscript is robust. I wonder how the used transcriptomes were produced. Are all stages of development included so no early or late gene could be missed? Are different isoforms described for other groups?

Discussion aspects:
1. Is apical tuft different from the apical organ?
2. On the experiments for sprouty, there are several regions that remain unaffected. I wonder if the authors could discuss these results a little bit more; for example:
a. Are the domains in which P-ERK remains active activating the pathway by other ligands or effectors known;
or by contrast is just penetrance?
3. In relation to the sprouty paralogs, can the authors establish any correspondence between Terebratalia 2 and Phoronopsis 1, and Terebratalia 1 and Phonoropsis 2, given their patterns? Or this duplication is independent in both species and there's no such a relationship?
a. It's specially intriguing the ectodermal domain in just one of the paralogs. Are both mesodermal and ectodermal domains being affected by the treatment? Unfortunately, the WMISH for the phoronid is not very crisp.

Materials and methods

General comment: It's extremely valuable and much appreciated to have dependable and well-described methods for reproducibility purposes, and other's labs experiments, especially in emergent models. I strongly recommend that important details be provided.

1. Please, provide the size of the probes, the chosen regions that allowed to get specific probes in the case of 2 orthologs 2. Please, provide reference/catalog number/company of the reagents used

References

General comment: Some references in the main manuscript have a different format and lack the author list. In the supplemental references, the format is different from the main list.

Others:
1. After gastrulation, vegetal view is not accurate. Gastrulation defines the three axes; hence, this would correspond to a ventral view. I suggest to change this label in all gastrula+ images and text (Example Fig. S4 legend).
2. Change "Full projection" by "Z-projection"
3. 20 um, 50uM: It's not the abbreviation for micro-. In methods section, three different formats are used in the same line.
4. Line number is appreciated for review purposes.

Second revision

Author response to reviewers' comments

We thank the reviewers and the editor for their positive evaluation. We addressed their comments as following:

© 2021. Published by The Company of Biologists under the terms of the Creative Commons Attribution License (https://creativecommons.org/licenses/by/4.0/).

Reviewer comments:

Reviewer 1 Advance Summary and Potential Significance to Field:

As previously said,in their article entitled "FGF signalling induces mesoderm in members of Spiralia", Carmen Andrikou and Andreas Hejnol describe the expression pattern of genes known as mesodermal/muscle markers in other bilaterian animals in one brachiopod and one phoronid species. They then assess the role of FGF signalling in the formation of the mesoderm in these species and in another brachiopod species by looking at the expression of the receptor and ligand genes during development, and by using pharmacological treatments with SU5402. FGF has been shown to be required at different steps of mesoderm or mesodermal derivatives formation in diverse bilaterian phyla (deuterostomes and ecdysozoans) and the work presented here brings potentially some indications suggesting that this signalling pathway would also play similar although variable roles in members of the Spiralia group. The authors present many data, most of them technically sounds. The authors have answered most of my comments and have made a big effort by undertaking new experiments in order to test for the potential specificity of SU5402. However, I still have some concerns detailed below.

Reviewer 1 Comments for the Author:

1. The anti-dpERK1/2 immunostaining was undertaken only on two species over three. In the corresponding FigS10, the authors indicate that the pictures correspond to larva, but I believe they rather correspond to gastrula stage embryos. I feel the results are not really convincing. Although the nuclear signal is lost in treated embryos, the few nuclei labelled in control embryos do not seem to correspond to cells expressing FGFR. I know that immunostaining against dpERK1/2 is not efficient in many species and I would not present these results. The data on Sprouty genes expression is much more convincing to me, with the loss of expression in treated embryos observed early in N. anomala and Ph. harmeri, and later in T. transversa. Therefore, I think these results should be presented as a main figure, and before the analysis of the SU5402-treated embryos phenotype, not as the last results section (so before the "Perturbation of FGF signalling..." section).

Response: The reviewer is right; we were unable to undertake the anti-dpERK1/2 immunostaining in the third investigated species, *Ph. harmeri*. Since the reviewer feels that the results are not convincing enough, we decided to follow his/her recommendation and eliminate them from the manuscript. We also moved the analysis of the *sprouty* gene expression before the analysis of the SU5402-treated phenotypes (now Fig 5).

2. Concerning MAPK, in the main text and the figure legends, the authors are not analysing the expression of MAPK (MAPK is a big family with many members), they are analysing the translocation into the nucleus of the active form (diphosphorylated) of ERK1/2 which is described as the downstream effector of the MAPK pathway activated by FGFR.

Response: The reviewer is correct. Since we eventually eliminated the anti-dpERK1/2 experiments, we also eliminated this part from the text.

3. FigS7 is absolutely not informative

Response: We are sorry that the reviewer feels this way. We actually made Fig S7 following the suggestion of reviewer 3, who asked for a summary of the perturbation results and 3D reconstructions of the larvae. Therefore, we prefer to keep this figure.

4. In all the figures the insets are extremely small and sometimes covering the main picture. Moreover, panel labels are also often overlapping with the embryo, which renders the nice results very hard to see even at 300% zoom. This should really be modified.

Response: We have now increased the inset's size and made them as large as the main panels. For Figs 2, 3, 4 and 5 we moved them on the right side of the figures. For Fig 6 we needed to create a separate supplementary figure (Fig S8) due to space limitation, where we show the lateral views of the embryos (while in the main Fig 6 we only kept the dorsoventral views). In this way, the panels

© 2021. Published by The Company of Biologists under the terms of the Creative Commons Attribution License (https://creativecommons.org/licenses/by/4.0/).

labels are not overlapping anymore with the embryos.

5. Title: again, I see a problem with the use of “induction” in the title

Response: We changed the title to “FGF signaling acts on different levels of mesoderm development within Spiralia”.

6. Introduction page 3: “in T. transversa mesoderm originates... » I believe the mesoderm does not originate but is specified at the blastula stage.

Response: We have changed this sentence according to the reviewer’s suggestion.

7.Results, page 5, twice : “(c2-c3-c4)”: I believe it is rather” (cs2-cs3-cs4)”. Same think page 6

Response: The reviewer is right. We have now corrected these parts.

8. Results, page 11: “Moreover, an additional participation of FGF in mesoderm migration...”: I do not understand how, only based on coexpression of fgfr and twist, a function of FGF signal in mesoderm migration can be speculated.

Response: The reviewer is correct. Due to the possible involvement of FGF in mesoderm migration and differentiation in brachiopods, we hypothesized that a similar case might also occur in phoronids. The coexpression of *fgfr* and the mesodermal marker *twist* throughout the development provides a hint in favor of this hypothesis but it is definitely not proving it. An additional hint comes from the fact that the larvae treated from the gastrula stage possess some mesodermal cells, but these are neither migrated posteriorly nor differentiated as in the control larvae. We understand however that in order to test this hypothesis more experiments are needed and therefore we modified our sentence as following: “Moreover, an additional participation of FGF in mesoderm migration and differentiation (as seen in brachiopods) might occur due to the recovered but undifferentiated mesoderm in larvae treated from the gastrula stage as well as the observed coexpression of the mesodermal marker *twist* and *fgfr* throughout the development (Fig S11). Additional experiments are needed to test this hypothesis.”

9. Discussion section, page 15: “impaired gastrulation can occur either as a direct...”: impaired gastrulation is proposed to be due to impaired mesoderm formation. However, it is rather impaired mesoderm formation that results from gastrulation defects/blocking. I think the results should be discussed in this sense.

Response: Unfortunately, we cannot be certain whether the impaired gastrulation we witness is caused by a failure in mesoderm formation or vice versa. For example, we already know from previous studies in brachiopods (Marin Duran et al 2016) that it is the time and location of mesoderm specification which results to different gastrulation modes and blastoporal fates between brachiopod species (e.g., protostomy vs deuterostomy).
Moreover, we don’t know whether impaired gastrulation and the failure in mesoderm formation are correlated or independent events. For example, in vertebrates FGF signaling seems to have two distinct and separate roles: one earlier in specifying and maintaining axial and paraxial mesoderm and a later morphogenetic role in coordinating gastrulation movements (Dorey and Amaya 2010). Therefore, we prefer to leave the discussion open for the reader’s interpretation. We however, modified the paragraph as following:
“Moreover, they all showed impaired gastrulation to some degree. Impaired gastrulation can be either correlated with a failure in mesoderm formation or an indirect effect. For example, in *T. transversa,* where mesoderm is formed independently of FGF signaling, most likely the role of FGF is only morphogenetic, in orchestrating cell movements during gastrulation. However, in the other two species, where FGF is somehow involved in mesoderm formation, it is still unclear whether the observed failure in gastrulation movements after FGF inhibition is associated to the lack in mesoderm formation or is an independent event.”

10. Discussion section page 15 : “The expression of fgf8/11/18”: it is “fgf8/17/18”. Moreover, there are absolutely no evidence for a gradient and the sentence is really too speculative.

© 2021. Published by The Company of Biologists under the terms of the Creative Commons Attribution License (https://creativecommons.org/licenses/by/4.0/).

Response: We thank the reviewer for spotting this out. We have changed fgf8/11/18 to fgf8/17/18. Concerning the second point, the reviewer is correct as there is no evidence for a gradient in brachiopods. We therefore removed the word “gradient” from the sentence.
However, the relative spatial expression of *fgf8/17/18* and *fgfr* together with the SU5402 experiments suggest a possible link between posterior axial elongation and mesoderm differentiation of the brachiopod embryo and we believe that the comparison with vertebrates is interesting even if it doesn’t include a gradient.

11. Discussion section, page 16: MAPK, as I said before, corresponds in a fact to a multiplicity of pathways and precisions are needed. Moreover, MAPK pathways are activated by a wide range of signals, among which many RTKs (Receptor Tyrosine Kinase) that are not FGFRs, and bilaterians all possess many RTKs that might be using MAPK as a cytoplasmic output of their activation. Hence, the last sentence is a real shortcut that should be avoided.

Response: We have now eliminated the last sentence of this paragraph.

12. Materials and methods: for double fluorescent ISH, were only DIG-labelled probes used?

Response: The reviewer is correct, we also used DNP-labeled probes. We have now added this information in the materials and methods.

13.“dv” is used for dorsoventral view. But I believe it is or dorsal or ventral views. It should be specified more clearly in all the figures.

Response: The reason we have used “dorsoventral view” instead of dorsal or ventral view is because the images are focusing on the signal and not on the view itself, which sometimes results in views that are neither clear dorsal nor ventral views. Moreover, in the case of fluorescent images the embryos are not single images, but Z-projections made from several stacks and therefore the view is again not clearly dorsal or ventral.

14.I am not a native English speaker, but I think a little bit of editing is required.

Response: We have now gone through a round of English editing made by a native speaker.

15. uM or µM are used

Response: We have now changed uM to µM to ensure consistency.

16. Please add line numbers

Response: We have now added line numbers.

Reviewer 2 Advance Summary and Potential Significance to Field:

It was my misunderstanding that the author suggest conserved role of FGF signaling in the bilaterians.

Reviewer 2 Comments for the Author:

My comments were properly addressed in the revised manuscript. I like their additional experiment on the specificity of SU5402.

Response: We thank the reviewer for his/her valuable input and positive evaluation.

Reviewer 3 Advance Summary and Potential Significance to Field:
As before

© 2021. Published by The Company of Biologists under the terms of the Creative Commons Attribution License (https://creativecommons.org/licenses/by/4.0/).

Reviewer 3 Comments for the Author:

Minor revisions:

Abstract

1. "FGF signaling is involved in mesoderm induction in members of deuterostomes (e.g. tunicates, hemichordates) [...]": I think that other examples could be better for defining Deuterostomia.

Response: We have used specifically these two examples because the role of FGF signaling in mesoderm induction is only demonstrated in these two deuterostome groups.

2. "Moreover, inhibitor experiments demonstrate that FGF [...]": I suggest changing the name of the approach, i.e "the use of the inhibitor X".

Response: We have changed this sentence according to the reviewer's suggestion.

Introduction

1. "Mesoderm is an embryonic germ layer of bilaterians that gives rise to tissues residing between the ectoderm and endoderm, such as coeloms and muscles (Hyman, 1951; Ruppert, 1991). The way mesoderm is formed varies between embryos of different species. Mesoderm can originate in an epithelial mode by outpouchings of the invaginating endoderm (for example in most deuterostomes (Franz, 1924; Hennig, 1984; Hyman, 1955; Hyman, 1959; Swalla, 1993) and two clades of protostomes, the Chaetognatha (Hertwig, 1896; Kapp, 2000; Matus et al., 2006) and the Brachiopoda (Conklin, 1902; Kowalevsky, 1874; Plenk, 1913)), or in a mesenchymal mode from one or more precursor cells that internalize during gastrulation (for example in spiralian species, [...]": Coeloms are not tissue but cavities, generated by the gastrulation process, but not by mesoderm itself. On the other hand, "epithelial and mesenchymal" are not defined types of gastrulation, as invagination and delamination are. I think that a more proper term is required here.

Response: We changed the sentence modifying the terminology of gastrulation types as following: " Mesoderm can originate by outpouchings of the invaginating endoderm (for example in most deuterostomes (Franz, 1924; Hennig, 1984; Hyman, 1955; Hyman, 1959; Swalla, 1993) and two clades of protostomes; the Chaetognatha (Hertwig, 1896; Kapp, 2000; Matus et al., 2006) and the Brachiopoda (Conklin, 1902; Kowalevsky, 1874; Plenk, 1913)). Alternatively, mesoderm can form by delamination of one or more precursor cells that internalize during gastrulation (for example in spiralian species, where the source of mesoderm can be of endodermal (e.g. the micromere 4d) and ectodermal origin (e.g. micromeres from the animal pole/anterior end of the blastopore) (summarized in Henry and Martindale, 1999; Kozin and Kostyuchenko, 2016; Lambert, 2008; Lyons and Henry, 2014) and in ecdysozoans, where mesoderm originates either from internalization of vegetal endomesodermal cells (Martin- Duran and Hejnol, 2015; Sulston et al., 1983), or from cells of the blastoderm (Eriksson and Tait, 2012; Hartenstein et al., 1985))."

2. "This molecular conservation has been commonly used as an argument for the homology of this germ layer [...]": This paragraph didn't mention molecules. I consider "mechanisms" to be more accurate.

Response: Indeed, we don't mention molecules by names in this paragraph. However, we do refer to similarities in the molecular underpinnings. The argument on mesoderm homology has been heavily based on shared sets of transcription factors and not so much on general mechanisms of development (that would imply also morphogenesis etc., where different animals show remarkable differences in the way they form mesoderm, as stated in the introduction). We have now changed the sentence to: "These molecular similarities have been commonly used as an argument for the homology of this germ layer."

3. "TVC and tail muscle in tunicates [...]": This abbreviation hasn't been defined before

Response: The reviewer is correct; we have now defined the abbreviation of TVC in the text.

© 2021. Published by The Company of Biologists under the terms of the Creative Commons Attribution License (https://creativecommons.org/licenses/by/4.0/).

4. "These data show that in all three organisms, mesoderm development exhibit differences not only in the recruitment of regulatory genes but also in their temporal and spatial expression profiles, suggesting diverse underlying patterning mechanisms [...]": I suggest a review of this paragraph. Recruitment on in situ experiments suggest a gene recruitment, which I consider inaccurate, and makes it redundant considering the next part of the sentence. I think that "recruitment of regulatory genes" is incorrect, and recruitment could be used for proteins and other factors that actually are translocated between areas.

Response: Gene recruitment is a term for the placement of a gene under a regulatory system. In this case, we refer to a set of transcription factors that are utilised in order to form mesoderm. We have now changed the sentence to: "These data show that in all three organisms, mesoderm development exhibit differences not only in the recruitment of transcription factors but also in their temporal and spatial expression profiles, suggesting diverse underlying patterning mechanisms."
The recruitment of these transcription factors as part of the mesoderm formation toolkit is not the same as their spatial or temporal expression profiles, in which we refer in the next part of the sentence. Therefore, we think that both parts of the sentence are needed.

Results

1. "FGF signaling acts through MAPK and is regulated by sprouty genes in Lophophorates": While the experiment clearly demonstrates that sprouty, as a target gene for ERK, is affected by the treatment, no experiment is carried out to demonstrate or suggest that sprouty is a master regulator of mesoderm formation. This experiment demonstrates that there is no mesoderm (due to the treatment) and that sprouty (a target) is affected, but only by targeting sprouty one could make such a claim.

Response: The reviewer is right; these experiments were conducted as an effort to show SU5402 specificity and not to suggest that sprouty is a master regulator of mesoderm formation. However, the fact that the expression of *sprouty* genes resemble *fgfr* gene expression both spatially and temporally, suggests that it might be downstream of FGF signaling pathway. We have now changed the title of this paragraph to "*Sprouty* genes are downstream of FGF signaling pathway in lophophorates" and also modified similar parts of the text, accordingly.

2. To understand in which step of development the expression of sprouty genes [...]": Stage of development

Response: We have now corrected this sentence according to the reviewer's suggestion.

3. "FGF signal interpretation via MAPK as shown in other organisms [...]": I suggest a change to "integration" or "modulate FGF transduction" or "modulate FGF signaling"

Response: We have decided to eliminate the anti-dpERK1/2 immunostaining data after the suggestions of the Editor and Reviewer 1. Therefore, this sentence is now eliminated from the text.

4. About the antibody staining: Because of the nature of the technique, activation of the gene can't be established. Therefore, "expression of" should be substituted by "activation of" [ERK, the pathway].

Response: As mentioned above, we have now eliminated the anti-dpERK1/2 immunostaining data.

5. Orthologs and paralogs: In general, the pipeline seems correct. However, the authors used transcriptomes instead of genomes to find all the potential orthologs and paralogs in the three species. Certain disadvantages are derived from this approach, although I am aware of the possible difficulties here and the orthology work on the manuscript is robust. I wonder how the used transcriptomes were produced. Are all stages of development included so no early or late gene could be missed? Are different isoforms described for other groups?

Response: We understand the reviewer's concern. The transcriptomes were produced by mixing

© 2021. Published by The Company of Biologists under the terms of the Creative Commons Attribution License (https://creativecommons.org/licenses/by/4.0/).

embryos from all stages of development; therefore, we are confident that early or late gene were not missed. Different isoforms of some genes (e.g., FGFR) are described for some animal groups, however without genome availability we are not able to perform a similar analysis in out investigated species.

Discussion aspects:

1. Is apical tuft different from the apical organ?

Response: Yes, they are indeed different. An apical tuft consists of a group of external long sensory cilia and is usually part of an apical organ, which is in many cases composed out of internal or epidermal sensory cells.

2. On the experiments for sprouty, there are several regions that remain unaffected. I wonder if the authors could discuss these results a little bit more;

Response: We have now extended the description of *sprouty* expression in the SU5402 treatments, as following:
“When we tested the expression of *sprouty* genes in larvae treated from the blastula stage, a severe reduction in the mesoderm and mesodermal derivatives was witnessed in all three species (Fig. 5Ba-j’). Additionally, *Ph. harmeri* exhibited a loss of ectodermal expression (Fig. 5Be-f’). When larvae were treated from the gastrula stage, the mesodermal expression of *sprouty* was partly recovered in *Ph. harmeri* and *T. transversa* (Fig. 5Ba’’-f’’) but this was not the case for *N. anomala* (Fig. 5Bi’’-j’’). Ectodermal expression was also recovered in *Ph. harmeri* (Fig. 5Be’’-f’’).”

a. Are the domains in which P-ERK remains active activating the pathway by other ligands or effectors known; or by contrast is just penetrance?

Response: Both interpretations are possible. The expression domain of FGFR is similar to the one of P-ERK but we cannot rule out that other pathways (e.g., RTKs) are also expressed in a similar manner. Nevertheless, as mentioned above, these experiments are now eliminated from the manuscript, as suggested by the Editor and Reviewer 1.

3. In relation to the sprouty paralogs, can the authors establish any correspondence between Terebratalia 2 and Phoronopsis 1, and Terebratalia 1 and Phonoropsis 2, given their patterns? Or this duplication is independent in both species and there's no such a relationship?

Response: In the phylogeny (Fig. S1) *N. anomala* sprouty1, *Ph. harmeri* sprouty1, and *T. transversa* sprouty1 group together, *Ph. harmeri* sprouty2, and *T. transversa* sprouty2 however likely emerged from an independent duplication.

a. It's specially intriguing the ectodermal domain in just one of the paralogs. Are both mesodermal and ectodermal domains being affected by the treatment? Unfortunately, the WMISH for the phoronid is not very crisp.

Response: The reviewer is correct, unfortunately the WMISH was not very crisp for *Ph. harmeri*. In *Ph. harmeri* both mesodermal and ectodermal domains were affected when larvae were treated from the blastula stage, however in the treatment from the gastrula stage, the expression was partly recovered. This was not the case for *T. transversa,* which retained an ectodermal expression of *sprouty* in both treatments. We now provide further explanation in the text.

Materials and methods

General comment: It's extremely valuable and much appreciated to have dependable and well-described methods for reproducibility purposes, and other's labs experiments, especially in emergent models. I strongly recommend that important details be provided.

1. Please, provide the size of the probes, the chosen regions that allowed to get specific probes in the case of orthologs

© 2021. Published by The Company of Biologists under the terms of the Creative Commons Attribution License (https://creativecommons.org/licenses/by/4.0/).

Response: The size of the probes and the chosen regions are now provided in Table S2.

2. *Please, provide reference/catalog number/company of the reagents used*

Response: The reference/catalog number/company of the reagents used in this study are now added in the material and methods section.

References

General comment: Some references in the main manuscript have a different format and lack the author list. In the supplemental references, the format is different from the main list.

Response: We believe that the reviewer is referring to consecutive references that contain the same author list. The output of the library in these cases are references lacking the author list, for example:
***Altenburger**, A. **and Wanninger**, A. (2009). Comparative larval myogenesis and adult myoanatomy of the rhynchonelliform (articulate) brachiopods Argyrotheca cordata, A. cistellula, and Terebratalia transversa. Front. Zool. **6**.*
***----** (2010). Neuromuscular development in Novocrania anomala: evidence for the presence of serotonin and a spiralian-like apical organ in lecithotrophic brachiopod larvae. Evol. Dev. **12**, 16-24.*
We have now corrected that.

Concerning the supplemental references, we have now changed the supplementary references output and made it as in the main text.

Others:
1. *After gastrulation, vegetal view is not accurate. Gastrulation defines the three axes; hence, this would correspond to a ventral view. I suggest to change this label in all gastrula+ images and text (Example Fig. S4 legend).*

Response: The only visible axis in the blastula and early gastrula is the animal/vegetal axis. Therefore, we only use “vegetal” view for these two developmental stages, while from the late gastrula onwards -where the anterior/posterior and dorsal/ventral axis get established- we switch to “dorsoventral” views. It is true that often the later “ventral view” corresponds to an earlier “vegetal” view, but since this event occurs at a later stage of development we would prefer to keep the labelling as it is.

2. *Change "Full projection" by "Z-projection"*

Response: We have now changed it according to the reviewer’s suggestion.

3. *20 um, 50uM: It's not the abbreviation for micro-. In methods section, three different formats are used in the same line.*

Response: We have now changed uM to µM to ensure consistency.

4. *Line number is appreciated for review purposes.*

Response: We have now added line numbers.

Third decision letter

MS ID#: DEVELOP/2020/196089

MS TITLE: FGF signaling acts on different levels of mesoderm development within Spiralia

© 2021. Published by The Company of Biologists under the terms of the Creative Commons Attribution License (https://creativecommons.org/licenses/by/4.0/).

AUTHORS: Carmen Andrikou and Andreas Hejnol
ARTICLE TYPE: Research Article

I am happy to tell you that your manuscript has been accepted for publication in Development, pending our standard ethics checks.

© 2021. Published by The Company of Biologists under the terms of the Creative Commons Attribution License (https://creativecommons.org/licenses/by/4.0/).
